# Barriers, Facilitators and Priorities for Implementation of WHO Maternal and Perinatal Health Guidelines in Four Lower-Income Countries: A GREAT Network Research Activity

**DOI:** 10.1371/journal.pone.0160020

**Published:** 2016-11-02

**Authors:** Joshua P. Vogel, Julia E. Moore, Caitlyn Timmings, Sobia Khan, Dina N. Khan, Atkure Defar, Azmach Hadush, Marta Minwyelet Terefe, Luwam Teshome, Katherine Ba-Thike, Kyu Kyu Than, Ahmad Makuwani, Godfrey Mbaruku, Mwifadhi Mrisho, Kidza Yvonne Mugerwa, Lisa M. Puchalski Ritchie, Shusmita Rashid, Sharon E. Straus, A. Metin Gülmezoglu

**Affiliations:** 1 UNDP/UNFPA/UNICEF/WHO/World Bank Special Programme of Research, Development and Research Training in Human Reproduction (HRP), Department of Reproductive Health and Research, World Health Organization, Headquarters, Geneva, Switzerland; 2 Knowledge Translation Program, Li Ka Shing Knowledge Institute, St. Michael’s Hospital, Toronto, Ontario, Canada; 3 Health System and Reproductive Health Research Directorate, Ethiopian Public Health Institute, Addis Ababa, Ethiopia; 4 WHO Country Office, Addis Ababa, Ethiopia; 5 Maternal and Child Health Directorate, Ministry of Health, Addis Ababa, Ethiopia; 6 Independent consultant, Yangon, Myanmar; 7 Burnet Institute, Melbourne, Australia; 8 Department of Medicine, The University of Melbourne, Melbourne, Victoria, Australia; 9 Ministry of Health, Community Development, Gender, Elderly and Children, Dar-es-Salaam, United Republic of Tanzania; 10 Ifakara Health Institute, Dar-es-Salaam, United Republic of Tanzania; 11 Makerere University, Kampala, Uganda; 12 Department of Emergency Medicine, University Health Network, Toronto, Canada; University of Exeter, UNITED KINGDOM

## Abstract

**Background:**

Health systems often fail to use evidence in clinical practice. In maternal and perinatal health, the majority of maternal, fetal and newborn mortality is preventable through implementing effective interventions. To meet this challenge, WHO’s Department of Reproductive Health and Research partnered with the Knowledge Translation Program at St. Michael’s Hospital (SMH), University of Toronto, Canada to establish a collaboration on knowledge translation (KT) in maternal and perinatal health, called the GREAT Network (Guideline-driven, Research priorities, Evidence synthesis, Application of evidence, and Transfer of knowledge). We applied a systematic approach incorporating evidence and theory to identifying barriers and facilitators to implementation of WHO maternal heath recommendations in four lower-income countries and to identifying implementation strategies to address these.

**Methods:**

We conducted a mixed-methods study in Myanmar, Uganda, Tanzania and Ethiopia. In each country, stakeholder surveys, focus group discussions and prioritization exercises were used, involving multiple groups of health system stakeholders (including administrators, policymakers, NGOs, professional associations, frontline healthcare providers and researchers).

**Results:**

Despite differences in guideline priorities and contexts, barriers identified across countries were often similar. Health system level factors, including health workforce shortages, and need for strengthened drug and equipment procurement, distribution and management systems, were consistently highlighted as limiting the capacity of providers to deliver high-quality care. Evidence-based health policies to support implementation, and improve the knowledge and skills of healthcare providers were also identified. Stakeholders identified a range of tailored strategies to address local barriers and leverage facilitators.

**Conclusion:**

This approach to identifying barriers, facilitators and potential strategies for improving implementation proved feasible in these four lower-income country settings. Further evaluation of the impact of implementing these strategies is needed.

## Introduction

Globally, all healthcare systems face challenges to improving quality of care, as observed by failures to optimally use best available evidence.[1–5] While the generation of new evidence through clinical research is a necessary step, health systems often fail to ensure that evidence is used in routine decision-making and clinical practice.[6] This has led to a growing body of research on how to achieve effective, sustained implementation of evidence-based products (such as guidelines). Knowledge translation (KT) is one of a host of terms (e.g., implementation, knowledge transfer) used to describe “a dynamic and iterative process that includes the synthesis, dissemination, exchange and ethically sound application of knowledge to improve health, provide more effective health services and products, and strengthen the healthcare system.”[6]

Within maternal and perinatal health, there are many examples of failures to implement interventions that are known to be effective.[7] Globally, an estimated 289,000 maternal deaths, 2.6 million stillbirths and 2.8 million newborn deaths occur each year.[8–10] These deaths are largely preventable, and highlight the importance of ensuring that high-quality care is available for every woman and newborn throughout pregnancy, childbirth and the postpartum period.[11] In this article, we aim to describe barriers, facilitators and strategies for implementing WHO maternal health guidelines identified in a mixed-methods study in four low and middle-income countries (LMICs).

### The GREAT Network

The World Health Organization (WHO) uses a rigorous process for guideline development, based on the standards of the WHO Guideline Review Committee.[12] The Department of Reproductive Health and Research (RHR) at WHO issues recommendations on a range of reproductive health topics.[13] However, since guideline development and dissemination alone is not enough to change behaviour and improve outcomes,[14,15] there is a clear need for effective strategies to support implementation of these guidelines into practice globally. To meet this challenge, RHR partnered with the Knowledge Translation Program at St. Michael’s Hospital, University of Toronto, Canada to establish a collaboration on KT in maternal and perinatal health, called the GREAT Network (Guideline-driven, Research priorities, Evidence synthesis, Application of evidence, and Transfer of knowledge).[16]

**Fig 1:**
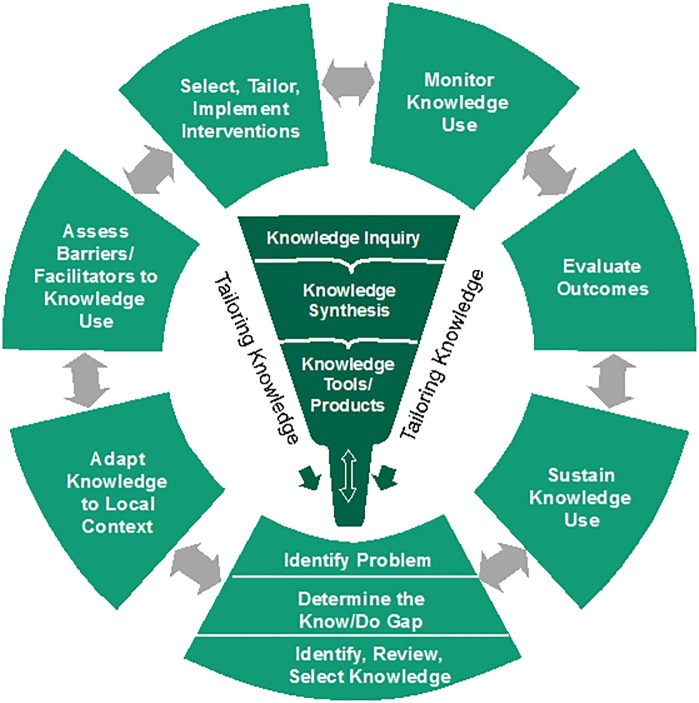
Knowledge-to-Action Cycle (Reproduced with permission from[38]). Reprinted from Straus SE, Tetroe J, Graham I. Knowledge translation in health care: moving from evidence to practice. 2nd ed. BMJ Books, Wiley, 2013. under a CC BY license, with permission from Wiley, original copyright 2013.

The GREAT Network uses a systematic approach based on evidence and theory to support LMICs in the implementation of maternal and perinatal health guidelines. The Network’s activities are guided by the Knowledge-to-Action (KTA) cycle (Fig 1), a process model which presents the steps involved in bringing research to practice. The theoretical elements exist on two levels: first, the KTA was developed based on a review of over 30 behavior change theories and frameworks[17]; second, the process of selecting implementation strategies to address identified barriers and facilitators to change involves incorporating behaviour change theory, for example the theory of planned behaviour or the capability, opportunity, motivation—behaviour theory.[17] The KTA also emphasizes the need to consider evidence at each step along the process. The GREAT Network conducted a mixed-methods pilot study on the determinants of implementation of maternal health guidelines in Kosovo in 2012.[18]. The process was adapted and expanded into a multi-country, mixed methods research activity described below.

This project focused on the first steps of the action cycle of the KTA (Fig 1), namely: adaptation of knowledge to the local context (i.e. adapting WHO guidelines to each country setting), assessing barriers and facilitators to knowledge use, and selecting and tailoring implementation strategies to local contexts. The study utilized an integrated KT approach, where countries’ and relevant WHO maternal health guidelines were identified based on in-country needs. The premise of integrated KT is that end users should be involved in research and implementation throughout the process so that the activities and outcomes directly address their needs.[19] This article describes the methods, findings and lessons learned from these activities. In subsequent phases, local working groups will progress through the action cycle steps of tailoring, implementing, monitoring, evaluating and sustaining the use of guidelines in their local contexts.

## Methods

A mixed-methods (qualitative and quantitative) study was conducted in each of the four participating countries (Myanmar, Uganda, Tanzania and Ethiopia), adapted from the methodology developed in the Kosovo pilot study.[18] For each country, Phase 1 comprised a three-step process: establishing a multi-stakeholder group in each country to identify local maternal health guideline priorities (Step 1); conducting mixed methods research in the participating country to identify priorities, barriers, and facilitators to guideline implementation (Step 2); and developing an implementation plan, that incorporates contextualized implementation strategies, in accordance with findings from Step 2 (Step 3). Steps 2 and 3 were conducted during a 2-day in-person workshop with relevant local stakeholders. All steps were conducted with local partners (who were co-principal investigators). Subsequent implementation activities are currently ongoing, with support from the GREAT Network.

We developed a generic protocol describing the methods and outputs for this activity, allowing for local adaptation where required. The study protocol was approved for technical content by the WHO Research Project Review Panel; the WHO Ethics Review Committee reviewed the project and deemed it exempt from review. Relevant local approvals were obtained (where required) in each country (see below).

### Setting

Participating countries were identified through purposive sampling. Within each country, activities related to a specific guideline implementation challenge, identified as a national priority at the time of the activities. In Myanmar, the activity was conducted in the context of a Ministry of Health initiative to improve the coverage of basic maternal and newborn healthcare nationwide, with particular emphasis on task-shifting from midwives to auxiliary midwives (AMWs). Uganda, Tanzania and Ethiopia were identified as priority countries within the UN Commission on Life Saving Commodities for Women and Children, which aims to improve access to 13 essential commodities (including the maternal health commodities oxytocin, misoprostol and magnesium sulfate). In-country activities were conducted in Myanmar in June 2014, Uganda in August 2014, Tanzania in November 2014 and Ethiopia in May 2015.

### Design

#### Step one: Identifying and establishing a local working group

Using an integrated KT approach, the first step was to identify key stakeholders. In each country, local investigators were identified via existing networks. Composition of the local working groups generally included 3–5 individuals from maternal health research organizations, clinical obstetrics, Ministry of Health, civil society and the WHO country office. A series of virtual meetings were held with this group to review KT principles, discuss local implementation priorities and to plan activities. A consensus approach was used to select the guideline/s of interest, based on their knowledge of relevant local initiatives and priorities and with informal consultation with other local stakeholders (such as Ministry of Health, United Nations (UN) agency or university staff).

#### Step two: Mixed methods research

In each country, an anonymous survey was conducted to obtain understanding of key priorities related to the WHO guidelines, used to inform discussions and deliberations at the in-person workshops (see below). Surveys differed slightly between countries (S1–S4 Files) for individual surveys used in each country), however all included questions on: the respondent’s demographic and professional information, their current role and responsibilities, and perceived maternal health guideline priorities or factors affecting their uptake. In Myanmar, participants were asked to rate their agreement on the extent to which a list of factors were barriers to the use of the WHO task-shifting guidelines in their setting. In Uganda, Tanzania and Ethiopia, participants were asked to prioritize recommendations for implementation from the selected WHO guidelines.

The local working group created a list of relevant local stakeholders (minimum 50), based on existing networks and websites of relevant organizations. This stakeholder list included healthcare providers (such as obstetricians, paediatricians, midwives and nurses), policymakers, healthcare administrators, program managers, researchers, non-governmental organization (NGO) staff, UN agency staff, professional association representatives, and other stakeholders from relevant local organizations. Participants were invited to complete a survey (the survey stated that consent was implied upon completion of the survey) and reminders were sent at approximately two and four weeks.[20]. Both paper and online surveys were used to maximize responses and ensure that those with limited or no web access were not unduly disadvantaged from participating (both surveys used same questions).

The two-day in-person workshops aimed to incorporate perspectives from a diverse range of healthcare system stakeholders. The objectives of the workshop were to utilize these multiple perspectives to identify barriers and facilitators to guideline implementation, select priority recommendations, and identify potential implementation strategies to improve guideline uptake. Primary data collection occurred during the workshops, including focus group discussions (FGDs), an anonymized individual ranking exercise (using an electronic audience response system), and small and large group discussions. Workshop participants were purposively sampled (using the stakeholder list described above), with the aim of recruiting 20–35 participants per country. To ensure representation from across the healthcare system, healthcare administrators, policymakers, non-governmental organization staff, representatives from professional associations, frontline healthcare providers, and healthcare system researchers/academics were identified and recruited. Individuals from different levels of the healthcare system (eg: regional, district and facility level) were identified. A particular emphasis was placed on recruiting relevant opinion leaders and decision makers, as well as ensuring participants from rural and urban areas. Prior to the workshop, participants received information on the objectives of the workshop, as well as a summary of the guideline(s) (translated where necessary).

The workshop was co-chaired by local working group members and international partners. Translators were available as required. Day 1 included a presentation of key principles of knowledge translation and the WHO guideline development process, as well as national maternal and newborn health indicators and priorities. Findings of the pre-workshop survey were presented, to provide additional information from other stakeholders for workshop participants to consider when deliberating priority recommendations for implementation. Subsequently, two to four in-person FGDs (of 6 to 8 people each) were held, lasting up to 2 hours each. Each FGD was co-facilitated by a nominated workshop participant and a researcher from WHO or St Michael’s Hospital. A customized FGD guide was developed by the research team and was adapted for each country workshop (S1–S4 Files). FGDs were organized by cadre to facilitate disclosure by participants. The objectives of the FGDs were to identify and explore priorities, barriers and facilitators to the adaptation and implementation of relevant guideline recommendations. Informed consent was obtained prior to the commencement of FGDs. The FGDs were digitally recorded and field notes taken for analysis.

On Day 2, findings from Day 1 activities were fed back to participants, including a preliminary analysis of priorities, barriers and facilitators. Participants were asked to complete an anonymous individual ranking exercise (via the electronic audience response system) to identify the extent of consensus and to prompt reflection for further deliberation. With this system, each participant is able to vote anonymously in real time with results presented immediately. The ranking exercise was based on the modified Delphi technique, which is used to gather input from participants who may have differing views and perspectives. Participants ranked priority recommendations in terms of relevance and feasibility for implementation in the local context. Consistent with the RAND appropriateness method,[21] ratings were based on a nine-point Likert scale. When responses for a given recommendation were highly disparate, large group discussion took place and responses were re-ranked with the aim of reaching a higher level of agreement.

#### Step Three: Developing an implementation plan

On Day 2, participants reconvened in small groups to identify potential strategies for implementing the prioritized recommendations in their practice settings. Participants were encouraged to link proposed implementation strategies back to the underlying barriers that would be addressed, and leverage identified facilitators. Small group discussions were co-facilitated by an experienced researcher and a workshop participant nominated by the group. Deliberations were digitally recorded and field notes were taken for analysis.

#### Analysis

Descriptive statistics were calculated on quantitative survey data. For the qualitative data, all FGDs and small group discussions were audio taped and transcribed. Qualitative analysis of the transcripts and field notes was performed independently by two qualitative analysts using a thematic content analysis approach.[22] First, the analysts familiarized themselves with the data to develop initial coding themes. Second, these themes were further refined into categories that were ultimately used to develop a coding framework. Third, all transcripts were then coded by the analysts independently using the revised framework. Inter-rater reliability was compared once all transcripts were coded using percentage agreement; any discrepancies (i.e., < 80% agreement) between the analysts were reconciled through discussion.[23] Analysis was conducted using NVivo 10 software. A technical report for each country activity (containing a summary of quantitative survey data, and the qualitative analysis) was disseminated to workshop participants and other relevant stakeholders (S1–S4 Files).

## Results

A summary of key demographic and health indicators for each country, as well as the WHO guideline/s selected for these activities, is shown in Table 1. These are four countries of diverse context, however all have high maternal and perinatal mortality.The activity in Myanmar focused on recommendations on task-shifting for maternal and newborn health interventions from midwives to AMWs.[24] Activities in Uganda and Tanzania considered recommendations from four WHO maternal health guidelines (WHO recommendations for the prevention and treatment of postpartum haemorrhage; WHO recommendations for the prevention and treatment of pre-eclampsia and eclampsia; WHO recommendations for augmentation of labour; and WHO recommendations for induction of labour).[25–28] The activity in Ethiopia considered WHO recommendations on the prevention and treatment of postpartum haemorrhage.[25] A detailed description of study findings (including survey questions and results, and synthesized findings of FGDs) is provided in the technical reports for each country (S1–S4 Files) and an overview of key per-country findings is reported below.

**Table 1:** Summary of indicators and guideline implementation research activities and outcomes in four countries.

	Myanmar	Uganda	United Republic of Tanzania	Ethiopia
**Overarching objective at national level**	Improve coverage of maternal and newborn healthcare using task-shifting within midwifery and auxiliary midwifery	Improve access to and use of maternal health commodities (oxytocin, misoprostol and magnesium sulfate)		
**Key country level health indicators**				
**Population (2015 estimate)**[39]	53 897,000	39 032,000	53,470,000	99,391,000
**Skilled birth attendance rate (latest estimate)** [40]	70.6% (2010)	57.4% (2011)	48.9% (2010)	10.0% (2011)
**Maternal mortality ratio (2015 estimates)** [41]	178 per 100,000	343 per 100,000	398 per 100,000	353 per 100,000
**Guideline implementation activities and priorities**				
**WHO guideline/s of interest**	Recommendations on task-shifting maternal and newborn health interventions from midwives to auxiliary midwives	prevention and treatment of PPH prevention and treatment of pre-eclampsia and eclampsia augmentation of labour induction of labour	prevention and treatment of PPH prevention and treatment of pre-eclampsia and eclampsia augmentation of labour induction of labour	prevention and treatment of PPH
**Date of in-country activities**	June 2014	Aug 2014	Nov 2014	May 2015
**Number of workshop attendees**	42	34	32	19
**Factors affecting implementation:**				
**Health system level**	Shortage of MWs and AMWs Available resources Accountability and monitoring Policies and political context	Access to resources, drugs, equipment and supplies Drug procurement, distribution and management Purchasing and supply chain management Safety and use Medical indications Human resources Access to site-specific data Accountability and monitoring Documentation Regulation Policies	Access to resources, including drugs and drug distribution system, equipment, supplies and human resources Continuity of care Monitoring and evaluation Policies Dissemination of guidelines	Access to resources, including drugs, equipment, supplies, and human resources Drug procurement, distribution, management Data collection & monitoring Policies & incentives Readiness for change Guidelines & protocols
**Health provider level**	Roles and capacity of AMWs and MWs Education, continuing education and quality of training Willingness, buy-in and motivation Relationships between health cadres	Beliefs, attitudes and buy-in Knowledge and skills Training, coaching, mentorship and professional development Authorized roles	Beliefs, attitudes and buy-in Knowledge, skills and self-efficacy Training, mentoring and professional development	Beliefs, attitudes, buy-in Knowledge & skills Training & supportive supervision Role definition
**Woman / community level**	Community/patient perceptions of AMW and MW roles Cultural practices and health-seeking behaviours	Traditional beliefs and perceptions of healthcare services Knowledge and awareness Socioeconomic status	Health-seeking behaviour and preferences for care Community health care workers as champions Socioeconomic status	Traditional beliefs Knowledge & awareness Access to healthcare services
**Prioritized recommendations for implementation**	Administration of misoprostol to treat PPH before referral Management of puerperal sepsis with oral antibiotics Performance of neonatal resuscitation	The use of uterotonics for the prevention of PPH during the third stage of labour for all births Encouraging the adoption of mobility and upright position during labour in women at low risk The use of oral misoprostol for labour augmentation is not recommended.	In settings where oxytocin is unavailable, the use of other injectable uterotonics (ergometrine/ methylergometrine) or oral misoprostol for prevention of PPH Uterotonics for the prevention of PPH during the third stage of labour for all births. Magnesium sulfate for the prevention of eclampsia in women with severe pre-eclampsia Magnesium sulfate for treatment of women with eclampsia Active phase partograph with a four-hour action line for monitoring progress of labour.	Use of uterotonics for the prevention of PPH during the third stage of labour Late cord clamping (performed after 1 to 3 minutes after birth) for all births while initiating simultaneous essential newborn care. Postpartum abdominal uterine tonus assessment for early identification of uterine atony for all women. Uterine massage for the treatment of PPH. Uterine packing not recommended for the treatment of PPH due to uterine atony after vaginal birth.
**Potential implementation strategies for priority recommendations**				
**Priority implementation activities for next steps**	Target AMWs in rural areas Proper training and education of multiple cadres, especially AMWs, focused on above recommendations. Training can increase trust and buy-in across all levels, and improve perceptions about the roles of midwives (MWs) and AMWs. Consider reviewing and defining AMW roles in terms of how they are selected, trained, retained, regulated, and supervised Obtain policymaker buy-in and a push for policy changes to permit task shifting Engage policymakers and professional organizations with evidence briefs; Revise policies related to drug administration and distribution; Get financial commitments to provision of drugs and equipment to AMWs; Institute regulatory oversight of AMWs	Need for review of current drug procurement and monitoring practices to address drug shortages and expirations Conduct further research to better understand how misoprostol can be safely used in the community, extent and types of misoprostol misuse, and how to improve use in health facilities. Results could, in turn, support changes to policy. Recruit more physicians and MWs, particularly in rural/remote areas; infrastructure (e.g., housing for healthcare workers) and incentives are needed Eliminate current recruitment ban on hiring of physicians and MWs to improve frontline capacity to implement recommendations Create more formal linkages between healthcare facilities and village health teams to better coordinate and standardize care Increase awareness about harms and benefits of recommendations (e.g., benefits of a labour companion; medical causes of eclampsia). Could be achieved through strategies and activities directed at patients and the wider community (e.g., radio/SMS campaigns, community talks/meetings) Train staff in use of prioritized interventions	Improve national drug ordering and monitoring, including accountability measures for timely request and reporting, and implementing cost-sharing programs. Ensure access to equipment (eg: refrigerators, gloves and blood pressure cuffs). Budgeting can be improved via a Comprehensive Council Health Plan. Implement strategies to recruit and retain staff, focusing on rural areas Cross-train existing staff in maternal health so they can be re-distributed as needed Increase opportunities for training, with more focus on pre- and in-service training. Training should be competency- based include continuing medical education, supportive supervision and mentorship programs train and promote an interprofessional, collaborative healthcare team model to improve attitudes, buy-in, and provider confidence Create more formal linkages between the levels of facilities to better coordinate and standardize maternal healthcare. Opportunities to form linkages through technology (e.g., telemedicine) currently being piloted Widely disseminate guidelines, through strategies such as mass media campaigns, educational materials and community champions.	Create a multi-disciplinary guideline implementation working group within the Ministry of Health maternal health case team. Adapt WHO maternal health guideline for Ethiopian context using ADAPTE process. Create standard protocols on how to implement the guideline recommendations and distribute to facilities for onsite guidance. Protocols should be user-friendly, ready-to-use, and visible to act as reminders for HCWs. Select and implement priority clinical indicators Establish a mentorship program at the facility level between junior and senior HCWs to provide technical support and supportive supervision on implementation Establish an interdisciplinary quality improvement team (e.g., including physicians, midwives, administrators) at each healthcare facility to identify priority areas for practice improvement Design and conduct evaluation of implementation activities Identify strategies to improve and standardize the benefits package offered to HCWs across all regions Conduct evaluation of the Health Extension Worker Program Evaluate the Maternity Waiting Home initiative, which is currently being used in some remote areas to mitigate transport barriers

### Myanmar

A total of 31 people completed the pre-workshop survey and 97% agreed that task shifting to AMWs is a priority in Myanmar. In the survey, the three most prevalent barriers to implementation of the task-shifting recommendations were: 1) the need for training, retraining and supportive supervision of AMWs; 2) the need for clear policy on AMW roles and responsibilities; and 3) community preferences for different cadres of health workers.

Forty-two participants attended the workshop, which included six AMWs and four MWs. Four FGDs were conducted, stratified by role and/or level of the healthcare system (AMW, midwives (MW), Ministry of Health staff at central and township level, and a mixed group of UN and non-governmental stakeholders and representatives of professional organizations) and focused on the feasibility of implementing the task-shifting recommendations in Myanmar.

Participants in FGDs discussed factors affecting implementation of AMW-specific task-shifting recommendations. At the level of the healthcare system, factors included: shortages of MWs and AMWs; lack of resources (including birth kits and medicines), the need for better accountability and monitoring of AMWs (specifically, a need for AMW supervision, regulatory oversight and improved data collection for births attended by AMWs) and a need for health policy changes (particularly those governing AMW and MW role descriptions). Issues at the level of the healthcare provider were common, including the role and capacity of AMWs and MWs (particularly, AMWs capacity to take on additional tasks and the need for MWs to provide better supervision for AMWs); the need for improved quality, regulation and monitoring of AMW and MW education and training and the willingness, buy-in and motivation of AMWs and MWs, as well as the relationships between these health cadres. Factors at the level of the patient/community included the existence of strong community support for and trust in AMWs (described as an important facilitator). Cultural practices and health-seeking behaviours were also identified, specifically the perceptions that many women are reluctant to seek hospital care (due to financial and cultural factors) and AMWs were less likely to refer women to hospital.

Respondents ranked the feasibility of implementing AMW-specific task-shifting interventions in Myanmar (top ranked recommendations are listed in Table 1). Identified barriers to implementation of these prioritized recommendations included: variable capabilities of AMWs to perform tasks appropriately; poor drug quality, supply, and availability to AMWs; need for additional monitoring of drug use (including inappropriate use) and performance supervision; and insufficient funds, materials and skilled trainers for training. More task-specific barriers included current policies that do not permit AMWs to use these interventions; a lack of AMW-specific national guidelines (on prevention of PPH, neonatal resuscitation and management of puerperal sepsis); and a lack of resuscitation equipment.

The workshop participants proposed that the primary targets for implementation efforts should be AMWs working in rural areas, whereas MWs and AMWs working in urban areas and interested in improving skills were secondary targets. Potential implementation strategies included: policy briefs (to support development of evidence-based health policies that are practical and feasible); registration and regulation of AMWs through a new or existing national organization (such as a new AMW council, or the existing National Nursing and Midwifery Council); development of AMW guidelines on obstetric emergency management; and standardization and improvement of AMW training, tailored to specific needs of rural-based AMWs. Incentives for training (e.g. honoraria) were proposed to encourage participation. The need for improvements in drug distribution (including better logistics) was also identified.

### Uganda

A total of 16 people completed the pre-workshop survey; the majority of respondents were from Kampala and Mityana (37.5% and 25.0%, respectively). Prevention and treatment of PPH was identified by all respondents as the highest priority guideline for implementation in Uganda. Thirty four stakeholders participated in the two-day in-person workshop, with balanced representation from each stakeholder group, including 20 (58.9%) practicing healthcare providers.

Four FGDs were conducted to elicit perceptions around factors affecting implementation of the WHO guidelines in Uganda. One cross-cutting issue that was consistently highlighted as a barrier to health care provision in general (including guideline implementation) was lack of human resources. This included shortages of doctors and midwives, poor recruitment and retention rates (particularly in rural areas) and limitations on allowable numbers of health care workers that can be recruited at various health facility levels (irrespective of actual workload). These staffing pressures can limit effective implementation due to lack of time for routine care practices (such as labour monitoring and partograph completion).

Gaps in drug procurement, management and distribution processes as well as lack of essential equipment such as refrigerators for oxytocin storage, fetal scopes and partographs, were also described as barriers. It was noted that certain medicines (eg: magnesium sulphate) are not approved for use at lower levels of the health care system (such as health centres), which hinders their use, despite awareness of WHO recommendations. Concerns were also raised regarding safety and appropriate medicine use, including what cadres of healthcare providers are trained in their use. Examples cited included the growing practice of labour induction in outpatient settings, and anecdotal reports of combined use of oxytocin and misoprostol for labour augmentation, resulting in uterine rupture in some cases.

The need for better site-specific data on implementation of evidence-based practices was also highlighted. While quantitative data on selected outcomes are routinely collected and reported to the Ministry of Health, participants reported these are not fed back to facilities to support improvements in care. All FGDs described the need for improved accountability and monitoring of provider adherence to guidelines and professional standards. However, they also described that current government policies prohibit the implementation of some recommendations. Specifically, lack of consideration of available research evidence in policy decisions that govern how some medicines are distributed and used by various cadres was cited. Two focus groups discussed that the administration of misoprostol by community healthcare workers is currently not supported by policy in Uganda, despite studies in Uganda that have demonstrated safety and effectiveness of this approach (under the supervision midwives). Lack of up-to-date guidance on recommended practices was also highlighted, particularly around the use of misoprostol for prevention of PPH in health facility settings where drugs like oxytocin and ergometrine are also available. Recent improvements in misoprostol availability in facilities have not yet translated into their routine use; there have been instances of misoprostol expiring unused.

Factors affecting implementation were also identified at the provider level including: negative attitudes (for example around the use of the partograph); lack of buy-in from providers (particularly, their resistance to change); a lack of knowledge and skills (for example in administering magnesium sulphate and in interpreting the partograph); and the lack of opportunities for professional development for providers. The need for clear task shifting/sharing policies was highlighted, as the current role definitions of certain cadres were thought to hinder implementation. At the patient/community level, factors identified included traditional beliefs (eg: misconceptions around certain medical conditions, such as eclampsia); a lack of knowledge and awareness of certain health behaviours (such as a fear that mobility during labour can negatively affect outcomes); and the role of socioeconomic status in seeking and accessing healthcare in Uganda. The high value placed on companionship during labour in Uganda was cited as an important facilitator.

Respondents ranked three recommendations as the most feasible (Table 1). In the small group discussions, participants were asked to consider potential strategies to implement priority recommendations. Each group was assigned 1–2 guidelines to focus on during discussions; however, implementation strategies that were broadly applicable were also welcomed. Table 1 summarises the six implementation activities relevant to prioritized recommendations.

### Tanzania

A total of 15 stakeholders completed in the pre-workshop survey. Survey respondents represented six different regions across Tanzania with Dar es Salaam the most highly represented region (50%). The survey respondents varied in terms of role and the level of the healthcare system in which they were situated. The majority of respondents (n = 11) selected the prevention and treatment of PPH guideline as the highest priority in Tanzania.

A total of 32 stakeholders participated in the in-person workshop. FGDs explored priority recommendations, as well as factors influencing their implementation. At the healthcare system level, participants identified a lack of access to resources (including drugs, equipment, supplies and human resources) and challenges to referring patients to higher-level facilities safely and quickly. Other factors included poor continuity of antenatal and postnatal care for women who cannot afford or access services, the need for improved monitoring and evaluation of medicines distribution, and the need for improved policies that support implementation. At the provider level, identified factors included: healthcare providers’ existing beliefs and attitudes regarding practices, and the need for their buy-in as part of implementation efforts; the need for improved knowledge, skills and self-efficacy amongst providers; and the need for continuous training, coaching, and professional development to ensure skill retention and adherence to guidelines. At the community level, identified factors included: women’s health-seeking behaviors and preference for care; the role of community champions in promoting care-seeking in early labour, and financial constraints affecting women’s abilities to seek and use care.

In the ranking exercise, five recommendations were deemed the most feasible to implement in the Tanzanian context (Table 1). In small groups, participants considered potential implementation strategies—a summary of these strategies is shown in Table 1.

### Ethiopia

Activities in Ethiopia focused on WHO recommendations on prevention and treatment of postpartum haemorrhage. Fifty-three stakeholders completed the pre-workshop survey and nineteen stakeholders participated in the in-person workshop. The survey identified ‘use of uterotonics’ as the highest priority (66%) in clinical area within the guideline.

Findings from FGDs described issues at the level of the healthcare system, including: access to resources (a lack of drugs, supplies, personnel and facilities); challenges to drug procurement, distribution and management (including stock-outs, particularly in rural areas); data collection and monitoring (particularly, a lack of routine data collection and reporting on maternal deaths and a lack of facility-level audit and feedback to improve provider performance); the need for policies to mandate protocol adherence; need for incentives in facilities to allow more equitable distribution of human resources, and for more visible and accessible guidelines and protocols. Notably, while participants agreed readiness to change was important, there were mixed views regarding current readiness. At the Ministry of Health level, the level of readiness for health system changes related to guideline implementation was perceived to be high. However, at the provider level, some participants perceived resistance to change (i.e., lack of buy-in, low morale), while others disagreed, indicating that providers would recognize the importance of the issue and will support change regardless of frustrations related to low morale.

Several factors at the level of the healthcare provider that may affect guideline implementation were also identified. Some participants indicated that provider resistance to using available guidelines, lack of adherence, and lack of buy-in by providers can negatively affect use of guidelines. A lack of knowledge and skills was also identified. Participants described a lack of knowledge exchange opportunities between senior healthcare providers and newly trained providers, and a need for more training, supportive supervision and mentorship opportunities. Positive inter-professional dynamics and a clear understanding of one’s role were discussed as factors that may facilitate guideline implementation at the provider level. At the community level, factors identified included: preferences regarding childbirth location (home births in Ethiopia are common); a lack of knowledge and awareness amongst pregnant women about the risks of home birth (and the need for more and better education to address this) and the financial, economic and social barriers preventing women from accessing to healthcare services.

In the ranking exercise, five recommendations were deemed most feasible to implement in the Ethiopian context (see Table 1). Based on these findings, ten recommended activities were formulated by the group to guide next steps of guideline implementation in Ethiopia (see Table 1).

## Discussion

In this multi-country study, we used a systematic approach (based on the KTA model) to identifying barriers and facilitators to implementation of prioritized WHO maternal heath recommendations in four lower-income countries, and to identify implementation strategies. This activity has demonstrated that this approach is feasible and can guide implementation in resource-constrained settings. Effective KT is challenging—KT interventions targeting healthcare providers typically only produce 10% absolute change in behavioural outcomes [29], highlighting the need for research on how to optimize and facilitate implementation processes in a more standardized way. Simultaneously, contextual differences in LMIC settings emphasize the need for tailoring. The current study presents how we have operationalized the first few steps of a systematic process model (the Knowledge to Action cycle) to inform implementation activities designed to lead to behaviour change and improved patient outcomes.

WHO develops and disseminates evidence-based guidelines to support improvements in quality of care, however guidance on how these can be implemented is often lacking. For example, a recent content analysis of 123 WHO guidelines highlighted that active, evidence-based guidance for implementation is often lacking.[30] Understanding that guideline development and dissemination activities are not enough to ensure changes in practices and outcomes,[14,15,29] the action cycle of the KTA model is designed to guide implementation planning in a variety of contexts (see Fig 1). The first stage of the action cycle is to identify needs. All countries had existing needs (high rates of maternal and perinatal mortality), yet each country was able to prioritize guidelines most relevant to addressing their setting’s specific priorities (e.g. task shifting in Myanmar). By selecting recommendations based on key stakeholder priorities, results of the research are more applicable to their needs. Issues relating to guideline adaptation (the next step in the action cycle) were discussed during the FGDs. When guideline recommendations were not feasible in a country context (e.g. not having access to misoprostol, one of the recommended medicines for prevention and treatment of PPH), this was identified by participants and flagged for follow-up, so that the guideline recommendations could be adapted to the local context, for example using tools such as ADAPTE.[31]

The next step within the KTA cycle is to understand the barriers and facilitators to guideline implementation. The FGDs allowed participants to explore these at multiple levels, including woman/community, healthcare provider, and health system/policy levels. Systematic reviews and studies of barriers and facilitators to implementation of clinical guidelines have previously been conducted.[32] However, by engaging stakeholders directly in identifying barriers and facilitators and prioritizing guideline recommendations, they are better able to understand local challenges and opportunities for implementation, to select relevant and appropriate implementation strategies and tailor them to the local context. This approach also helps stakeholders link together the barriers and facilitators with implementation strategies to focus efforts on priority areas and issues. The selection of implementation strategies is driven by behaviour change theory and linked directly back to the identified barriers and facilitators.[14] For example, if providers lack the knowledge and skills (barriers) to implement the guidelines, there is an underlying capability issue (behaviour change theory), which may lead the group to select education (implementation strategy). Alternatively, if there are system-level challenges related to the scope of work (a barrier identified in the Myanmar activity on midwifery task-shifting), this describes an issue with opportunity (behavior change theory) and the implementation strategies selected may focus on policy changes. A 2015 Cochrane review by Baker and colleagues included cluster-randomized trials on the use of ‘tailored’ interventions (selection of interventions based on identified determinants of practice) to improve professional practice and healthcare outcomes, including guideline adherence.[33] They reported that tailored implementation appears to be generally effective, albeit of small to moderate magnitude. However, it remains to be seen whether this is also true in resource-limited settings: only four trials were conducted in middle-income countries (two trials each in South Africa and Indonesia) and none in low-income countries.

Throughout this activity, we used an integrated KT approach which emphasizes how end users and key stakeholders should be involved throughout the project and ideally take ownership of next steps.[19] While traditional knowledge translation focuses on conducting research then disseminating findings, integrated KT is a collaboration that focuses on “partnerships, respect, knowledge exchange, mutual learning and co-production of knowledge”. There is emerging evidence that integrated KT, specifically end-user engagement can increase academic impacts and more directly address relevant clinical problems.[34,35] Integrated KT aligns with the participatory research approaches emphasized when working in LMICs. There is evidence that participatory approaches can affect substantive maternal and perinatal adverse outcomes in LMIC settings. For example, a Cochrane review by Lassi and colleagues found that community participatory interventions (such as community mobilization, community support groups and women’s groups) probably reduce neonatal mortality and may reduce maternal mortality.[36]

### Key Findings

Despite the different prioritized recommendations and contexts, barriers identified across countries were often similar. Findings suggested that provider knowledge was not the primary barrier to guideline implementation; rather, they described that poor implementation was largely due to practical issues. Health system level factors, including health workforce shortages, and need for strengthened drug and equipment procurement, distribution and management systems, were consistently highlighted as limiting the capacity of providers to deliver high-quality care, despite their willingness to do so. All countries identified the need for health policies to support these implementation efforts, specifically the need to address outdated or restrictive health policies that were not in line with current evidence (for example, policies governing what tasks midwives are allowed to perform). However, the need to improve knowledge and skills of healthcare providers (through education, coaching, mentorship and other professional development activities) was consistently identified as an important determinant of implementation at the level of the health provider.

Community/individual level determinants of guideline implementation were also quite consistent across countries. More generic barriers to utilizing care (including geographical, economic and social barriers) can complicate timely use of effective interventions.[37] For example, the benefits of oxytocin for prevention and treatment of PPH are likely limited when women present in facilities a long time after childbirth. These factors highlight the need for guideline implementation efforts to explicitly consider and address factors affecting communities and individual women. The KT literature reflects a disproportionate focus on interventions directed at healthcare providers,[29] with comparatively less knowledge on the effectiveness of interventions directed at individual women and communities.

### Strengths and Limitations

The strengths of this study include the use of a mixed-methods approach that incorporates theory and evidence. It has permitted a standardized assessment of barriers and facilitators to inform implementation efforts, as well as offering a roadmap for future directions. This approach could be applied to other guideline implementation challenges. However, it has not yet been demonstrated that this approach is superior to other approaches in terms of its effect on clinical outcomes (such as morbidity and mortality); further evaluation is required.

Our study had several limitations. First, we had variable numbers of participants and responses to the pre-workshop survey, largely due to technological difficulties. As the survey was circulated to large groups of people to maximize number of responses, the denominator (and response rate) is unknown. As the survey was anonymized, we are unable to assess what proportion of survey responses came from workshop participants. While we regard the survey as a useful resource to inform the workshop discussions, with experience we came to consider the diversity of participants represented within the focus groups as a more important determinant of success. While we aimed to engage a mixed group of stakeholders, community representatives and advocates were under-represented. More effort on how to best engage them is important (for example, considering separate community-specific research activities).

Although these activities were conducted in multiple countries, the scope of the activity was deliberately narrowed to specific priorities for implementation within each country, with consideration of broader contextual or systems factors that can affect or impede implementation. The findings cannot necessarily be generalized to other LMICs (with their own contexts and challenges), or to other implementation activities. However, other countries and organizations could adopt and adapt a similar methodological approach to implementation of maternal health guidelines (or other evidence-based guidelines) in their own settings.[16] Certain barriers (such as the need for training, and improved drug and equipment procurement) are likely relevant to a range of implementation efforts.

### Lessons Learned

Several lessons were learned in executing these activities, which could be incorporated into adaptations of this methodology. In some countries, a large number of recommendations were initially considered. This requires additional time and effort and adds complexity to activities. Focused, sustained implementation efforts addressing few (rather than several) KT gaps are more likely to succeed in the longer term. Hence we advise narrowing the focus at the outset to as few recommendations as is practical.

Identifying and engaging local stakeholders as champions was critical—ideally, such stakeholders are well-resourced and motivated, have local networks and partnerships that can be leveraged to achieve project objectives, and can navigate local challenges. Such stakeholders should be engaged early and often, with a sincere emphasis on local leadership. Conversely, staff turnover of key individuals (such as within Ministries of Health) can endanger guideline implementation efforts—institutional ownership by the appropriate local entities (such as the Ministry of Health, WHO Country Office or university department) is therefore advised. Consideration should be given to (where relevant) aligning or combining guideline implementation efforts with existing local or national initiatives on improving quality of maternal healthcare. Effort should also be made to maintain ongoing engagement with workshop participants, such as sharing resources on coaching, technical support, funding or other opportunities.

In the FGDs, we noted a tendency to over-emphasize barriers; additional attention to considering facilitators is advised. We came to appreciate that the “right mix” of stakeholders in the workshops was essential. Including policymakers, researchers, clinicians and other stakeholders with sufficient seniority and power to make decisions was necessary to ensure buy-in for future implementation efforts. Representation from frontline clinicians (including doctors, midwives, and nurses) meant that the discussions focused on the practical reality of delivering care, which can often be missed in discussions by senior policymakers alone.

### Next Steps

In this paper we have reported on activities relating to the first few steps of the KTA cycle. The GREAT Network partners continue to support activities in these countries related to subsequent steps—findings from these will be the subject of a separate, future publication. Evaluation of how this approach can improve guideline implementation (in terms of both process and outcome indicators) will also be required. For example, we recently conducted a process evaluation of the outcomes in Kosovo two years after the workshop.

Guideline implementation efforts in lower-income countries need to be more consistently documented and reported, so that all can learn from successes and failures in the field. The local working groups, with support from the GREAT Network, are developing and implementing activities based on these findings. For example in Myanmar, findings of this activity stimulated revisions of Ministry of Health policies regarding the scope of auxiliary midwifery practice and training, including administration of misoprostol for PPH prevention (prior to referral), management of puerperal sepsis with oral antibiotics, and performance of neonatal resuscitation. The workshop outputs and related discussions played a major role in influencing the policy changes. However, successful implementation of the policies and strategies identified in these activities will require strong leadership at multiple levels, sustained engagement of multiple stakeholder groups and mobilization of necessary financial, technical and human resources to support them.

## Conclusions

In order to support guideline implementation, we used a systematic approach to identify barriers and facilitators to the implementation of maternal and perinatal health guidelines at the health system, provider and community level in four lower-income countries. It is a flexible, feasible approach that can inform and help to optimize implementation efforts based on local barriers and facilitators to guideline implementation.

## Supporting Information

S1 File Final report: findings from surveys, focus groups and OPTIMIZEMNH Guideline workshop in Yangon, Myanmar. (PDF)

S2 File Understanding Barriers and Facilitators to Implementation of Maternal Health Guidelines in Uganda: A GREAT Network Research Activity—Final report on findings. (PDF)

S3 File Understanding Barriers and Facilitators to Implementation of Maternal Health Guidelines in Tanzania: A GREAT Network Research Activity—Final report on findings. (PDF)

S4 File Understanding Barriers and Facilitators to Implementation of Maternal Health Guidelines in Ethiopia: A GREAT Network Research Activity—Final report on findings. (PDF)

## Abbreviations

AMWs: Auxiliary midwives
FGDs: focus group discussions
GREAT: Guideline-driven, Research priorities, Evidence synthesis, Application of evidence, and Transfer of knowledge
KT: Knowledge translation
KTA: Knowledge-to-action
LMICs: low- and middle-income countries
MOH: Ministry of Health
MWs: Midwives
NGO: Non-governmental organizations
PPH: Postpartum haemorrhage
RCT: Randomized controlled trial
RHR: Department of Reproductive Health and Research
UN: United Nations
UNDP: United Nations Development Programme
UNFPA: United Nations Population Fund
UNICEF: United Nations Children’s Fund
WHO: World Health Organization
