# Supplementary material for: Barriers, Facilitators and Priorities for Implementation of WHO Maternal and Perinatal Health Guidelines in Four Lower-Income Countries: A GREAT Network Research Activity
Source: PLoS One. 2016 Nov 2;11(11):e0160020. doi: 10.1371/journal.pone.0160020 (PMC5091885; doi:10.1371/journal.pone.0160020)
Supplement: S1 File — (PDF) [file pone.0160020.s001.pdf]

GREAT Project [Guideline-driven, Research priorities, Evidence synthesis, Application of evidence, and Transfer of knowledge]

Final Report: Findings from Surveys, Focus Groups, and OPTIMIZEMNH Guideline Workshop in Yangon, Myanmar

Prepared by:

Sobia Khan[1], Caitlyn Timmings[1], Joshua Vogel[2], Katherine Ba Thike[3], Julia Moore[1], Metin Gülmezoglu[2], Nhan Tran[2], Sharon Straus[1]

[1]Knowledge Translation Program, Li Ka Shing Knowledge Institute, St. Michael's Hospital, Canada

[2]World Health Organization

[3]Consultant, Myanmar

Prepared for:

GREAT Project Stakeholders, Myanmar

Acknowledgements

We would like to thank the Department of Health Myanmar for hosting us in Yangon, and would like to especially thank Dr. Theingi Myint for her help in organizing the project activities. We appreciate the assistance of Dr. Thwe Thwe Win (Burnet Institute), Dr. Ko Ko Zaw (Department of Medical Research, Lower Myanmar), Dr. Kyu Kyu Than (University of Melbourne), Dr. Maung Maung Lin (World Health Organization Country Office, Myanmar) and Ms. Nay Zar Oo (World Health Organization Country Office, Myanmar) throughout the duration of this project.

Contact

For questions about this report, please contact:

Caitlyn Timmings, Research Coordinator
Knowledge Translation Program
Email: timmingsc@smh.ca
Phone: 416-864-6060 ext. 77566

Abbreviations

**MNH**	Maternal and newborn health
**LMIC**	Low or middle income country
**KT**	Knowledge translation
**WHO**	World Health Organization
**GREAT**	Guideline-driven, Research priorities, Evidence synthesis, Application of evidence, and Transfer of knowledge
**AMW**	Auxiliary midwife
**MW**	Midwife
**TMO**	Township medical officer
**FG**	Focus group
**NGS**	Non-government stakeholders
**PHS**	Public health supervisor
**IUD**	Intrauterine device
**PPH**	Post-partum haemmorrhage
**IV**	Intravenous

Executive Summary

Background

Informed by joint consultations with the World Health Organization (WHO) and the Department of Health Myanmar, the 2012 WHO *OPTIMIZEMNH* guideline on task shifting in maternal and newborn health was selected as a priority for implementation in Myanmar. Objectives included: 1) identifying barriers and facilitators to the implementation of the task shifting guidelines in Myanmar; and 2) developing a cross-cutting, multi-level implementation strategy to improve the use of the guidelines. The purpose of this report is to provide in-country stakeholders in Myanmar with key findings from pre-workshop and workshop activities.

Methods

Multiple methods were used to collect data on priorities, barriers and facilitators, as well as to determine potential implementation strategies for the task shifting guideline in Myanmar. Primary data collection occurred during an in-country two-day workshop involving focus group (FG) discussions, a ranking exercise, and small and large group discussions to explore barriers and facilitators, identify guideline priority areas, and develop action-oriented and tailored implementation strategies. Prior to the workshop, a survey was administered to inform workshop proceedings.

Findings

The process of selecting priority maternal and newborn health (MNH) recommendations and exploring barriers and facilitators to implementing the task shifting guideline yielded rich information for implementation planning in Myanmar. Participants in all FGs discussed **issues at the level of the healthcare system**, which included various factors related to the widespread conditions of the Myanmar context that can affect the implementation of the task shifting guidelines. These factors include *shortage of midwives (MWs) and auxiliary midwives (AMWs)*, *available resources, accountability and monitoring*, and *policies and political context.* **Issues at the level of the healthcare** provider were prevalent, where several barriers related directly to the AMWs, and MWs, physicians and clinicians were described. Specific factors discussed by FG participants include *role and capacity of AMWs and MWs; education and quality of training; willingness, buy-in and motivation;* and *relationships between health cadres.* Finally, **issues at the level of the patient/community** were described and included *community/patient perceptions of AMW and MW roles*, and *cultural practices and health-seeking behaviours*.

The ranking exercise resulted in a list of task shifting recommendations that were deemed feasible to implement according to workshop participants. Within the small group discussions, several recommendations to overcome barriers were suggested as the building blocks for an action plan to guideline implementation.

Recommendations

The findings of the pre-workshop survey aligned with those of the in-person workshop; both data collection methods helped to shape concrete strategies for moving forward on selected guideline

recommendations. The most salient points that emerged from the pre-workshop and workshop activities were:

- Task shifting should be considered across the system, where tasks can be shifted vertically and laterally in a manner that is feasible and suitable to Myanmar's largely rural context.
- Proper training and education of multiple cadres of health care workers is essential to optimal implementation of the task shifting guidelines. Specifically, tailored training for AMWs should focus on:
    - Oral supplement distribution to pregnant women;
    - Administration of misoprostol to prevent postpartum haemorrhage (where auxiliary nurses are already an established cadre);
    - Administration of misoprostol to treat postpartum haemorrhage before referral (where auxiliary nurses are already an established cadre and where a well-functioning referral system is in place or can be put in place);
    - Management of puerperal sepsis with oral antibiotics; and
    - Performance of neonatal resuscitation (bag and mask).
- Overall, training can increase trust and buy-in across all levels, and can improve perceptions about the roles of midwives (MWs) and AMWs.
- Myanmar may consider reviewing and defining the AMW role in terms of how individuals are selected, trained, retained, regulated, and supervised in order to make positive and sustainable changes to how maternal care is delivered, especially in rural and remote settings.
- Guideline implementation requires policymaker buy-in and a push for changes at the policy level, including:
    - Engaging policymakers and professional organizations with evidence briefs;
    - Revising policies related to drug administration and distribution;
    - Financially committing to provision of drugs and equipment to AMWs; and
    - Instituting regulatory oversight of AMWs.

Many of the barriers and facilitators discussed regarding the guidelines are applicable to other priority areas; therefore, these findings can inform and be integrated into future barrier and facilitator assessments conducted for additional guideline implementation initiatives in Myanmar.

Table of Contents

Background ... 6
Partnership with Myanmar ... 6
Purpose of report ... 7
Methods ... 7
Participant recruitment ... 7
Pre-workshop survey ... 7
In-person workshop: Focus groups, individual ranking exercise, and small group discussions ... 8
Focus groups ... 8
Individual ranking exercise ... 8
Small group discussions ... 9
Analysis ... 9
Triangulation of methods ... 9
Findings ... 9
Pre-workshop surveys ... 10
In-person workshop ... 13
Focus groups ... 13
Individual ranking exercise and small group discussions ... 20
Limitations ... 24
Recommended Future Directions ... 24
Summary and Conclusions ... 25
References ... 27
Appendix A: Pre-workshop survey ... 28
Appendix B: Focus group discussion guide ... 33
Appendix C. Comparison of Auxiliary Midwife Role in Myanmar to WHO Definition ... 35

Background

All healthcare systems struggle to optimally use evidence, resulting in inefficiencies and reduced quantity and quality of life[1-6]. For example, while there is ample evidence supporting the use of life-saving commodities and prevention and treatment strategies for maternal and newborn health (MNH), high rates of maternal and infant mortality and morbidity are still prevalent, especially in low and middle-income countries (LMICs).Recognition of this challenge has created interest in how knowledge translation (KT) approaches can be tailored and applied in the area of MNH. This has, in turn, created a need to enhance capacity in KT to meet the demand worldwide.

The World Health Organization (WHO) has partnered with the Knowledge Translation Program at St. Michael's Hospital (SMH) in Toronto, Canada to establish an international partnership called the GREAT (Guideline-driven, Research priorities, Evidence synthesis, Application of evidence, and Transfer of knowledge) Network, funded by the Canadian Institutes of Health Research. Recognizing that LMICs struggle to implement evidence-based clinical practice guidelines that can reduce maternal morbidity and mortality, the GREAT Network uses a unique evidence-based KT approach to support LMICs in the implementation of such guidelines. Specifically, the GREAT Network brings together relevant stakeholders of the healthcare system to identify and assess the priorities, barriers, and facilitators to guideline implementation, and supports the efforts of stakeholders to develop a guideline implementation strategy tailored to the local context.

Partnership with Myanmar

This report focuses on the partnership between St. Michael's Hospital, WHO (Department of Reproductive Health and Research), and healthcare system stakeholders of Myanmar. Activities within this partnership were funded by the Implementation Research Platform of WHO. Informed by joint consultations with WHO and the Department of Health Myanmar, the 2012 WHO *OPTIMIZEMNH* guideline on task shifting[7] was selected as a priority for the in-country workshop and related activities in Myanmar. The task shifting guideline was selected to promote distribution of tasks and responsibilities among cadres of healthcare workers, with particular emphasis on the role of auxiliary midwives (AMWs), and to improve access to maternal and newborn care. This aligns with current Ministry of Health priorities to increase the number of AMWs in the country, with the objective to have one AMW per village, so as to improve coverage of basic MNH care to all areas of Myanmar with a particular focus on rural locales.

Objectives of this partnership include:

1. Identifying barriers and facilitators to the implementation of the task shifting guidelines in Myanmar;
2. Developing a cross-cutting, multi-level implementation strategy for improving the use of the guidelines;
3. Supporting local stakeholders in the delivery of activities as identified in the implementation strategy;
4. Supporting local stakeholders in the development of a monitoring and evaluation plan to capture both the process and intervention impacts; and

5. Providing ongoing resources and mentorship to local stakeholders on guideline implementation.

Purpose of report

The purpose of this report is to provide in-country stakeholders in Myanmar with key findings from activities conducted to meet objectives 1 and 2 outlined above. Priorities, barriers, and facilitators related to implementation of the task shifting guideline are assessed in the body of this report, and practical recommendations are provided to effectively implement the guidelines on task shifting in Myanmar.

Methods

Multiple methods were used to collect data on priorities, barriers and facilitators, as well as to determine potential implementation strategies for the task shifting guideline in Myanmar. A survey of relevant stakeholders in Myanmar was administered to inform development of a two-day, in-country workshop. The workshop included small and large group discussions and a ranking exercise. These methods are briefly outlined below.

Participant recruitment

Participants were identified in consultation with the WHO Country Office Myanmar and the Department of Health, Myanmar. Participant selection was guided by role and level in which the individual operated in the health care system. To ensure representation from across the healthcare system, individuals with roles as healthcare administrators (e.g., programme managers, directors), township medical officers (TMOs), policymakers, non-governmental organization staff, representative from UN agencies working on maternal and newborn issues in Myanmar (UNFPA, UNICEF, WHO), representatives from professional societies and councils (e.g. Myanmar Nursing Council, Myanmar Nurse and Midwife Association), frontline clinicians [e.g., physicians (including obstetricians, neonatologists, pediatricians), midwives (MWs), auxiliary midwives (AMWs)], researcher/academics, and students/trainees were identified. Individuals representing different levels of the healthcare system were also identified – those working at the central (e.g. government agencies and national organizations/bodies) and local (e.g. township and regional level healthcare) levels.

Pre-workshop survey

The pre-workshop survey was designed to assess the perceived importance of task shifting as a healthcare system priority as well as the perceived barriers and facilitators to guideline implementation in Myanmar. The findings were, in turn, used to inform workshop facilitators about stakeholder perceptions, and to guide workshop proceedings. Surveys were administered from May to June 2014 in either an online or paper format [Appendix A]. Paper surveys were administered using a face-to-face method with a project team member (Dr. Thwe Thwe Win and personnel of MCH Division, Department of Health, Myanmar).

Participants were given a copy of the task shifting guidelines along with the survey. The survey included sixteen common barriers to guideline implementation, informed by the Theoretical Domains Framework.[8] Respondents were asked to rate their level of agreement with each of the potential

barriers on a scale from 1 to 7 (where 1= strongly disagree and 7= strongly agree) to gain an understanding of the types of barriers that were seen to be important in the context of implementation of the task shifting guidelines in Myanmar. Respondents were able to expand on barriers using open-ended responses. To gain insight into the factors that may aid implementation of the task shifting guideline, respondents were also asked to identify potential facilitators in open-ended questions. Descriptive statistics (count, proportion, mean and standard deviation) were used to analyze categorical and ordinal data. Thematic content analysis was employed for open-ended survey questions.[9]

In-person workshop: Focus groups, individual ranking exercise, and small group discussions

A sample of survey respondents and additional participants who represented the stakeholder groups of interest (described above) were invited to participate in an in-person workshop. Email invitations were sent to potential participants by the Department of Health Myanmar in June 2014. In advance of the in-person workshop, confirmed participants were provided with a package containing a summary of the task shifting guideline and recommendations, the agenda, and an overview of the workshop goals. Written consent was obtained from participants upon arrival to the workshop using a standardized information letter and consent form.

On Day One of the workshop, presentations on KT/guideline implementation, the WHO guideline development process and the task shifting guideline were delivered. Workshop attendees also participated in FG discussions. On Day Two of the workshop, key points from the FGs were discussed, priority areas were determined using a ranking exercise, and implementation strategies were selected in small group discussions. Methods for the FGs, ranking exercise and small group discussions are outlined below.

Focus groups

To achieve methodological rigour and ensure saturation of themes, participants were divided into four FGs, each comprised of approximately five to 15 participants. FGs were organized according to role and/or level of the healthcare system: FG1= AMWs; FG2= MWs; FG3= Central level staff; and FG4= non-governmental organizations, UN agencies working on maternal and newborn health issues in Myanmar (UNFPA, UNICEF, WHO) and researchers (hereon referred to as the 'non-government stakeholders' or NGS group). Each FG was assigned one expert facilitator, one translator and one note taker. All facilitators used a standard semi-structured discussion guide [Appendix B] to ensure use of a systematic approach while also allowing for FG questions to be tailored to the target groups. FG sessions lasted approximately 90 minutes. The focus groups centred on a discussion of recommendations from the guidelines that should be prioritized for implementation, and key barriers and facilitators to implementing these recommendations.

Individual ranking exercise

A total of 11 guideline recommendations were included in the ranking exercise. Five of the 11 recommendations were those identified during FG sessions as key tasks that typically fall within the competencies of the auxiliary midwife (AMW) role in the global context, but were not currently being performed by AMWs in Myanmar. These recommendations were perceived to be valuable activities to

be taken up by AMWs in Myanmar and included in the ranking exercise. The other six recommendations included were those rated as "Strong Recommendations" in the task shifting guideline – i.e. those that have strong evidence or expert consensus supporting the implementation of that guideline.

Workshop facilitators engaged participants in a nominal group process[10] to rate the feasibility of implementing each of the 11 identified recommendations. Consistent with the RAND Appropriateness Method[11], participants individually ranked each recommendation, using a 9-point Likert scale (where 1= extremely not feasible and 9= extremely feasible). An electronic audience response system was used so that participant ratings could be shared in real time.

Small group discussions

Following the ranking exercise, small group breakout discussions were conducted by facilitators using the same four groupings as used on Day One for FG discussions. Each group was assigned one translator and one note taker. In the first part of the small group discussions, participants were guided in an exercise to map implementation barriers to four of the 11 priority recommendations: three of these recommendations were identified as most feasible during the ranking exercise, and one was ranked as the least feasible but was felt by senior administrators to be important to consider implementing because of the potential impact on maternal health. In the second part of the small group discussions, participants were asked to identify implementation strategies that could potentially address identified barriers.

Analysis

FG sessions were digitally recorded and transcribed verbatim by a trained transcriptionist. After familiarization of the data from the transcripts and notes, data were qualitatively analyzed in NVivo 10 software by two independent analysts at SMH using a thematic analysis approach. Themes were developed in consultation with meeting facilitators to discuss interpretations of the data for a shared understanding of key findings.

Results from the individual ranking exercise were analyzed using descriptive statistics (proportion) of assigned feasibility ratings for each of the 11 recommendations. Small group discussions were analyzed using the same method as described for FG sessions above.

Triangulation of methods

Using the technique of integration, data collected across all methodologies were considered in detail together to draw meaningful and pertinent recommendations that are feasible and relevant for the Myanmar context.

Findings

A total of 31 individuals participated in the pre-workshop survey, and 42 participated in the two-day workshop. A description of the participants is provided in Table 1.

Table 1. Demographic information of pre-workshop survey respondents and workshop participants

	Survey (N=31)		Workshop (N=42)	
**Region**	n(n=30)	%	n(n=42)	%
Yangon (including Yangon, South Dagon, Thanlyin)	16	53.33%	28	67.00%
Nay Pyi Taw/Mandalay	12	40.00%		
Nay Pyi Taw	2	6.67%	14	33.00%
**Role (categories are not mutually exclusive)**	n (n=29)	%	n (n=42)	%
Auxiliary Midwives	8	27.59%	6	14.29%
Lady health visitors (i.e. midwife supervisors)	4	13.79%	2	4.76%
Midwives	8	27.59%	4	9.52%
Department of Health /Directorate of Medical Services	2	6.90%	10	23.81%
Township Medical Officers/Regional Health Representatives	2	6.90%	6	14.29%
Senior associate/managers	2	6.90%		
Hospital Administrators	2	6.90%		
Students/trainees	1	3.45%	1	2.38%
NGOs and UN agencies working on maternal and newborn health in Myanmar (UNFPA, UNICEF, WHO)			6	14.29%
Department of Medical Research			4	9.52%
Professional schools/associations (e.g. Nursing Association)			3	7.14%

Pre-workshop surveys

Survey findings are presented below according to three main themes: (1) task shifting among healthcare workers as a priority; (2) perceived barriers to guideline implementation; and (3) perceived facilitators to guideline implementation.

Task shifting among healthcare workers as a priority

The majority of respondents (n=30, 97%) agreed that task shifting to AMWs is a priority in Myanmar. The most common reasons provided to support this priority area for guideline implementation included: (1) an overall shortage of healthcare workers in Myanmar; (2) uneven distribution of healthcare workers, specifically, the majority of the population resides in rural areas whereas most healthcare workers are located in urban areas; and (3) MWs are overworked, signaling a need to reallocate tasks from MWs to other cadres of healthcare workers such as AMWs and public health supervisors (note that additional public health supervisor positions were created this year to help reduce the public health functions of the MW) . Other responses included a need to improve the quality of maternal care through a more realistic distribution of roles and responsibilities across cadres of healthcare workers and a need to reduce maternal morbidity and mortality rates. Only one respondent disagreed with task shifting as a current priority in Myanmar, but did not indicate why.

Perceived barriers to guideline implementation

Using a scale from 1 to 7 (where 1= strongly disagree and 7= strongly agree), survey respondents identified the following as the three most prevalent barriers to implementing the task shifting guidelines in Myanmar: (1) need for training, retraining, and supervision of AMWs to implement the guideline; (2) need for clear policy on roles and responsibilities of the AMWs; and (3) patient preference to be seen by a more experienced health worker than an AMW. The complete set of respondent ratings for each barrier can be found in **Table 2** below.

Table 2. Average respondent ratings (mean, standard deviation) of level of agreement with statements on barriers to guideline implementation, where 1= strongly disagree and 7= strongly agree.

Barrier (n= 31)	Mean	SD
Need for training, retraining and supervision to implement guideline	6.13	1.18
Need for clear policy on roles and responsibilities	5.97	1.20
Patient preference to be seen by a more experienced health worker	5.42	1.50
Not aware of process of development of OPTIMIZEMNH guidelines	4.71	1.51
Time pressures	4.71	1.47
Lack of familiarity with how to apply them	4.58	1.63
Not aware of OPTIMIZEMNH guidelines	4.42	1.73
Concern(s) about lack of autonomy over my practice	4.03	1.96
Lack of awareness that guidelines are supported by evidence	4.00	1.79
Lack of resources (including tools, services or training) to implement the guideline	3.94	2.02
Lack of confidence in guideline development or the developers	3.74	0.93
Lack of applicability to patients or work situations	3.58	1.63
Lack of cost-efficiency	3.55	1.55
Inconsistent with other guidelines or tools that I use	3.16	1.63
Inconsistent with my work (i.e., my routines)	2.97	1.62
Lack of practicality (i.e., too rigid)	2.94	1.88

Overall, respondents tended to view the task shifting guidelines as being consistent with their work routines, feasible for implementation, and did not perceive cost to be a significant barrier.

Additional barriers

Survey respondents were given the opportunity to identify additional factors that could act as barriers to implementation of the task shifting guideline in Myanmar. One barrier

> *"[Implementation of the OPTIMIZEMNH guideline may lead to] shifting burden to the midwives who already cover all different areas of primary health care and they are overburdened"* (Survey respondent)

that was identified by multiple respondents pertains to the current workload of MWs and AMWs. Specifically, respondents shared that MWs and AMWs may become overburdened if additional tasks and responsibilities are allocated to them, as recommended in the task shifting guideline.

Another perceived barrier that was commonly noted was a lack of confidence in the abilities of AMWs held by some physicians as well as some members of the general public.

> *"Since health volunteers are not government staff, there may be low levels of trust and reliance. Since they are not officially assigned, some social support groups may have different opinions."* (Survey respondent)

Issues at the administrative/systems level were also identified including: weak monitoring and evaluation mechanisms for care provided by MWs and AMWs; lack of a long-term strategy for MW career path and coordination among levels of the healthcare system; lack of equipment; lack of financial support; and lack of time for staff to attend training sessions. Lack of support for AMWs was identified by some respondents to be a barrier to task shifting in some communities, in particular in remote areas of the country. Finally, concerns around the potential for misuse of the new responsibilities granted within the task shifting guideline were identified as a barrier or risk of implementation.

Perceived facilitators to guideline implementation

The main themes that emerged from the survey around potential facilitators to implementing the task shifting guideline included: an identified need among physician groups for enhanced capacity and training for MWs and AMWs; evidence that task shifting is an effective strategy in other settings; and political (e.g., identification of maternal health as a priority) and financial commitments from the Department of Health Myanmar (e.g., training stipend for AMWs, availability of refresher training opportunities). Respondents also indicated that the guideline aligns well with other current Department of Health priorities.

> *"The Ministry of Health starts processing for task shifting in midwives (e.g., more appointment of PHS II at all sub centers for disease control tasks). It is the right time for implementation of [the OPTIMIZEMNH] guideline."* (Survey respondent)

Another facilitator that emerged in the pre-workshop survey was the high degree of acceptability and support for trained volunteers working in rural communities:

> *"There are trained volunteers already. In Myanmar, majority is rural population, and they tend to rely on native/local staff. Therefore upgrading local staff/ volunteers has high acceptability from community"* (Survey respondent)

In-person workshop

Focus groups

During the FGs, participants discussed the feasibility of implementing the *OPTIMIZMNH* guidelines in Myanmar. Findings are described in terms of issues at the *healthcare system level, healthcare worker level,* and *patient/community level* (Figure 1).

Figure 1. Overview of focus group discussion findings

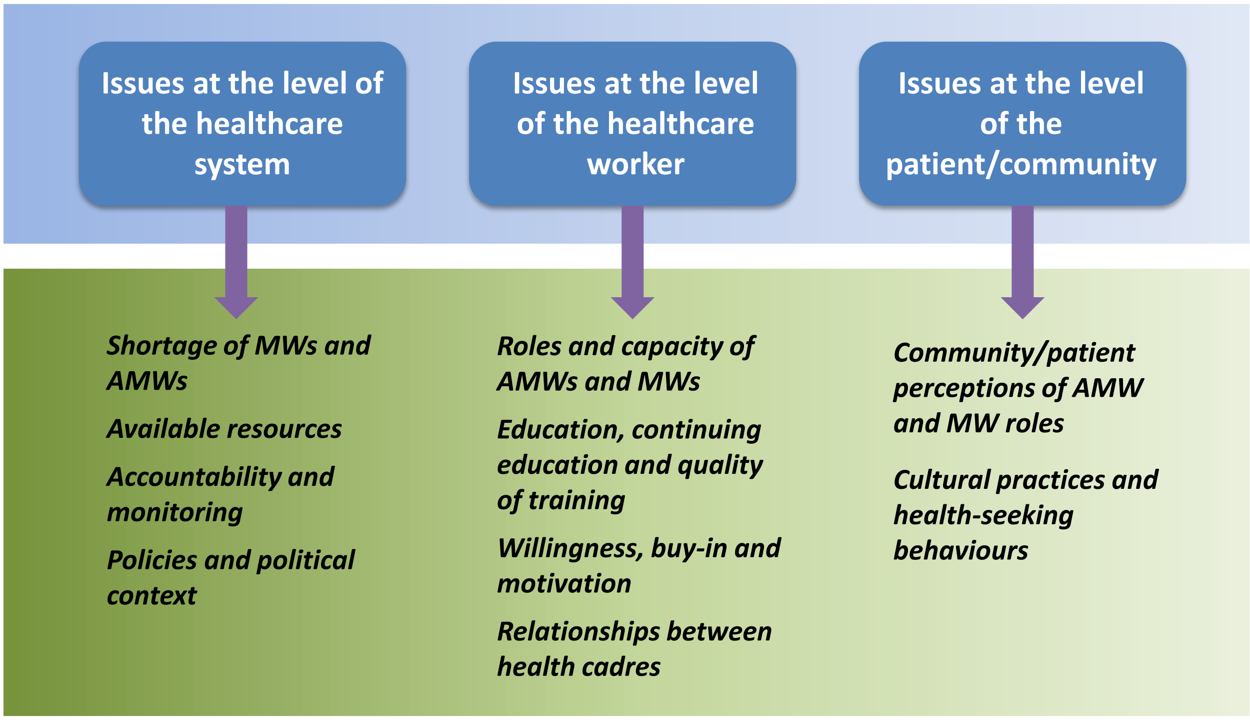

Issues at the level of the healthcare system

Participants in all FGs discussed various factors in the healthcare system and the widespread conditions of the Myanmar context that can affect the implementation of the task shifting guidelines. These factors include *shortage of MWs and AMWs*, *available resources, accountability and monitoring*, and *political climate.*

<u>Shortage of MWs and AMWs</u>

Participants in the Central and NGS FGs briefly discussed healthcare worker shortages in relation to task shifting, particularly the government initiative to produce more AMWs. While viewed as a necessary step, the current push to recruit AMWs was seen to have certain drawbacks. First, the Central level FG participants described that considering the shortage of MWs in Myanmar, there would be a larger number of AMWs operating without supervision. Second, most participants acknowledged that there is high attrition amongst AMWs given that they are not paid and often need to leave their positions to secure an income elsewhere. These factors were seen to threaten the implementation of task shifting.

To overcome the health care worker shortages and implement task shifting recommendations, Central level participants described the importance of recruiting more AMWs to work in remote areas by revisiting the AMW selection process and selecting for individuals who can be trained to take on higher level tasks. Participants for multiple groups described a need to provide incentives and motivate AMWs to continue to function and work in the AMW role, to reduce attrition.

Available resources

Participants were concerned about the capacity for AMWs to take on additional tasks given that they are unpaid and may not be provided with the equipment they require to perform certain healthcare functions. AMWs reported that they are using kits (including blood pressure cuffs, suction devices) that were provided to them at the start of their AMW service – some dating as far back as 1984 – and that replacement of equipment is often an out-of-pocket expense. Some AMWs may sporadically receive drugs from hospitals, whereas others routinely purchase drugs on their own for administration to patients. AMW participants reported spending anywhere from 3,000 to 7,000 kyats per month on medications alone. Money to purchase equipment and drugs is either secured through small service fees charged by AMWs to more socioeconomically stable families for delivery (about 5,000 kyats), or is borrowed from spouses or parents of AMWs.

Accountability and monitoring

Accountability and monitoring refers to the supervision mechanisms to ensure AMWs are implementing the relevant practices, should the task shifting guideline be implemented. It also describes access to data collection methods and infrastructure to determine outcomes of guideline implementation, as well as MNH outcomes overall.

Participants in all groups discussed the role of the MW in supervising AMWs and monitoring their performance. It was acknowledged that supervision of AMWs by MWs is not optimal due to the MWs' lack of time to monitor, physical distance from AMWs in some areas of the country (in a system where transportation costs are not covered), and potential lack of supervisory skills. Additionally, Central level, NGS and MW FGs were concerned about the lack of regulation of AMWs by a professional organization. If the task shifting guideline were to be implemented; participants felt that regulatory oversight would need to be introduced to ensure AMW accountability to best practices.

With regard to monitoring health outcomes, AMW participants described use of a 'birth book' or maternal-child health handbook as their means of collecting data regularly from mothers and babies; specifically, they captured information on maternal blood pressure at each visit and the baby's weight at delivery. AMWs give this information to the MW, and are unaware of what happens to the data thereafter. Central level participants reported that data collected from the AMWs by the MW is entered into the Health Management Information System (HMIS), but wondered whether a process should be implemented to collect data directly from AMWs. Both the Central level and NGS groups discussed that monitoring of MNH outcomes in Myanmar requires improvement, both in terms of *what* is collected (i.e. defining clear indicators for collection and reporting), as well as *how* it is collected. Moreover,

participants in all groups mentioned that there is little opportunity for AMWs to receive feedback on their performance, based on any of the data that are collected.

Policies and political context

Participants from all groups indicated that current government policies prohibit the implementation of some of the task shifting guideline recommendations. Specifically, current Myanmar health policies prevent AMWs from administering injections, except in exceptional situations (e.g. in remote townships where they are trained and supervised to do so). Similarly, some drug policies indicate that MWs can only administer the first dose of a medication (e.g., magnesium sulfate), and must refer patients to the hospital to receive additional doses. The NGS focus group agreed that the rules and regulations for all health worker cadres would need to be revised in order to support a system of task shifting (i.e. task shifting from physicians to MWs, and from MWs to PHS and AMWs); and to re-evaluate health worker competencies and accreditation based on changing guidelines.

NGS FG participants perceived that the presence of conflict areas in Myanmar may affect implementation of the task shifting guidelines in those areas; participants described Myanmar as a country “still in transition’, which may prevent nation-wide roll-out. For example, in remote areas where non-state actors are present, roll out of certain clinical practices has been impacted and their presence may influence implementation of the task shifting guidelines.

Issues at the level of the healthcare worker

Several barriers related directly to the AMWs, and MWs, physicians and clinical leaders were described. Specific factors discussed by FG participants include *role and capacity of AMWs and MWs; education, continuing education and quality of training; willingness, buy-in and motivation;* and *relationships between health cadres.*

Role and capacity of AMWs and MWs

Current roles and tasks of AMWs and MWs, as described by these two groups, are outlined briefly in **Table 3**. To help frame this discussion, Appendix C was developed to compare the AMW role as it currently stands in the Myanmar context to the role definition outlined by the WHO.

Table 3. AMW and MW roles and tasks

Current AMW tasks	MW Tasks related to the AMW role
• Health promotion and education with regard to ante- and post-natal care.<br>• Support for the MWs;<br>○ Collection of patient information for health services such as universal childhood immunization and post-natal follow up<br>○ Organization of women and children to receive health services from MWs<br>○ Accompany MWs on home visits to support them in service provision<br>○ Assist the MW to perform/conduct normal deliveries<br>○ Provision of information on contraception<br>• Disease control activities [gastroenteritis, dengue haemorrhagic fever, tuberculosis, and prevention of mother-to-child transmission of HIV] including:<br>○ Education (e.g., clean water storage to limit the spread of dengue haemorrhagic fever)<br>○ Follow-up for tuberculosis cases<br>○ Organizing communities to receive tetanus toxoid injection from the MW<br>• Administer some drugs to patients, including paracetamol, Burmeton (chlorphenamine maleate), oral salt solution, ergot, vitamin b1 and amoxicillin, if requested by MW to do so. *	• Some role overlap with AMWs (i.e. responsible for ante- and post-natal care, delivery, and disease control)<br>• Coordinate MNH and disease control services<br>• Supervise the AMWs<br>• Receive information from the AMWs for reporting purposes as well as to determine next steps in the patient's care pathway (e.g. treatment, referral to a tertiary care centre)<br>• Providing certain services/treatments (e.g. vaccinations, contraception)

*Note: Although technically prohibited from doing so, participants reported that AMWs are regularly administering drugs such as antibiotics to individuals.

Participants from all FGs raised concern about the capacity of AMWs to take on additional tasks. Non-AMW groups (i.e., MW, Central level and NGS groups) expressed concern about AMWs' capacity for learning the tasks outlined in the task shifting guideline. Participants from these groups were concerned about the capacity of AMWs to learn, internalize and apply the tasks given that most of these individuals have a low level of formal education and training in necessary skill areas. Additionally, participants pointed out that AMWs in Myanmar have less formal education and receive less skills training than outlined in the WHO's role definition, which impacts their ability to perform the tasks presented in the task shifting guideline. Currently, standard training lasts 6 months - 3 months of classroom teaching and 3 months of clinical/practical work.

When discussing the role and capacity of AMWs, participants also spoke about the MW role in Myanmar. One challenge described by the MW and the Central level groups was role conflicts between AMWs and MWs. They generally felt that MW and AMW roles were unclear despite existing role definitions at the national level. Leaders [i.e., Township Medical Officers (TMOs)] may prohibit MWs

from performing tasks that they were trained to do if they do not believe that the task is within the MWs competencies. This acts to diminish the MWs’ role and potentially contributes to an unclear demarcation between the MW and AMW role. MW participants expressed a desire to expand their role to include additional competencies, and believed that there should be more role recognition and distinct responsibilities from AMWs.

Discussion on the capacity of MWs pertained mostly to their capability of supervising AMWs. Central level participants perceived that many MWs may not have the skills to effectively supervise the work of the AMWs, and participants from multiple groups acknowledged that MWs are currently overburdened and may not have the time to supervise AMWs. Some participants were Lady Health Visitors whose role is to supervise MWs but who had assumed a MW role due to the heavy workload of MWs in their township, particularly because the MW role is split between public health functions and MNH. MW participants believed that shifting tasks to AMWs, in theory, should enable MWs to take on additional tasks, but that in reality, their workload would not decrease as their supervisory role would increase.

Education, continuing education and quality of training

All groups discussed the current state of education and required directions for health care worker training. AMWs and MWs felt their training would be enhanced with enhanced practical and hands-on components. Central level participants echoed this concern. Many drawbacks of the current training programs were identified, including large class sizes, short courses, and poor facilitation. As well, MWs felt that course participants were too diverse (i.e. representing too many different health worker cadres), and that not all participants were relevant learners for MNH issues [e.g. public health supervisors (PHS)]. These participants were viewed to be distractions to others for whom course work is essential. Central level and MW participants acknowledged that training quality varied by township, where some townships delivered better quality training than others.

The Central level FG participants noted that there was no regulatory body that monitors training and education quality for AMWs. All FG participants described improvements in training (i.e., providing ongoing, high quality training in small groups with more practical components, and ongoing monitoring of competencies and training quality through a central mechanism) as an essential component of successfully implementing the task shifting guidelines.

Willingness, Buy-in and Motivation

AMW participants reported that they were keen to learn more, to improve their skills and to take on additional tasks under the supervision of a MW or when a MW is not available (e.g., in rural locales). These participants indicated that they love their work and are motivated by their commitment to their community, many of whom they consider family. AMWs perceived becoming more skillful to be helpful in improving MNH outcomes in cases when a MW is unable to attend to a woman during pregnancy and delivery, and the woman and baby after delivery.

MW participants generally supported the task shifting guideline, and perceived that some (but not all) AMWs are truly interested in healthcare and are capable of taking on more responsibilities. They

believed that AMWs could be motivated to take on additional tasks by appealing to their interest in healthcare, but also by offering incentives such as training and collaboration with health staff, meals/snacks, equipment, medications, and health services for their own families. MW buy-in for the guideline was increased by the prospect of advancement in MW roles and competencies. Participants described the guideline as an opportunity for MWs to take on more complex tasks, as some of their current work will be shifted to the AMWs; however, it was acknowledged that having the time to take on additional work will depend on how quickly and seamlessly disease control tasks can be shifted laterally to the PHS.

The remaining groups had different opinions on the acceptance of the task shifting guideline. While the task shifting guideline was widely perceived to be a priority for Myanmar (as indicated by survey respondents as well), many participants in the Central level and NGS groups did not have confidence that policymakers and medical doctors would accept task shifting as routine practice. They discussed that the fear of consequences related to shifting tasks to lower-skilled cadres of health worker often outweighed the benefits of these actions. There was also a fear of misuse of some drugs (e.g. misoprostol) if AMWs were able to administer them widely in areas where there is little supervision by MWs or physicians.

Both the Central level and NGS FG participants doubted whether MWs were willing to shift their tasks to AMWs, and believed that MWs would need to be provided with some incentive to accept task shifting (e.g., an expansion of the MW role and greater supervisory responsibilities). A few participants also questioned AMWs' motivation for wanting to take on additional tasks; these participants believed that most health workers are willing to expand their repertoire of services as it will be an opportunity for them to charge patients for these services and therefore increase or generate income.

One participant noted that before considering the implementation of task shifting guidelines, consultation and buy-in must be secured from groups such as the professional societies representing pediatricians and obstetricians for elements of maternal-child health care.

<u>Relationships between health cadres</u>

A concern among FG participants across all groups with regard to task shifting was its potential effect on relationships between health worker cadres. Of particular importance was the AMW and MW relationship; AMWs, although keen to take on more responsibility, acknowledged that they did not want to rival MWs by assuming more tasks. As such, AMWs felt more comfortable with task shifting in cases when a MW is not available. AMWs and MWs indicated that in some areas of the country, the two health worker cadres were very close and worked well alongside each other, whereas in other areas, there may be more discord and an unwillingness to work together. MWs described this conflict to either be personal (i.e. personality conflicts) or financial (i.e. competition for service fees) in nature. MW participants indicated that some MWs are less willing to work directly with AMWs because of a perceived hierarchy of roles. The level of trust gained by the community can also cause conflict between MWs and AMWs; MW FG participants reported that AMWs who are backed by community members may disregard the MW and work more autonomously.

All FGs described relationships among AMWs, MWs and other health worker cadres as key factors in the implementation of the guidelines. Hospital staff were often perceived as deterrents for AMWs and MWs to follow guidelines. For example, some participants reported that AMWs and MWs were often scolded and blamed for the patient's condition when accompanying a patient with an obstetric emergency to the hospital. They believed that this may lead to conflicting behavior across the cadres; specifically they perceived that it may make AMWs less likely to refer patients to a hospital, and MWs more likely to lose confidence in their own training and refer more quickly to a hospital rather than administering a relevant intervention. MW FG participants indicated that hospital staff often do not understand AMW and MW capacities and skill sets, which can be a point of contention when referring a patient to the hospital.

Most FGs described that adherence to guidelines depends on what township leaders allow the MWs and AMWs to do based on their level of trust in these health worker cadres; one example offered by three groups was that MWs in some townships were not allowed to conduct IUD insertions despite this being within their current training and competencies. Thus, leadership attitudes and trust in their health care workers were seen as challenges to guideline implementation, but also major facilitators if leaders improve the confidence and skills of health care staff.

Finally, the NGS FG participants mentioned that ethnic and language differences in some areas may affect the relationships among health worker cadres at all levels.

Issues at the level of the patient/community

Participants briefly discussed barriers and facilitators to task shifting at the level of the patient and community. These are described below in terms of *community/patient perceptions of AMW and MW roles*, and *cultural practices and health-seeking behaviours*.

Community/patient perceptions of AMW and MW roles

Community and patient perceptions of the AMW and MW roles were considered important factors in guideline implementation. As described above, AMWs who have worked for many years in their communities have gained the community's trust. In some cases, this motivated AMWs to provide optimal care, whereas other times AMWs were perceived by MW and Central level participants to be entrenched in their ways with no accountability for changing their practices since they have strong support from their communities. In these latter cases, MWs, who are lesser known to the community, will have little influence.

Cultural practices and health-seeking behaviours

Participants described a cultural practice in some places in Myanmar of avoiding admission to a hospital unless the clinical situation is extremely dire. Because of this, it was perceived that AMWs are preferred to MWs in some communities as they are less likely to refer to a hospital. Participants also stated that the nature of the AMW job increases their role within communities and enables the AMW to work more autonomously – specifically, as most AMWs are selected from their community and have more

interaction and contact with community members, the communities often have more faith in the AMW. Central level participants described that most patients cannot afford to give MWs some remuneration so are reluctant to seek their help. As well, AMWs follow up with women after the delivery of their child, whereas participants reported that MWs rarely provide that level of continuous care because of their other responsibilities.

Individual ranking exercise and small group discussions

When asked to rank the feasibility of implementing tasks not currently practiced by AMWs as well as the strong recommendations of the task shifting guideline, workshop participants selected the following tasks and recommendations as the top 5 most feasible to implement in Myanmar (all results are presented in **Table 4**):

- Oral supplement distribution to pregnant women;
- Administration of misoprostol to prevent postpartum haemorrhage (where auxiliary nurses are already an established cadre);
- Administration of misoprostol to treat postpartum haemorrhage before referral (where auxiliary nurses are already an established cadre and where a well-functioning referral system is in place or can be put in place);
- Management of puerperal sepsis with oral antibiotics;
- Performance of neonatal resuscitation (bag and mask); and
- Initiation and maintenance of injectable contraceptives using a standard syringe.

Tasks ranked as most feasible were described by workshop participants to be the easiest to perform. Those ranked as least feasible were tasks that were described as complicated, introduced safety concerns, and were in direct opposition with current Myanmar policy regarding drug administration.

Table 4. Ranking of tasks not included in the task shifting guideline recommendations but identified by FG participants as activities currently not performed by AMWs; and strong recommendations for task shifting. Results in each grouping indicate *proportion of respondents (%)*, and are listed in order from most to least feasible, where 1 = extremely unfeasible and 9 = extremely feasible.

		Score (%)								
		Extremely unfeasible								Extremely feasible
	**Recommendation**	**1**	**2**	**3**	**4**	**5**	**6**	**7**	**8**	**9**
Tasks not included in OPTIMIZEMNH guideline but identified by FG participants as activities currently not performed by AMWs	Oral supplement distribution to pregnant women	0	0	0	0	0	0	10	17	76
	Administration of misoprostol to prevent and/or treat PPH	0	0	0	3	0	3	28	17	48
	Management of puerperal sepsis with oral antibiotics	8	4	4	4	0	4	12	44	20
	Low-dose aspirin distribution for women at high risk of eclampsia or preeclampsia	26	11	0	0	7	7	15	30	4
	Administration of oxytocin to prevent and/or treat PPH using a standard syringe or a compact pre-filled auto-disable device	38	21	7	3	0	14	17	0	0
	Management of puerperal sepsis with intramuscular antibiotics using a standard syringe	46	27	8	4	0	4	8	0	4
OPTIMIZEMNH strong recommendations	Performance of neonatal resuscitation	4	0	0	0	4	8	12	36	36
	Initiation and maintenance of injectable contraceptives using a standard syringe	11	7	7	0	4	15	19	15	22
	Suturing of minor perineal/genital lacerations	18	11	4	7	7	25	11	14	4
	Internal bimanual uterine compression for PPH	46	12	4	12	8	8	12	0	0
	Administration of IV fluid as part of treatment for PPH	62	15	8	0	8	4	0	0	4
	Insertion and removal of IUDs	64	11	7	0	4	4	0	4	7

Of the top five most feasible recommendations and tasks selected by workshop participants, the three selected for further discussion in small groups were: 1) administration of misoprostol to prevent and/or treat PPH; 2) management of puerperal sepsis with oral antibiotics, and; 3) performance of neonatal resuscitation.

Workshop facilitators also asked small groups to consider implementation strategies for a fourth recommendation ranked by the majority of participants as extremely unfeasible, but considered by the WHO to be a life-saving commodity for women with PPH, particularly in rural or remote settings: administration of IV fluid as part of treatment for PPH. A summary of the specific barriers identified for each of these four recommendations/tasks (note: these barriers were discussed in greater detail in FG discussions and described above) and potential implementation strategies as identified by workshop participants are presented in **Table 5**.

Table 5. Selected recommendations/tasks, barriers to implementation and potential implementation strategies that can alleviate barriers, as identified by workshop participants.

<table>
<tr><th></th><th>Barriers unique to the task</th><th>Barriers common across most tasks</th><th>Implementation Strategies</th></tr>
<tr><td>Administration of misoprostol to prevent and/or treat PPH</td><td>Current policies prevent AMW administration of misoprostol</td><td rowspan="4">Education level and variable capabilities of AMWs to perform task appropriately

Drug quality, supply, and availability to AMWs **

Monitoring use/misuse and supervision of performance

Insufficient budget and lack of materials and skilled trainers for training</td><td rowspan="4">Target population: Primary targets for task shifting would be AMWs working in rural areas; secondary targets would be AMWs working in urban areas with demonstrated capacity for learning/interest in learning.

Advocacy to policy makers: Develop policy briefs outlining evidence for reversing restrictive policies (e.g., injections, administration of oral antibiotics).

Registration and regulation: To ensure monitoring and performance evaluation, consider registration and regulation of AMWs by a national organization. Suggestions included regulation through: the Nursing Association; the Ministry of Health; or, an AMW council (which would have to be developed).

Development of a guideline for management of emergency obstetric cases by AMWs: To include recommendations on all four tasks discussed.

Education: Standardized training at an organizational level for AMWs (e.g., through a professional regulatory body); intensive, practice-oriented courses; ongoing refresher training. Consider tailoring training to the needs of MWs and AMWs, particularly to those in rural areas who will need a higher level of training because of limited access to other health care providers. Skilled trainers and aids for practical training opportunities need to be secured. Training and competencies of AMWs should be regularly evaluated. Incentives for training can be considered (e.g., honorarium) to ensure attendance.

Drug distribution: Policies that inhibit drug administration are in conflict with the current availability of drugs (i.e., most drugs are widely available in drugstores). Systems-level controls of drug availability and quality were suggested. AMWs drug use can be regulated by provision of drugs through the system; AMWs can be provided with good quality medications through MWs, who receive their drugs through the TMOs.</td></tr>
<tr><td>Management of puerperal sepsis with oral antibiotics</td><td>Current policies prevent AMW administration of antibiotics

No current national guideline on management of puerperal sepsis by AMWs</td></tr>
<tr><td>Performance of neonatal resuscitation</td><td>Lack of equipment: bag and mask, bulb mucous extractor

Approval from Paediatric Society required

No current guideline on neonatal resuscitation</td></tr>
<tr><td>Administration of IV fluid as part of treatment for PPH*</td><td>Current policies prevent AMWs from administering IV fluid</td></tr>
</table>

*participants still disagreed with this recommendation after discussion in small groups. It was suggested that alternatives to IV fluid administration could include provision of anti-shock garment or oral salt solution as a volume expander.

** note: misoprostol and antibiotics are available for purchase over the counter

Limitations

There are three main limitations to data collection to declare. First, data were collected from a small sample that may not be representative of the entire population working in the MNH sector of Myanmar; however, the sample was highly diverse and included individuals from government and professional associations, as well as all categories of health workers from represented townships. It should be noted that no patients were included in any of the data collection. As well, the pre-workshop survey did not have optimal participation, and data were lost from clinicians, physicians (OB/GYN), the Myanmar Nursing Council and the Myanmar Nurse and Midwife Association. Second, time, resource, and space restrictions were faced by project organizers in conducting this activity; therefore, a purposeful convenience sample was used to identify stakeholders to participate in the pre-workshop survey and two-day workshop. As a result, most stakeholders were recruited from two large regions. Finally, language and cultural barriers may have prevented a more robust understanding of the data, but translation was used during and after the workshop to enhance comprehension of the data.

Recommended Future Directions

Multiple recommendations emerged from the pre-workshop and workshop findings that require action at the policy, research, and practice levels. The recommended action areas are presented below in **Figure 2,** with distinction among levels of action (i.e., policy, research, practice) and timelines for action (i.e., shorter and longer-term goals).

Figure 2. Recommendations for future directions in Myanmar, stratified by shorter- and longer-term goals for policy, research and practice areas.

	Policy	Research	Practice
**Shorter-term Goals**	***Engage policymakers with evidence and examples of task shifting in other countries to increase buy-in.***<br><br>***Engage professional organizations e.g. paediatric society, nursing council.***	***Engage researchers to develop an implementation process/ outcomes evaluation strategy.***<br><br>***Review indicators in the reproductive health strategy for monitoring and evaluation that are feasible for the Myanmar context.***	***Revise role definitions by: revisiting MW and AMW competencies in Myanmar and comparing these with competencies of similar cadres globally.***<br><br>***Disseminate role definitions widely to all level of health care workers.***
**Longer-term Goals**	***Review mechanisms for antibiotic distribution and administration by AMW, under supervision of MW.***<br><br>***Ensure that AMWs have appropriate equipment to perform tasks.***<br><br>***Incorporate regulatory oversight of AMWs in the mandate of a selected organization.***	***Collect baseline data on practices and attitudes of health care workers toward MNH practices.***<br><br>***Plan and conduct studies related to the process and outcomes of guideline implementation.***	***Change training curricula and models to be competency-based and tailoring them to meet the needs of health workers.***<br><br>***Plan and execute ongoing performance evaluation for AMWs using established indicators for quality of care (e.g following procedures according to guidelines).***

Summary and Conclusions

The process of selecting priority MNH recommendations, and exploring barriers and facilitators to implementation of the task shifting guideline, has yielded rich information and considerations for implementation planning in Myanmar. The findings of the pre-workshop survey aligned closely with those of the in-person workshop; both data collection methods helped to inform concrete strategies for moving forward in introducing selected guideline recommendations to the local context. The most salient points that emerged across the pre-workshop and workshop activities were:

- Task shifting should be considered across the system, where tasks can be shifted vertically and laterally in a manner that is feasible and suitable to Myanmar's largely rural context.
- Competency-based training and education of multiple cadres of health care workers is essential to optimal implementation of the task shifting guidelines. Specifically, tailored training for AMWs should focus on:
    - Oral supplement distribution to pregnant women;
    - Administration of misoprostol to prevent and/or treat PPH;
    - Management of puerperal sepsis with oral antibiotics;

    - Essential care of newborns including neonatal resuscitation (bag and mask);
- Overall, training, teamwork and provision of constructive supervision and feedback to MWs and AMWs can increase trust and buy-in across all levels, and can improve perceptions about the roles of MWs and AMWs.
- Myanmar may consider re-evaluating the AMW role in terms of how individuals are selected, trained, assigned tasks, retained, regulated, and supervised in order to make positive and sustainable changes to how maternal care is delivered, especially in rural and remote settings.
- Guideline implementation requires policymaker buy-in and a push for changes at the policy level, including:
    - Engaging policymakers and professional organizations with evidence briefs
    - Reviewing mechanisms for distribution related to administration of antibiotics and other drugs for specific conditions
    - Ensuring MWs and AMWs have the requisite equipment to perform their responsibilities properly
    - Instituting regulatory oversight of AMWs

The methods used to inform the implementation strategies discussed in this report are transferable to other priority areas and other guidelines, particularly those in the area of maternal-child health. Moreover, many of the barriers and facilitators discussed regarding the task shifting guidelines are applicable to other priority areas; therefore, these findings can provide a platform for such discussions and be integrated into barrier and facilitator assessments conducted as part of additional guideline implementation initiatives in Myanmar.

To move forward, we recommend creating a working group for planning task shifting guideline implementation activities consisting of key stakeholders within Myanmar, including (but not limited to): Ministry of Health staff, representatives of professional organizations and regulatory bodies (e.g. nursing association, neonatology society), township medical officers, physicians, MWs, AMWs, and researchers. Implementation support in terms of training and capacity building can be provided by the GREAT Network throughout the process to aid in-country stakeholders in achieving implementation goals.

References

1. McGlynn EA, *et al*.: **The quality of health care delivered to adults in the US**. *N Engl J Med* 2003, 348:2635-2645.

2. Davis D, Evans M, Jadad A, Perrier L, Rath D, Ryan D, Sibbald G, *et al*.: **The case for KT: shortening the journey from evidence to effect.** *BMJ* 2003, **327**(7405)**:**33-35.

3. Madon T, Hofman KJ, Kupfer L, Glass RI: **Public health. Implementation science.** *Science* 2007, **318**(5857)**:**1728-1729.

4. Shah BR, Mamdani M, Jaakkimainen L, Hux JE. Risk modification for diabetic patients. Are other risk factors treated as diligently as glycemia? Can J Clin Pharmacol 2004; 11(2):e239- e244.

5. Pimlott NJ, Hux JE, Wilson LM, Kahan M, Li C, Rosser WW. Educating physicians to reduce benzodiazepine use by elderly patients: a randomized controlled trial. CMAJ 2003; 168(7):835-839.

6. Kennedy J, Quan H, Ghali WA, Feasby TE. Variations in rates of appropriate and inappropriate carotid endarterectomy for stroke prevention in 4 Canadian provinces. CMAJ 2004; 171(5):455- 459.

7. World Health Organization. **WHO recommendations: optimizing health worker roles to improve access to key maternal and newborn health interventions through task shifting 2012**. http://www.optimizemnh.org/ Accessed 6 Aug 2014.

8. Cane J, O'Connor D, Michie S: **Validation of the theoretical domains framework for use in behaviour change and implementation research**. *Implementation science : IS* 2012, **7**:3

9. Braun V, Clarke V. **Using thematic analysis in psychology**. Qual Res in Psychol 2006; 3 (2): 77-101.

10. Jones J, Hunter D: **Consensus methods for medical and health services research.** *BMJ* 1995, **311:**376-380.

11. Fitch K, *et al*.: **The RAND/UCLA appropriateness method user's manual**. RAND Corporation; 2001.

Appendix A: Pre-workshop survey

GREAT Project Assessment Survey- Myanmar

Introduction

Welcome to the GREAT Project (Guideline-driven, Research priorities, Evidence synthesis, Application of evidence, and Transfer of knowledge). The purpose of the project is to improve the quality of care for mothers and infants in Myanmar, to build capacity locally, and to develop a framework to implement the World Health Organization (WHO) Guidelines on Task Shifting (entitled "OPTIMIZE4MNH: Optimizing health worker roles for maternal and newborn health") at the local level.You are being invited to participate in a short survey to help the project team better understand the key priorities related to the WHO guideline on task shifting and the perceived barriers and facilitators to its implementation in the Myanmar context. Participation in the survey will take approximately 15- 20 minutes of your time. The responses of the survey are anonymous and will be used to inform the proceedings of a two-day in-person meeting to be held in Yangon (Myanmar) at a later date in June. By completing and submitting this survey, your consent to participate is implied. If you have any questions about the survey, please contact one of the following individuals:Dr. Katherine Ba Thike at kbathike@gmail.com or by phone: 94211 47174Dr. Thwe Thwe Win at thwetwin@gmail.comDr. Theingi Myint at theingimyint5@gmail.com or by phone: 95153862Dr. Ko Ko Zaw at zawkzaw@gmail.com or by phone: 95134 907Thank you very much for your time and participation.

Section I: Demographic Information

1. In which region/state or township do you work? Please respond in the box provided below.

Region/State: ☐

Township: ☐

2. At what level of the health care system do you work? Please check all responses that apply.

Central ☐

Region/State ☐

Township ☐

Village ☐

3. What is your title/role description?

4. How long have you been in this role?

○ Less than 1 year

○ 1-2 years

○ 3-5 years

○ 6-10 years

○ 11-20 years

○ More than 20 years

5. What are the five main tasks you carry out in descending order, starting from the most common. Please respond in the box provided below.

1.

2.

3.

4.

5.

Section II: WHO Task Shifting (OPTIMIZE4MNH) Guideline

For the purpose of this survey, "task shifting" is defined in accordance with the WHO guideline:

> "The term task shifting is used to describe a situation or those strategies and activities used to train and enable 'mid-level' and 'lay' health workers to perform specific interventions that might otherwise be provided only by cadres with longer (and sometimes more specialized) training.In Myanmar, we are considering task sharing/shifting from doctors to midwives and from midwives (MW) to auxiliary midwives (AMW)."

6. In your opinion, is task shifting among health care workers a priority for maternal and newborn health in Myanmar at this time?

○ Yes

○ No

7. If yes, what do you thing has led to the identification of task shifting as a priority?

[ ]

8. If no, why do you think task shifting is not a priority at this time?

[ ]

9. Please rate your agreement of the extent to which the following factors act as BARRIERS to the use of the WHO Task Shifting (OPTIMIZE4MNH) guidelines in your setting?

	1- Strongly Disagree	2- Disagree	3- Somewhat disagree	4- Neither agree nor disagree	5- Somewhat agree	6- Agree	7- Strongly Agree
Not **aware** of Optimize4MNH guidelines	○	○	○	○	○	○	○
Not **aware** of process of development of Optimize4MNH guidelines	○	○	○	○	○	○	○
Lack of **familiarity** with how to apply them	○	○	○	○	○	○	○
Lack of **awareness** that they are supported by evidence	○	○	○	○	○	○	○
Lack of **confidence** in their development or the developers	○	○	○	○	○	○	○
Lack of **applicability** to patients or work situations	○	○	○	○	○	○	○

Lack of **cost-efficiency**	○	○	○	○	○	○	○
Lack of **practicality** (i.e. too rigid)	○	○	○	○	○	○	○
	○	○	○	○	○	○	○
	○	○	○	○	○	○	○

The following statements apply to task shifting/sharing from doctors to midwives and from midwives to auxiliary midwives

	1- Strongly disagree	2- Disagree	3- Somewhat disagree	4- Neither agree nor disagree	5- Somewhat agree	6- Agree	7- Strongly Agree
Patient preference to be seen by a more experienced health worker	○	○	○	○	○	○	○
Inconsistent with my work (i.e. my routines)	○	○	○	○	○	○	○
Inconsistent with other guidelines or tools that I use	○	○	○	○	○	○	○
Time pressures	○	○	○	○	○	○	○
Concern(s) about lack of autonomy over my practice	○	○	○	○	○	○	○
Lack of resources (including tools, services or training) to implement the guideline	○	○	○	○	○	○	○
Need for clear policy on roles and responsibilities	○	○	○	○	○	○	○
Need for training, retraining and	○	○	○	○	○	○	○

supervision to
implement guideline

○ ○ ○ ○ ○ ○ ○

10. Are there any other barriers that you think could challenge the implementation of these guidelines in Myanmar? If so, please list in the space provided below, and give specific examples if possible.

11. Are there any facilitators that you think could aid in the implementation of these guidelines in Myanmar? Please list in the space provided below, and give specific examples if possible.

12. Are there any other stakeholders (for example members of the public, community leaders, health care managers, health care professionals, health care workers, policymakers, or key community members) that might be willing to participate in this survey?

Name 1:

Title/Role 1:

Email Address 1:

Mailing Address 1:

13. Is there anything you would like to add?

Appendix B: Focus group discussion guide

<table>
<tr><td colspan="2">Instructions for facilitators:

Welcome and introductions
Collect signed consent forms
Review process for guideline development and review WHO guidelines</td></tr>
<tr><td colspan="2">Questions for participants</td></tr>
<tr><td>1.</td><td>In your opinion, is task shifting among health care workers a priority for maternal and newborn health in Myanmar at this time?
If yes, what do you think has led to the identification of task shifting as a priority?
If no, why do you think it is not a priority at this time?</td></tr>
<tr><td>2.</td><td>Having reviewed the summaries of the WHO guidelines on OPTIMIZE4MNH: (Optimizing health worker roles for maternal and newborn health) presented in the Information Package, what do you think are some of the key messages relevant to Myanmar?
Prompt: Please provide rationale.</td></tr>
<tr><td>3.</td><td>Do you feel confident in your knowledge to implement the WHO guidelines? In your skills? In your ability?
Prompt: Please elaborate/expand on answer.</td></tr>
<tr><td>4.</td><td>How would you describe the attitudes (culture) of the staff towards the implementation of new guidelines in your health care setting?</td></tr>
<tr><td>5.</td><td>What do you think are the potential barriers or challenges to implementing these guidelines in Myanmar?
Probe: Do you feel that your health care setting has the capacity to incorporate these guidelines?
Probe: Do you feel that your health care setting has established sufficient organizational readiness to support this change?
Probe: What do you feel are some of the logistical factors that need to be considered?</td></tr>
</table>

	○ Probe: Are there additional barriers or challenges that you think may affect the implementation of these guidelines?
6.	What strategies would you suggest to overcome some of these potential barriers and challenges?
7.	What do you think are the potential facilitators that could aid in the implementation of these guidelines in your health care setting?<br>○ Probe for specific examples:<br>○ Physician champion? Other clinical champions?<br>○ Strong leadership?<br>○ Incentives and/or motivation for using guidelines?<br>○ Training?<br>○ Education/training?<br>○ Improved access to guideline materials?
8.	How do leaders within your organization/institution reinforce or reward improvements in process?
9.	How do you monitor/measure implementation efforts in your health care setting?
10.	Do you have any additional suggestions that could help your health care setting in implementing the WHO guidelines on task shifting?
11.	Before we wrap up today's discussion, is there anything else that anyone would like to add?
**Thank participants and wrap up**	

Appendix C. Comparison of Auxiliary Midwife Role in Myanmar to WHO Definition

The table below outlines the current capacity and role of auxiliary midwives in Myanmar as they align with the capacity and role of auxiliary midwives as defined by the World Health Organization.

WHO Definition of Auxiliary Nurse Midwife Role[1-3]	Auxiliary Midwives in Myanmar
Have some training in secondary school. A period of on-the-job training may be included, and sometimes formalized in apprenticeships. Like an auxiliary nurse, an auxiliary nurse midwife has basic nursing skills and no training in nursing decision –making. Auxiliary nurse midwives assist in the provision of maternal and newborn health care, particularly during childbirth but also in the prenatal and postpartum periods. They possess some of the competencies of midwifery but are not fully qualified as midwives. The recommendations also noted that definitions, length of training and competencies may vary between health systems.	Auxiliary midwives in Myanmar have some training in secondary school. They complete a 3 month on-the-job training programme. Auxiliary midwives assist in the provision of maternal and newborn health care, particularly during childbirth but also in prenatal and postpartum care. They generally work under the supervision of midwives but (particularly in rural settings) they may have no direct supervision.

For the purposes of comparison, the interventions defined by the WHO task-shifting recommendations as being within the auxiliary nurse midwife competency are listed below, as compared to those within the current auxiliary midwife competency in Myanmar.

Assumed competencies of Auxiliary Nurse Midwives within WHO recommendations	Current Auxiliary Midwife Competency in Myanmar
Promotion of maternal, newborn and reproductive health interventions	✓
Oxytocin administration to prevent PPH – standard syringe	
Oxytocin administration to treat PPH – standard syringe	
Oxytocin administration to prevent PPH – CPAD	
Oxytocin administration to treat PPH – CPAD	
Misoprostol administration to prevent PPH	
Misoprostol administration to treat PPH	
Oral supplement distribution to pregnant women	
Low dose aspirin distribution to pregnant women at high risk of pre-eclampsia/eclampsia	
Continuous support for women during labour, in the presence of a skilled birth attendant	✓
Puerperal sepsis management with oral antibiotics	
Puerperal sepsis management with intramuscular antibiotics – CPAD	
Maternal intrapartum care (including labour monitoring, e.g. using a partograph; foetal heart rate monitoring by auscultation; decision to transfer for poor progress; delivery of the baby)	✓

*PPH = post-partum haemorrhage; CPAD = compact pre-filled auto-disable device

[1] WHO recommendations: Optimizing health worker roles to improve access to key maternal and newborn health interventions through task shifting. Geneva, World Health Organization 2012.
[2]UNFPA. State of the World's Midwifery. New York: United National Population Fund. 2011.
[3]World Health Organization. Optimizing the delivery of key interventions to attain MDGs 4 and 5: background document for the first expert 'scoping' meeting to develop WHO recommendations to optimize health workers' roles to improve maternal and newborn health.(WHO 2010).
