# Supplementary material for: Barriers, Facilitators and Priorities for Implementation of WHO Maternal and Perinatal Health Guidelines in Four Lower-Income Countries: A GREAT Network Research Activity
Source: PLoS One. 2016 Nov 2;11(11):e0160020. doi: 10.1371/journal.pone.0160020 (PMC5091885; doi:10.1371/journal.pone.0160020)
Supplement: S2 File — (PDF) [file pone.0160020.s002.pdf]

Understanding Barriers and Facilitators to Implementation of Maternal Health Guidelines in Uganda: A GREAT Network Research Activity

---

Final report on findings

Entebbe, Uganda

21 and 22 August 2014

Prepared by:

Caitlyn Timmings[1], *Sobia Khan*[1], *Dr. Joshua Vogel*[2], *Dr. Lisa Puchalski Ritchie*[3], *Dr. Kidza Mugerwa*[4], *Dr. Denise Njama-Meya*[5], *Shusmita Islam*[1], *Dr. Julia E. Moore*[1], *Dr. A. Metin Gülmezoglu*[2], and Dr. *Sharon E. Straus*[1,3]

[1]Knowledge Translation Program, Li Ka Shing Knowledge Institute, St. Michael's Hospital, Canada

[2] UNDP/UNFPA/UNICEF/WHO/World Bank Special Programme of Research, Development and Research Training in Human Reproduction (HRP), Department of Reproductive Health and Research, World Health Organization, Headquarters, Switzerland

[3]University of Toronto, Canada

[4]Makerere University, Uganda

[5]PATH, Uganda

Table of Contents

Acknowledgements 2

Contact 4

Abbreviations 5

Executive Summary 6

Background 10

- Partnership with Uganda 10
- Purpose of report 11

Methods 12

- Participant recruitment 12
- Pre-workshop survey 12
- In-person workshop 12
  - Focus groups, individual ranking exercise, and small group discussions 12
  - Analysis 13
- Triangulation of methods 13

Findings 14

- Pre-workshop survey 14
  - Demographics 14
  - Prioritizing guidelines 14
  - Prioritizing recommendations within guidelines 15
- In-person workshop 16
  - Demographics 16
  - Focus groups 17
  - Individual ranking exercise 27
  - Small group discussions 27

Limitations 30

Recommendations to inform a country-specific implementation plan 31

Summary and conclusions 32

References 34

Appendix A: Uganda's healthcare system structure 36

Appendix B: Pre-workshop survey 37

Appendix C: Focus group discussion guides 45

Appendix D: Demographic information of pre-workshop survey respondents 49

Appendix E: Pre-workshop survey findings for priority recommendations for prevention and treatment of PPH guideline 50

Appendix F: Pre-workshop survey findings for priority recommendations for prevention and treatment of pre-eclampsia and eclampsia guideline 51

Appendix G: Pre-workshop survey findings for priority recommendations for augmentation of labour guideline 52

Appendix H: Pre-workshop survey findings for priority recommendations for induction of labour guideline....53

Appendix I: Median score and interquartile range (IQR) for feasibility rankings by guideline recommendation54

Acknowledgements

We would like to thank the Ministry of Health Uganda, Makerere University, World Health Organization (WHO) Country Office Uganda, and PATH for graciously hosting us in Entebbe, and would like to especially thank Drs. Collins Tusingwire, Kidza Mugerwa, and Denise Njama-Meya for their guidance and support throughout the process. We would like to acknowledge WHO, PATH and the United Nations (UN) Commission on Life Saving Commodities for funding the project activities. We also wish to acknowledge the GREAT Network for their strategic guidance in designing this activity. This activity is a part of a series of projects that the GREAT Network is involved with in partnership with low and middle income countries.

Contact

For questions about this report, please contact:

Caitlyn Timmings, Research Coordinator
Knowledge Translation Program
Li Ka Shing Knowledge Institute
St. Michael's Hospital
Toronto, Canada
Email: timmingsc@smh.ca
Phone: 416-864-6060 ext. 77566

Abbreviations

**FG**	Focus group
**GREAT**	Guideline-driven, Research priorities, Evidence synthesis, Application of evidence, and Transfer of knowledge
**IP**	Interprofessional
**IV**	Intravenous
**KT**	Knowledge translation
**LMIC**	Low and middle income country
**MDG**	Millennium Development Goal
**MMR**	Maternal mortality rate
**MNH**	Maternal and newborn health
**MW**	Midwife
**PPH**	Post-partum haemorrhage
**SMH**	St. Michael's Hospital
**UN**	United Nations
**WHO**	World Health Organization

Executive Summary

Background

This report is prepared for the Ministry of Health Uganda, stakeholders who participated in the in-person workshop activity hosted in Entebbe, Uganda on 21 and 22 August 2014, as well as all relevant healthcare system stakeholders in Uganda.

This activity is part of a series of guideline implementation activities supported by the GREAT (Guideline-driven, Research priorities, Evidence synthesis, Application of evidence, and Transfer of knowledge) Network. The GREAT Network was established in 2012 by the World Health Organization (WHO), St. Michael's Hospital (SMH)/University of Toronto, and international partnerships with low and middle income countries (LMICs) to provide guidance and technical support to LMIC stakeholders who are focused on enhancing maternal and perinatal health through the implementation of evidence-based WHO guidelines. The knowledge translation (KT) approach applied by the GREAT Network to optimize the uptake of guidelines in practice was piloted in 2012 in partnership with healthcare system stakeholders of Kosovo. Additional information on the study methodology and findings is published elsewhere[1].

Informed by global priorities set by the UN Commission on Life Saving Commodities on maternal health commodities and consultations with the WHO, Ministry of Health Uganda, Makerere University, and PATH the following four WHO guidelines on maternal and perinatal health were identified as key priorities for implementation planning activities in Uganda:

- Prevention and treatment of post-partum haemorrhage (PPH) (2012);
- Prevention and treatment of pre-eclampsia and eclampsia (2011);
- Induction of labour (2012); and
- Augmentation of labour (2014).

Objectives of this study included: 1) identifying barriers and facilitators to the implementation of the four priority guidelines in Uganda; 2) identifying the most important and feasible WHO recommendations for implementation; and (3) providing key recommendations to inform the development of a multi-level implementation strategy for improving local use of the guidelines. The purpose of this report is to share key findings from pre-workshop and workshop activities. These findings will subsequently inform the development, implementation, and evaluation of a strategy for improvement of use of guidelines.

Methods

Multiple methods were used to collect data on priorities, barriers, facilitators, and potential implementation strategies for the four identified guidelines in Uganda. Primary data collection occurred during an in-country two-day workshop involving focus group (FG) discussions, a ranking exercise, and small and large group discussions to explore barriers and facilitators; identify WHO recommendations to be prioritized; and develop potential implementation strategies to fit the local context. Prior to the workshop, a survey was administered to inform workshop proceedings.

Findings

Sixteen stakeholders participated in the pre-workshop survey and 34 stakeholders participated in the in-person workshop. Stakeholders represented multiple disciplines invited from diverse geographic regions and levels of the healthcare system including: healthcare administrators, policymakers, non-governmental organization staff, representatives from professional associations, frontline healthcare providers [e.g., physicians (including obstetricians and pediatricians) and midwives (MWs)], and researchers/academics.

Findings from FG discussions identified and explored facilitators and barriers to guideline implementation in the Ugandan context at multiple levels:

Factors at the level of the healthcare system included: *access to medication and equipment*; challenges in *drug procurement, distribution, and management; human resources; access to site-specific clinical data; accountability and monitoring of guideline implementation; and policies*. Key barriers and facilitators identified at the healthcare system level included:

Barriers:

- Lack of resources in healthcare facilities such as insufficient supplies of drugs (e.g., magnesium sulfate) and/or equipment/supplies (e.g., fetoscope, refrigerators).
- Difficulties with calculations required to reconstitute magnesium sulfate.
- Challenges with correct dosing of vaginal misoprostol.
- Gaps between registered medical indications of drugs and recommended uses in practice.
- Inefficiencies in drug procurement processes.
- Lack of facility capacity due to shortages of healthcare providers (e.g., physicians and midwives), especially in rural areas.
- Challenges with recruitment of healthcare workers (especially in rural areas).
- Fiscal constraints at the national level contributing to lack of healthcare providers in facilities.
- Data not routinely fed back from Ministry of Health to the healthcare facilities to allow for site-specific analysis and/or continual practice improvement.
- Lack of proper documentation in medical notes.
- Lack of accountability/monitoring of healthcare provider adherence to guidelines and professional standards.
- Lack of effective translation of research evidence into policy or practice (e.g., administration of misoprostol by community healthcare workers is currently not supported by policy in Uganda).

Facilitators

- Availability of oxytocin formulation that can be stored at room temperature.
- Ability to dilute oral misoprostol for lower-dose regimens (i.e., diluting the tablet in 200 mL of water).
- Access to site-specific data on some indicators to encourage practice change.
- Availability of delivery books in all healthcare facilities.

Issues at the level of the healthcare provider were prevalent, and included: *beliefs, attitudes, and buy-in about the guideline recommendations; knowledge and skills needed to implement the guidelines; training, coaching, and professional development around guideline implementation; and scope of roles.* Key barriers and facilitators identified at the provider level included:

Barriers

- Negative attitudes/beliefs about use of partograph (e.g., that it is inefficient or ineffective).
- Resistance to behaviour change to adhere to guideline recommendations.
- Anecdotal evidence or provider preferences for medical practices/drugs that are not recommended in guidelines.
- Lack of knowledge and skills (e.g., challenges with administering magnesium sulfate and properly formulating IV solution or use/interpreting partograph).
- No designated clinical instructors on the wards for some cadres of healthcare providers (e.g., MWs).
- Insufficient diversity of training modes (e.g., access to simulation centres, emergency preparedness drills).
- Lack of emphasis on knowledge exchange activities (i.e., those who do attend in-service trainings do not routinely share their learnings with colleagues in their healthcare settings).
- Current role definitions of various cadres of healthcare workers hinder the effective implementation of guideline recommendations in some cases.

Facilitators:

- Opportunities for reviewing healthcare cadre role definitions to facilitate task-shifting.
- Specialized trainings currently being offered to MWs in the area of balloon tamponades in some settings.

Issues at the level of the patient/community included: *traditional beliefs and perceptions of healthcare services; knowledge and awareness; and socioeconomic status.* Key barriers and facilitators identified at the patient/community level included:

Barriers:

- Traditional beliefs about the causes of eclampsia.
- Lack of awareness of benefits of some guideline recommendations (e.g., fear that mobilization will negatively affect labour) or harms of improper use of certain drugs.
- Lack of financial resources (e.g., to pay for transportation).

Facilitators:

- Positive cultural belief about the value of companionship.

The anonymous individual ranking exercise resulted in a participant-driven assessment of the feasibility of each of the 16 guideline recommendations that were deemed to be priorities in Uganda. Within the small group discussions that followed the ranking exercise, a variety of strategies were suggested to overcome barriers, serving as the building blocks for a guideline implementation plan.

Recommendations and conclusion

Potential strategies that could improve maternal health guideline utilization/implementation in Uganda were provided by healthcare system stakeholders representing multiple cadres. Key messages that emerged from the pre-workshop and workshop activities are as follows:

- There is a need and an opportunity to improve implementation of the priority recommendations across the four selected WHO guidelines.
- Drug procurement, management, and distribution practices are not operating at an optimal level. Examples were provided of drugs expiring on the shelves before they are used and of insufficient drug supplies. This suggests a need for a national and facility level review of current drug procurement and monitoring policies/practices.
- There is concern around the potential misuse of misoprostol. Further research is required to better understand how misoprostol can be safely used in the community, to understand the extent and types of misoprostol misuse currently going on and how to improve use of misoprostol in health facilities in Uganda. Results of this research could, in turn, support changes to policy.
- Recruitment of more healthcare workers (physicians and MWs) is needed across the healthcare system, but particularly in rural/remote areas; infrastructure (e.g., housing for healthcare workers, schools for the children of healthcare workers, etc.) and incentives (i.e., a competitive salary comparable to urban centres) are needed as a preliminary step. Eliminating the current recruitment ban on the hiring of physicians and MWs in Uganda may also be considered as an opportunity to increase access to human resources and ultimately frontline capacity to implement guideline recommendations.
- There is a need to create more formal linkages between healthcare facilities and village health teams to better coordinate and standardize maternal healthcare for the community.
- Patients and the wider community (e.g., family members and caregivers) would benefit from increased awareness about the harms and benefits of certain guideline recommendations (e.g., benefits of a companion during labour; medical causes of eclampsia). This could be achieved through strategies and activities directed at patients and the wider community (e.g., radio/SMS campaigns, birth plans, educational materials, community talks/meetings).
- Increased opportunities for training is essential to improving the implementation of guideline recommendations including: onsite orientation for new staff; use of simulation training/simulation centres; training at the bedside; improved supervision; use of clinical instructors; and development of coaching programs between more experienced healthcare workers and new professionals.

Many of the barriers, facilitators, and resultant implementation strategies identified regarding the four WHO maternal and perinatal guidelines are applicable to other priority areas in healthcare; therefore, these findings can inform and be integrated into future barrier and facilitator assessments and guideline implementation planning initiatives in Uganda.

Background

Despite a growing body of evidence to support clinical practice, including evidence-based guidelines, healthcare systems are failing to use this information optimally to improve the delivery of care[2]. Inadequate use of evidence in practice often results in inefficiencies, and reduced quantity and quality of life[2-7]. Low and middle-income countries (LMICs) face additional challenges to applying research evidence including: weak health system infrastructure; lack of professional regulation and opportunities for professional development and training; and lack of access to health data[8]. For example, while there is strong evidence supporting the use of life-saving commodities in prevention and treatment strategies for maternal and newborn health (MNH), there is limited availability and use of life-saving commodities in many LMIC settings which is contributing to high rates of maternal and infant mortality and morbidity. Current trends in maternal mortality rates (MMRs) suggest that most countries will not achieve the fifth Millennium Development Goal (MDG) target of a 75% reduction in MMR from 1990 to 2015[9]. Recognition of this challenge has created interest in how knowledge translation (KT) approaches can be tailored and applied to the area of MNH. This has, in turn, created a need to enhance capacity in KT to meet the demand worldwide.

The World Health Organization (WHO) has identified poor implementation of maternal and perinatal health guidelines globally as an important research priority in improving quality of care for mothers and newborns. In order to address this, WHO has partnered with the KT Program based at St. Michael's Hospital (SMH)/University of Toronto in Toronto, Canada to establish an international partnership called the GREAT (Guideline-driven, Research priorities, Evidence synthesis, Application of evidence, and Transfer of knowledge) Network, funded by the Canadian Institutes of Health Research. The GREAT Network uses a unique evidence-based KT approach to support LMICs in the implementation of guidelines as LMICs often experience challenges in routinely implementing evidence-based clinical practice guidelines that can reduce maternal morbidity and mortality. Specifically, the GREAT Network brings together relevant healthcare stakeholders in LMICs to identify and assess the priorities, barriers, and facilitators to guideline implementation, and supports the efforts of stakeholders to develop a guideline implementation strategy tailored to the local context.

Partnership with Uganda

This report focuses on the partnership established between the KT Program at SMH, WHO (Headquarters, Department of Reproductive Health and Research), PATH, and healthcare system stakeholders of Uganda. The activities of this study were funded by WHO, PATH and the UN Commission on Life-Saving Commodities. Ethical clearance to carry out study activities was sought and obtained from research ethics boards at Makerere University, SMH, and WHO.

Guideline selection was based on a multi-step process. The UN Commission on Life-Saving Commodities' identified oxytocin, misoprostol, magnesium sulfate as priority global maternal health commodities due to their importance in preventing and managing major causes of maternal and perinatal morbidity and mortality, including postpartum haemorrhage (PPH), pre-eclampsia, and eclampsia. Based on these, study partners mapped the three maternal health commodities against WHO's existing evidence-based clinical guidelines on maternal and perinatal health and developed a shortlist of candidate priority guidelines. Through consultation with the Ministry of Health Uganda on national priorities for guideline implementation, the following four guidelines were selected for the in-country workshop and related implementation activities in Uganda:

- Prevention and treatment of post-partum haemorrhage (PPH) (2012)[10];
- Prevention and Treatment of pre-eclampsia and eclampsia (2011)[11];
- Induction of labour (2012)[12]; and
- Augmentation of labour (2014)[13].

The objectives of the study include:

1. Identifying barriers and facilitators to the implementation of the four priority guidelines in Uganda;
2. Identifying the most important and feasible WHO recommendations for improvement of implementation;
3. Providing key recommendations to inform the development of a multi-level implementation strategy for improving local use of the guidelines;
4. Supporting local stakeholders in the development and delivery of the implementation strategy; and
5. Supporting local stakeholders in the development of a monitoring and evaluation plan to assess impact.

Purpose of report

The purpose of this report is to provide Ministry of Health Uganda, stakeholders who participated in the in-person workshop activity, and all relevant healthcare system stakeholders with key findings from activities conducted to date to meet objectives 1 - 3 as outlined above. Priorities, barriers, and facilitators related to implementation of the four WHO maternal health guidelines selected are assessed in the body of this report, and practical recommendations are provided to inform the effective implementation of the guidelines of interest in Uganda.

Methods

Multiple methods were used to collect data on priorities, barriers and facilitators, as well as to determine locally-relevant implementation strategies for the four selected guidelines. A survey of identified stakeholders in Uganda was administered to inform development of a two-day, in-country workshop. The workshop included small and large group discussions and a ranking exercise. These methods are briefly outlined below.

Participant recruitment

Participants were identified in consultation with the Ministry of Health Uganda, WHO, Makerere University, and PATH. To ensure representation from across the healthcare system, individuals with roles as healthcare administrators (e.g., district health office), policymakers, non-governmental organization staff, representatives from professional associations (e.g., Uganda Private Midwives Association, Uganda Pediatrics Association), frontline healthcare providers [e.g., physicians (including obstetricians and pediatricians) and midwives (MWs)], and researcher/academics were identified. Individuals representing different levels of the healthcare system were also identified to ensure representation from health centres II-IV, district/general hospitals, as well as regional and national referral hospitals [**Appendix A**]. Finally, geography was a key consideration in participant selection to ensure representation of stakeholders from both rural and urban centres across the country.

Pre-workshop survey

The pre-workshop survey was designed to provide a preliminary understanding of key priorities related to the identified WHO maternal guidelines in the Ugandan context. Surveys were administered in August 2014. The survey [**Appendix B**] was electronically disseminated to a wide variety of stakeholders. Participants received an email inviting them to participate in the survey and were asked about their preference for survey completion (web-based platform or paper-based survey). Consent was implied by completion of the survey.

In-person workshop

A sample of survey respondents and additional participants who represented the stakeholder groups of interest (described above) were invited to participate in a two-day in-person workshop in Entebbe, Uganda.

On **Day One** of the workshop, key background presentations were delivered. Workshop attendees then participated in focus group (FG) discussions, a semi-structured group interview process used to collect data on a specific topic. In the context of this activity, the objective of the FG discussions was to identify barriers and facilitators to implementation of priority guidelines. On **Day Two** of the workshop, key points from the FGs were shared (e.g., common barriers/facilitators) as well as the shortlist of priority recommendations (n=16) that were used for the ranking exercise. Small group discussions, action-oriented sessions to brainstorm recommendations for moving activities forward, were held to identify potential implementation strategies for priority recommendations.

Focus groups, individual ranking exercise, and small group discussions

Participants were divided into three FGs, each composed of approximately 8 to 15 participants. FGs were organized according to role and/or level of the healthcare system: FG1= physicians (including obstetricians and pediatricians); FG2= MWs; FG3= policymakers, researchers/academics, non-governmental organizations, and international organizations (hereon referred to as the 'interprofessional group' or IP group). Facilitators used

semi-structured discussion guides [**Appendix C**]. FG sessions lasted approximately 90 minutes and were conducted in English. The FG discussions centered on identifying priority recommendations based on importance and barriers and facilitators to implementing these recommendations in the Ugandan context.

A shortlist of recommendations was generated based on selections made in the **Day One** FG discussions and following deliberations among facilitators and local experts. On **Day Two**, workshop facilitators engaged participants in a nominal group process[14] to rate the feasibility of implementing each of the identified guideline recommendations. Consistent with the RAND Appropriateness Method[15], participants individually ranked each recommendation, using a 9-point Likert scale (where 1= extremely not feasible and 9= extremely feasible). In accordance with the RAND Appropriateness Method, a subset of the select recommendations was re-ranked due to disparate responses among participants.

Following the ranking exercise, small group breakout discussions were conducted by facilitators using the same three groupings as used on **Day One** for FG discussions. Participants were guided in an exercise to map implementation barriers to the priority recommendations, followed by an exercise to identify implementation strategies that could address identified barriers and that were locally relevant.

Analysis

Descriptive statistics were used to analyze categorical and ordinal survey data. For Section 2 (Guideline prioritization), count was used to depict the ranking of the four guidelines. For Section 3 (Prioritization of recommendations), data were recoded so that the highest ranking received the highest score (e.g., 1st ranked priority= assigned score of 4). The Total Score was then calculated to account for both the assigned rank and the number of times the guideline or recommendation was identified as one of the top five priorities.

FG sessions and small group discussions were digitally recorded and detailed notes were taken to supplement recordings. After familiarization of the data from the recordings and notes, data were qualitatively analyzed by an expert analyst at SMH using a thematic analysis approach.[16] Themes were developed in consultation with meeting facilitators to discuss interpretations of the data for a shared understanding of key findings.

Results from the individual ranking exercise were analyzed using descriptive statistics [median, interquartile range (IQR) including the score for the 25th percentile and 75th percentile] of participant assigned feasibility ratings for each of the identified recommendations. Small group discussions were analyzed using the same method as described for FG sessions above.

Triangulation of methods

Using the technique of integration, data collected across all methodologies were considered in detail together to draw meaningful and pertinent recommendations that are feasible and relevant for the Ugandan context.

Findings

Pre-workshop survey

Survey findings are presented below according to responses based on two main questions: (1) priorities between guidelines; and (2) priorities within guidelines (i.e., priority recommendations). Demographic information of the survey respondents is also provided.

Demographics

A total of 16 stakeholders participated in the pre-workshop survey. A description of the respondents is provided in **Appendix D**. Survey respondents represented 19 different districts across Uganda with Kampala and Mityana being the most highly represented regions (37.5% and 25.0%, respectively). The survey respondents also varied in terms of the level of the healthcare system in which they were situated. Of note, 33% of respondents identified working in health centre III settings and 26.7% identified working in regional referral hospitals. Respondents represented various roles within the healthcare system. The category of 'MWs, senior nursing officers and nursing officers' and the category of 'policymakers, policy advisors and policy consultants' made up the majority of respondents (each representing 40% of respondents).

Prioritizing guidelines

All of the respondents (n=15) selected the prevention and treatment of PPH guideline as the highest priority for implementation in Uganda (**Table 1**). The majority of respondents (n=12) considered prevention and treatment of pre-eclampsia and eclampsia as the next most important guideline. The augmentation of labour guideline was considered the third priority (n=9), while respondents considered the guideline for induction of labour the lowest priority (n= 8) of the four guidelines.

Table 1. WHO maternal and perinatal guidelines ranked in order of importance by pre-workshop survey respondents

Guideline	Priority	N
Prevention and treatment of PPH	1 (highest priority)	15
	2	0
	3	0
	4 (lowest priority)	0
Prevention and treatment pre-eclampsia and eclampsia	1 (highest priority)	0
	2	12
	3	0
	4 (lowest priority)	2
Augmentation of labour	1 (highest priority)	0
	2	2
	3	9
	4 (lowest priority)	4
Induction of labour	1 (highest priority)	0
	2	1
	3	6
	4 (lowest priority)	8

Prioritizing recommendations within guidelines

Using a scale of 1 to 5 (where 1=highest priority and 5=lowest priority), survey respondents identified the five most important recommendations within each of the four guidelines and ranked them in order of importance for implementation in Uganda. Detailed results are presented in **Appendix E- H**.

Prevention and treatment of PPH guideline

Appendix E outlines the ranking of priority recommendations for the prevention and treatment of PPH guideline. The three recommendations deemed to be the highest priorities based on total score were:

- Use of uterotonics for the prevention of PPH during the third stage of labour is recommended for all births (total score= 47);
- In settings where skilled birth attendants are not present and oxytocin is unavailable, the administration of misoprostol (600 µg PO) by community healthcare workers and lay health workers is recommended for the prevention of PPH' (total score= 36); and
- Oxytocin (10 IU, IV/IM) is the recommended uterotonic drug for the prevention of PPH (total score= 34).

Prevention and treatment of pre-eclampsia and eclampsia guideline

Appendix F outlines the ranking of priority recommendations for the prevention and treatment of pre-eclampsia and eclampsia guideline. The three recommendations deemed to be the highest priorities based on total score were:

- Magnesium sulfate is recommended for the prevention of eclampsia in women with severe pre-eclampsia in preference to other anticonvulsants (total score= 46);
- Women with severe hypertension during pregnancy should receive treatment with antihypertensive drugs (total score= 34); and
- Magnesium sulfate is recommended for the treatment of women with eclampsia in preference to other anticonvulsants (total score= 32).

Augmentation of labour guideline

Appendix G outlines the ranking of priority recommendations for the Augmentation of labour guideline. The three recommendations deemed to be the highest priorities based on total score were:

- Active phase partograph with a four-hour action line is recommended for monitoring the progress of labour' was the highest priority recommendation (total score= 59);
- Encouraging the adoption of mobility and upright position during labour in women at low risk is recommended (total score= 34); and
- Digital vaginal examination at intervals of four hours is recommended for routine assessment and identification of delay in active labour (total score= 20).

Induction of labour guideline

Appendix H outlines the ranking of priority recommendations for the Induction of labour guideline. The three recommendations deemed to be the highest priorities based on total score were:

- Induction of labour is recommended for women with prelabour rupture of membranes at term (total score= 35);
- Induction of labour is recommended for women who are known with certainty to have reached 41 weeks (>40 weeks + 7 days) of gestation (total score= 30); and
- If prostaglandins are not available, intravenous oxytocin alone should be used for induction of labour. Amniotomy alone is not recommended for induction of labour (total score= 22).

In-person workshop

Demographics

A total of 34 stakeholders participated in the two-day in-person workshop. A description of the participants is provided in **Table 2**. Participants were well-distributed across role categories, with representation from each major stakeholder group. Individuals who made up the highest proportion of participants identified in the category of 'MWs, senior nursing officers and nursing officers' or 'physicians' (32.4% and 26.5%, respectively).

Table 2. Demographic information of workshop participants

Professional Role (categories are not mutually exclusive)	n (n=34)	%
Midwives/senior nursing officers/nursing officers	11	32.4%
Physicians	9	26.5%
NGOs and international organizations	6	17.6%
Directors/administrators	4	11.8%
Researchers/academics	3	8.8%
Policymakers/policy advisors/policy consultants	3	8.8%

Focus groups

Key discussion points have been synthesized across FGs and are organized into the following categories to reflect factors that influence implementation of priority guideline recommendations and operate at the level of: (1) the healthcare system; (2) the provider; and (3) the patient/community. For an overview of the major themes and sub-themes identified at each level, refer to **Figure 1** below.

Figure 1. Overview of FG discussion findings on barriers and facilitators to guideline implementation

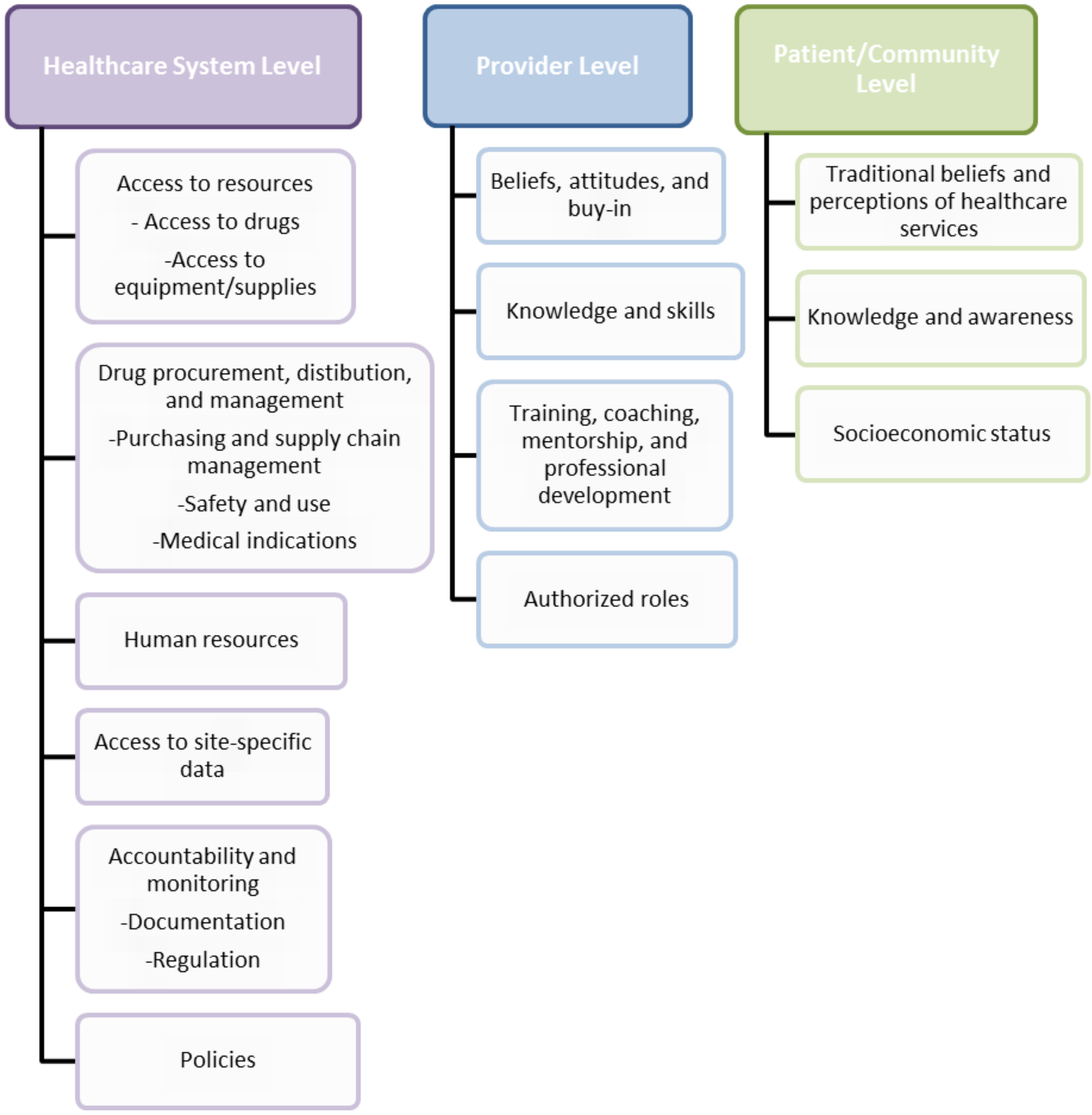

Factors affecting implementation of priority recommendations in the Ugandan context: Identifying barriers and facilitators

Healthcare system level

Various factors were identified to operate at the level of the healthcare system or be a product of systemic conditions in Uganda that can affect the implementation of the WHO guideline recommendations. These factors include: access to resources; drug procurement, distribution, and management; human resources; access to site-specific data; accountability and monitoring; and policies.

Access to resources

Participants in all three FGs described barriers experienced due to a lack of resources in healthcare facilities such as insufficient supplies of drugs and/or equipment/supplies. Though this barrier was noted to be present to some extent at all levels of the healthcare system, it was noted to be especially pronounced at lower level facilities (e.g., health centre levels II and III) and in rural/remote areas. Key discussion points largely focused on how access to resources can directly impede implementing guideline recommendations (i.e., a lack of necessary drugs or equipment/supplies required to implement the recommendation).

Lack of access to drugs

It was noted across FGs that not all medications are available equally at all levels of the healthcare system. For instance, access to magnesium sulfate was highlighted as an issue, particularly in lower level facilities. The antidote (i.e., calcium gluconate) to treat magnesium sulfate toxicity was also identified as a key commodity that is not widely available. Participants indicated that this can lead to further issues, as it may induce fear or avoidance of administering magnesium sulfate to prevent and treat pre-eclampsia or eclampsia if healthcare providers are not able to manage toxicity without the antidote available.

In cases where drugs are available (e.g., misoprostol, oxytocin, magnesium sulfate), participants identified that further challenges may remain such as calculations required to reconstitute magnesium sulfate before administration or access to clean/distilled water (e.g., for the dilution of oral misoprostol). There was consensus that access to oxytocin and misoprostol was generally sufficient. While this was noted as a facilitator, several participants also noted that the wide availability and distribution of these drugs can also lead to overuse or misuse in some settings.

Participants also cited examples of drugs being available but expired due to challenges with ordering/monitoring of stock at the facility or lack of refrigerators for proper storage of medications (e.g., oxytocin).

Lack of access to equipment/supplies

It was noted that lack of access to equipment and supplies was an issue in general, but is especially pronounced at lower level healthcare centres. Specific examples of equipment/supplies that are not widely available and directly affect implementation of the WHO guideline recommendations include lack of access to:

- Refrigerators for the proper storage of oxytocin;
- Partograph paper and/or fetoscope;

- Lack of blood pressure equipment such as the blood pressure cuff; and
- Correct sized syringes for magnesium sulfate administration.

One facilitator mentioned in relation to oxytocin storage, was the use of oxytocin that could be stored at room temperature. It was noted, however, that this type of oxytocin can only be stored at room temperature for 28 days reiterating the importance of diligence in terms of stocking and monitoring drugs at the facility level.

Drug procurement, distribution, and management

Purchasing and supply chain management

The market price of drugs was considered a factor that affects decisions surrounding procurement and distribution of drugs at the system level. As an example, it was noted that the market price of prostaglandins has increased in Uganda causing potential challenges with procuring misoprostol. There was debate around perceived cost of misoprostol- one FG commented on it being a relatively cheap drug, whereas another FG described the drug as "expensive". This debate may be a reflection of recent changes at the system level regarding the price of misoprostol.

The physician and IP groups described challenges they have experienced in their role within or external to the Ministry of Health Uganda to influence drug procurement practices such as challenges with getting buy-in from the senior officials to purchase, distribute or implement certain drug classes. Specifically, an example was provided around a recent effort to supply misoprostol to healthcare facilities (before healthcare workers had been trained and sensitized on its use), which resulted in expiry of the drug in some health facilities (having not been prescribed by the health workers). It was therefore difficult to justify asking the National Medical stores to procure misoprostol for healthcare facilities again. The second issue highlighted was that in spite of three local Ugandan studies (in Mbale, Mubende, and Kasese) that have demonstrated that community distribution of misoprostol for prevention of PPH in home births is safe, the Ministry of Health has failed to translate this evidence from research into practice. The technical team (MCH cluster) at the Ministry of Health Uganda have proposed distribution of misoprostol to women during antenatal care for self-use in cases where they are delivering at home. When this recommendation was discussed by the approving body, it was rejected due to concerns that misoprostol may be misused in the community.

Safety and use

A key focus of discussions across FGs was considerations of safety and appropriate usage of how drugs are administered and by whom. Misoprostol was the example provided most commonly to illustrate this point in the local context. Current policies indicate that misoprostol should be administered in facilities where physicians are present, as MWs have a limited role in induction of labour. It was also noted that some healthcare workers are not comfortable with administering misoprostol due to challenges with dosing (i.e., it is difficult to break tablets down into the correct dose). This issue was noted to be more pronounced in the case of administering vaginal misoprostol. Misoprostol is currently only available as a 200 µg tablet formulation in Uganda, however WHO recommends 25µg of vaginal misoprostol 6-hourly for induction, complicating its use.

Alternatively, one identified facilitator for correct dosing of oral misoprostol for induction was diluting the tablet in 200 mL of water and administering 25μg doses 2 hourly (as per WHO recommendations). This was challenged by some participants who noted that in some up-country settings, access to safe drinking water is limited. The physician group also noted that misoprostol should only be administered in settings with access to comprehensive surgical care (e.g., facilities/equipment/personnel available to perform a cesarean section) in the event of an emergency. This is not currently a policy directive. This arose from a concern that there were growing numbers of cases of induction of labour on a outpatient basis. It was not only being done in the wrong place (tablet inserted and women asked to go home and return the next day) but it was also being done by midwives who are currently not authorized to induce labour.

It was also noted that there have been cases where healthcare workers, in addition to augmenting labour with oxytocin, have given women misoprostol to further accelerate labour and this has, in some cases, been fatal with women dying from hemorrhage following rupturing of the uterus. The argument was also made that due to concerns of how misoprostol could be used in the community, oxytocin should be used as the first line of care in inducing labour as it has a shorter half-life and is generally safer; however, there was not clear consensus on this point. Finally, concerns that misoprostol will be misused in the community for unsafe termination of pregnancies remains a system-level barrier to its use.

Medical indications
Discussions around primary drug indications and how this affects implementation of recommendations in maternal care was less of a focus, but was mentioned specifically by the IP and physicians FGs. The physician group stated that healthcare providers are not universally aware of the indication for low-dose aspirin in the prevention of pre-eclampsia and therefore maternal wards do not generally stock for it. The IP group used the example of misoprostol to illustrate challenges associated with how drugs are registered and procurement processes. Specifically, it was noted that as misoprostol is not registered for use in induction of labour in Uganda (but rather it is registered for prevention of PPH and for post-abortion care), it cannot be procured for this purpose.

Human Resources
Lack of capacity due to shortages of healthcare providers (physicians and MWs) was described as a general barrier experienced throughout the healthcare system in Uganda. This barrier was described in terms of challenges with recruitment of physicians and MWs, especially in rural areas, leading to a disproportionate distribution of healthcare workers throughout the healthcare system (i.e., higher level facilities such as regional referral hospitals drawing more graduates than lower level facilities). Fiscal constraints at the national level were also described as a barrier to securing a full complement of healthcare providers in any given centre. Specific examples of how lack of capacity can affect implementation of WHO guidelines included an account of challenges experienced in complying with the recommendations pertaining to use of the partograph in centres with only one MW on duty and multiple patients on the ward. This can, in turn, result in inaccurate or lack of charting on a partograph, leading to potential complications due to improper monitoring/staging of labour. A second example given pertained to using uterine massage in compliance with the treatment of PPH recommendations. While this recommendation is qualified as a strong recommendation,

it is often not feasible to implement in practice due to lack of time and capacity for a healthcare provider to dedicate their attention to one patient.

Access to site-specific data

Two of the FGs (MW and IP groups) noted the need for more site-specific data on complications, death reviews, and implementation of evidence based practices (e.g., through audit and feedback in healthcare facilities). It was noted that access to site-specific data has acted as a facilitator to encourage practice change and increase buy-in from staff in the past as the usefulness/feasibility of guideline recommendations can be demonstrated in a specific setting. A specific example was given around use of partographs, whereby a study was conducted in certain facilities that showed that use of the partograph can in fact decrease time and increase efficiency versus slow practice down as is the common perception. This study was found to be influential in changing the attitudes and practices of staff at the sites in which it was conducted because the results felt relevant and meaningful to their context.

A common barrier observed by two of the three FGs (MWs and physicians) was that data are routinely collected and reported to the Ministry of Health Uganda but that the data are not fed back to the healthcare facilities to allow for site-specific analysis and/or continual improvements to be made in practice. The fact that channels for reporting are already established between the Ministry of Health Uganda and healthcare facilities was also seen as a facilitator or opportunity as these channels have the potential to allow two-way communication for sharing findings between the Ministry of Health and the various healthcare facilities.

Accountability and monitoring

Documentation

Participants identified lack of proper documentation in medical notes as a pervasive challenge in maternal healthcare practice. Additionally, participants noted that there are currently "no penalties" in place for failure to document patient care and that documentation is poorly monitored, if at all. The physician FG also noted that there is no emphasis placed on the importance of charting/documentation during training, fostering attitudes and an organizational culture that does not place high value on documentation. A facilitator that has improved patient charting practices was described by the MW FG; specifically, the presence of delivery books in all health facilities where the details of a mother's delivery are recorded. An opportunity to further best practices in documentation is being implemented in some healthcare facilities- the addition of columns in the delivery book to record certain interventions (e.g., administration of oxytocin or active management of the third stage of labour).

Regulation

A related barrier that was described by all three FGs was the need for improved accountability/monitoring of healthcare provider adherence to guidelines and professional standards. While it was acknowledged that there are regulators/inspectors in place, gaps and inefficiencies have been observed throughout the healthcare system. It was felt by all groups that there is a strong need to improve accountability of healthcare providers through routine monitoring by their professional associations and/or government bodies.

Policies

Participants across all FGs indicated that current government policies prohibit the implementation of some of the guideline recommendations, but also present opportunities for policies to be changed to better facilitate implementation of priority recommendations. Specific challenges were noted related to lack of evidence being considered in policy decisions that govern how drugs are distributed and used by various cadres of healthcare workers. For example, the physician and IP groups discussed that the administration of misoprostol by community healthcare workers is currently not supported by policy in Uganda, despite studies in Uganda that have demonstrated safety and effectiveness of this approach (under the supervision of midwives). This speaks to a larger challenge of using research evidence to change policy and that there is a current gap between the existing research evidence on misoprostol and policies around its use.

Summary of key barriers and facilitators at the healthcare system level

Barriers	Facilitators
• Lack of resources in healthcare facilities such as insufficient supplies of drugs (e.g., magnesium sulfate) and/or equipment/supplies (e.g., fetoscope, refrigerators).<br>• Difficulties with calculations required to reconstitute magnesium sulfate.<br>• Challenges with correct dosing of vaginal misoprostol.<br>• Gaps between registered medical indications of drugs and recommended uses in practice.<br>• Inefficiencies in drug procurement processes.<br>• Lack of facility capacity due to shortages of healthcare providers (e.g., physicians and midwives), especially in rural areas.<br>• Challenges with recruitment of healthcare workers (especially in rural areas).<br>• Fiscal constraints at the national level contributing to lack of healthcare providers in facilities.<br>• Data not routinely fed back from Ministry of Health to the healthcare facilities to allow for site-specific analysis and/or continual practice improvement.<br>• Lack of proper documentation in medical notes.<br>• Lack of accountability/monitoring of healthcare provider adherence to guidelines and professional standards.<br>• Lack of effective translation of research evidence into policy or practice (e.g., administration of misoprostol by community healthcare workers is currently not supported by policy in Uganda).	• Availability of oxytocin formulation that can be stored at room temperature.<br>• Ability to dilute oral misoprostol for lower-dose regimens (i.e., diluting the tablet in 200 mL of water).<br>• Access to site-specific data on some indicators to encourage practice change.<br>• Availability of delivery books in all healthcare facilities.

Provider level

Factors were also identified at the provider level to affect the implementation of priority WHO guideline recommendations in Uganda. These factors include: beliefs, attitudes and buy-in; knowledge and skills; training, coaching, mentorship, and professional development; and authorized roles.

Beliefs, attitudes, and buy-in

FGs identified the attitudes, beliefs, and buy-in of healthcare providers as factors that can either facilitate or hinder implementation of guideline recommendations in practice. One example used to illustrate this point was attitudes and beliefs around use of the partograph as a monitoring and grading tool in maternal care. Some healthcare workers (across various cadres) believe the partograph is ineffective and inefficient and therefore not a valuable use of time and resources. All three groups identified resistance to change as a barrier to implementing guideline recommendations. While this exists across cadres, it was noted that age is a mediating factor, whereby older/more experienced healthcare providers may be less accepting of new evidence. This point was emphasized around use of magnesium sulfate in the prevention and treatment of eclampsia as a relatively newer practice (since the early 2000s) where some healthcare providers may be resistant to its use. This barrier is further exacerbated when a particular belief or attitude is held by trainers/mentors who role model this view to their trainees. Participants also discussed how the experiences of a healthcare provider, including their organizational context, pre-clinical trainings, and anecdotal evidence influence how protocols/guidelines are operationalized or preference for one treatment or intervention over another regardless of what is recommended in a particular guideline (e.g., use of ergometrine over alternatives by the older MWs).

Knowledge and skills

Lack of knowledge or skills of some healthcare providers (across all cadres) was described as a general barrier to providing evidence-based maternal healthcare in Uganda. The most common example provided (given by all three FGs) to illustrate how lack of knowledge or skills can compromise use of particular guideline recommendation was around a lack of understanding of the extent of misuse of misoprostol. Another example of inappropriate use was cited under the augmentation of labour guideline with the use of intravenous (IV) oxytocin prior to confirmation of delay of labour. In either case it is believed that these drugs are used without an understanding of the potential harms. Lack of knowledge and skills was also cited as a factor that may prohibit providers from administrating magnesium sulfate in cases where it is indicated and a lack of skills on how to properly formulate the IV solution as it is not available in a user-friendly format, as well as monitor toxicity and administer the antidote if necessary. Other examples of priority guideline recommendations cited to be compromised by a lack of knowledge or skills include: lack of knowledge of the appropriate timeframe/rate in which oxytocin should be administered; lack of knowledge around *when* to augment labour; and lack of knowledge about how to properly use the partograph. However, there was less consensus on the reasons for not using the partograph, as some participants felt that it was well-known/well-taught but more of an issue of capacity and attitudes/beliefs whereas others attribute its underuse to lack of training.

Training, coaching, mentorship, and professional development

One of the most prominent barriers discussed in all three FGs was lack of opportunities for training, coaching, mentorship, and professional development available for healthcare providers. In Uganda there are no designated clinical instructors on the wards for some cadres of healthcare providers (e.g., MWs), meaning that the training curriculum does not continue beyond pre-clinical training. This lack of “continuous medical training at the bedside” was identified by participants as a barrier that contributes to new professionals not feeling confident and/or adequately prepared to use a given medication or intervention and may result in lack of compliance with guideline recommendations. In addition to improved access to training identified as a general need, specific areas were noted where training could be improved including: expanded surgical skills training (as an extension of internship); training in administration of the magnesium sulfate antidote; and introduction of training on how to interpret, adapt, and use guidelines as part of the current pre-clinical training curriculum. A need for diversified types of training was also noted including use of simulation (i.e., access to simulation centres) and emergency preparedness drills at the level of healthcare facilities.

When training is provided, it was mentioned that challenges still exist as there is a lack of emphasis placed on knowledge exchange or cascade training; those who do attend training (in-service training) do not routinely share their learnings with colleagues in their healthcare settings (e.g., lack of structures in place to share knowledge/learnings).

Authorized roles

Current role definitions of various cadres of healthcare providers in Uganda were thought to hinder the effective implementation of guideline recommendations in some cases. Opportunities for reviewing role definitions to facilitate task-shifting was seen as a promising opportunity. Another FG discussed the potential of training MWs to administer misoprostol for induction of labour or on the use of the balloon tamponade. It was further noted that some MWs are currently undergoing specialized training in the area of balloon tamponades which suggests that task-shifting is actively being explored as an implementation strategy in the Uganda context.

Summary of key barriers and facilitators at the provider level

Barriers	Facilitators
• Negative attitudes/beliefs about use of partograph (e.g., that it is inefficient or ineffective)<br>• Resistance to behaviour change to adhere to guideline recommendations<br>• Anecdotal evidence or provider preferences for medical practices/drugs that are not recommended in guidelines<br>• Lack of knowledge and skills (e.g., challenges with administering magnesium sulfate and properly formulating IV solution or using partograph)<br>• No designated clinical instructors on the wards for some cadres of healthcare providers (e.g., MWs)<br>• Insufficient diversity of training modes (e.g., access to simulation centres, emergency preparedness drills)<br>• Lack of emphasis on knowledge exchange activities (i.e., those who do attend in-service trainings do not routinely share their learnings with colleagues in their healthcare settings)<br>• Current role definitions of various cadres of healthcare workers hinder the effective implementation of guideline recommendations in some cases	• Opportunities for reviewing healthcare cadre role definitions to facilitate task-shifting<br>• Current specialized trainings offered to MWs in the area of balloon tamponades in some settings

Patient/community level

When considering how the patient/community can influence implementation of the WHO guideline recommendations, factors in the following categories were described: traditional beliefs; knowledge and awareness; and socioeconomic status.

Traditional beliefs and perceptions of healthcare services

The role of traditional cultural beliefs was identified as a community level factor that can influence healthcare-seeking behaviours of patients. One prominent example given is the traditional belief about the causes of eclampsia, and the resultant stigma associated with the condition. These beliefs and stigma have been reported to prevent patients from seeking care, complying with instructions to be transferred to a referral hospital, or taking the indicated medication (i.e., magnesium sulfate) once the condition is diagnosed. Alternatively, a factor that was identified in two of the FGs (MWs and physicians) as a facilitator fostered by cultural beliefs is the value placed on companionship and a sense of community in Uganda. This cultural value facilitates implementation of the recommendation of “a companion accompanying a woman during labour” (augmentation of labour guideline). Though this was identified as an important recommendation, a related

potential barrier was concern around a woman bringing a male companion as it could compromise the confidentiality and privacy of other patients on the ward. While this was acknowledged as a consideration, it was also noted that encouraging women to bring a female companion is a relatively easy and effective strategy to overcome this barrier.

Knowledge and awareness

Similar to one of the barriers identified at the provider level, lack of knowledge and awareness was also identified as a challenge that occurs at the patient/community level. For example, patients may demand misoprostol to increase the speed of their labour without the knowledge of the appropriate indications of this drug or the potential harms of misusing the drug. FGs also noted that some patients will not comply with practices or interventions as dictated by the guideline recommendations due to a lack of knowledge or awareness of the rationale and benefits. For example, the WHO augmentation of labour guideline recommends that women mobilize to enhance labour progression. Healthcare providers find that some patients are not aware of the benefits or purpose of mobilization and are therefore not compliant. It was also recognized that healthcare providers can contribute to this barrier as they do not always have the time or capacity to explain to the patient why they are being asked to mobilize.

Socioeconomic status

A pervasive barrier that was identified as a determinant of health-seeking behaviour at the patient/community level was the socioeconomic status of the patient. Lack of financial resources can deter or delay a patient from seeking healthcare, contributing to maternal morbidity and mortality rates in Uganda. For example, patients with financial constraints are perceived to be less likely to comply with a healthcare provider's recommendation to transfer to a referral hospital due to lack of access to transportation or resources to pay for transportation.

Summary of key barriers and facilitators at the patient/community level

Barriers	Facilitators
• Traditional beliefs about the causes of eclampsia<br>• Lack of awareness of benefits of some guideline recommendations (e.g., fear that mobilization will negatively affect labour) or harms of improper use of certain drugs<br>• Lack of financial resources (e.g., to pay for transportation)	• Positive cultural belief about the value of companionship

Individual ranking exercise

On **Day Two** of the in-person workshop, participants were asked to rank the feasibility of the 16 guideline recommendations that were deemed to be priorities in Uganda based on FG findings and deliberations among the workshop facilitators and in-country experts (**Day One)**. The 16 recommendations that were ranked included: 5 recommendations from Prevention and treatment of PPH; 4 recommendations from Prevention and treatment of pre-eclampsia and eclampsia; 3 recommendations from Induction of labour; and 4 recommendations from Augmentation of labour. Tasks ranked as most feasible were described by workshop participants to be the easiest to operationalize. Those ranked as least feasible were recommendations that were described as complicated, introduced safety concerns, or were in direct opposition with current policies or practices. Three of the 16 recommendations were re-ranked due to disparate responses. All results are presented in **Appendix I**. The following three recommendations were deemed to be most feasible to implement in the Ugandan context, with a median score of 9 ("extremely feasible"):

- The use of uterotonics for the prevention of PPH during the third stage of labour is recommended for all births. (Prevention and treatment of PPH guideline).
- Encouraging the adoption of mobility and upright position during labour in women at low risk is recommended. (Augmentation of labour guideline).
- The use of oral misoprostol for labour augmentation is not recommended. (Re-rated recommendation; Augmentation of labour guideline).

Small group discussions

Workshop facilitators asked small groups to consider implementation strategies and activities for the 16 priority recommendations. Each of the three groups was assigned 1-2 guidelines to focus on during discussions; however, implementation strategies that were broadly applicable or extended beyond assigned guidelines could also be discussed. A summary of the specific barriers identified and potential implementation strategies as identified by workshop participants are presented in **Table 3**.

Table 3. Mapping potential guideline implementation strategies/activities to barriers

Level of barrier	Category of barrier	Potential implementation strategies/activities
Healthcare system	Access to resources	• Introduce cost-sharing scheme whereby patients and community members donate money to healthcare facilities to help cover the cost of resources such as equipment/supplies and drugs.<br>• Introduce a protocol for inter-facility sharing of vehicles/ambulances that can be used for patient transport/transfer.<br>• Designate refrigerators on wards for multi-purpose use (e.g., storage of vaccines and medications).<br>• Advocate (e.g., through professional associations) for mandatory equipment/supplies in all healthcare facilities (e.g., partographs, partograph paper, refrigerators, etc.).<br>• Advocate (e.g., through professional associations) for purchasing of supplementary equipment/supplies such as curtains for patient privacy and birthing cushions to augment labour.
	Drug procurement, distribution, and management	• Secure formulation of oxytocin that can be stored at room temperature to decrease number of cases where the drug becomes compromised due to improper storage.<br>• Develop protocols, secure necessary equipment, and designate staff roles to monitor and record drug orders and quality control at (1) the level of the healthcare facility to monitor drug use and ordering needs; and (2) at the national level to ensure drug orders are being delivered and to inform national drug ordering practices.
	Human resources	• Eliminate government-imposed recruitment ban at national level to permit recruitment of healthcare workers across the country.<br>• Promote bonding schemes (encouraging healthcare workers to return to work in their communities) at all levels.<br>• Create competitive salaries, affordable housing, and schools in rural/remote communities to incentivize healthcare workers to work in underrepresented areas (i.e., lower level healthcare facilities).<br>• Review staffing law and implement a redistribution policy to ensure all healthcare facilities are operating at the recommended staff complement.<br>• Create incentives and programs to retain MWs and other healthcare workers who are approaching retirement (e.g., a proposal is being submitted to develop programs that train MWs in business administration to encourage retired MWs to take on healthcare administrative roles, or be trainers/supervisors).
	Access to site-specific data	• Conduct research studies to address the concerns of HPAC<br>• Advocate for Ministry of Health to feedback data to healthcare facilities for continuous quality improvement<br>• Introduce audit and feedback practices at healthcare facilities
	Accountability and monitoring	• Create protocols for documentation at the level of the healthcare facility<br>• Create protocols for MWs to document administration of magnesium sulfate before transfer to higher level facility<br>• Create interdisciplinary quality improvement teams at every healthcare facility to monitor and regulate practice to ensure guideline recommendations are being implemented<br>• Mandate emergency response/preparedness teams to be formed at all healthcare facilities<br>• Recruit and train MWs to specialize in/champion guideline implementation at larger healthcare facilities<br>• Create a schedule whereby an external inspector/Ministry of Health representative visits healthcare facilities at a determined frequency (e.g., mandatory minimum # of visits)<br>• Develop a policy that requires all healthcare facilities to have an intake procedure that records all visitors and patients to monitor who is present on a ward at any given time
	Policies	• Establish local demonstration sites to gain a deeper understanding of how the distribution of misoprostol to women during

Level of barrier	Category of barrier	Potential implementation strategies/activities
		antenatal care for self-use when delivery from home could be implemented as a policy, what the costs would be to Ministry of Health, and what the benefits would be (opposed to promoting current policy)<br>• Conduct a research study at the national level to evaluate the effectiveness of a cost-sharing scheme to provide funds to healthcare facilities and inform a policy directive<br>• Invest resources into professional associations to ensure they have the capacity and skills to effectively advocate for change on behalf of the healthcare workers they represent and support changes in practice by distributing/ disseminating information.
Provider	Beliefs, attitudes, and buy-in	• Create an organizational culture that promotes and supports accountability to professional standards and guidelines (e.g., if a physician frequently asks a MW for reports on a patient's partograph, this encourages the MW to use the partograph routinely).
	Knowledge and skills	• Develop protocols based on the WHO guidelines that are user-friendly, ready-to-use, and visible (e.g., posted on wards) to act as reminders for healthcare workers.
	Training, coaching, and professional development	• Develop and implement orientation programs for new staff to orient them to the healthcare facilities policies, protocols, expectations, and use of all equipment available.<br>• Establish coaching programs between more experienced and less experienced healthcare workers.<br>• Include a course on "how to use guidelines in practice" in university and college curricula for all healthcare workers.<br>• Promote bedside teaching as a valued teaching technique<br>• Improve supervision by ward managers/supervisors by providing training on WHO guidelines.<br>• Advocate for more training on surgical skills.<br>• Train healthcare workers on appropriate, effective, and transparent communication with patients.<br>• Create standardized modules for select staff members to go through to train them as supervisors or clinical instructors. A standardized curriculum will support awareness of guidelines.
	Scope of roles	• Create better linkages between healthcare facilities and village health teams (VHTs) as they are a key linkage to community healthcare<br>• Designate a liaison at every healthcare facility to be a "VHT coordinator" to standardize the use of WHO guidelines and better coordinate care<br>• Advocate for task-shifting among cadres of healthcare workers
Patient/ Community	Traditional beliefs	• Develop community level strategies (e.g., community meetings, community talks, use of SMS campaigns) to change attitudes/ de-stigmatize causes of eclampsia.<br>• Continue to promote importance of companionship during labour.
	Knowledge and awareness	• Develop education materials that are intended for patients (e.g., posters in wards on importance of mobility and examples of positions that promote labour) and for their companions (e.g., a one-pager on what to expect as a labour companion)<br>• Encourage patients to bring their designated companion to antenatal care visits<br>• Continue to use and promote the use of patient birth plans<br>• Develop community level strategies (e.g., community meetings, community talks, radio and/or SMS campaigns) to increase public awareness about benefits and potential harms of misuse of maternal commodities.
	Socioeconomic status	• Promote and create community-driven initiatives to share available resources at the community level (e.g., community fund for health emergencies or sharing a vehicle for hospital transport/transfer).

Limitations

There are three main limitations to the data collected. First, data were collected from a small sample of participants that may not be representative of the entire population working in the MNH sector of Uganda. Steps were taken, however, to ensure the sample was diverse and included representation from all or most major stakeholder groups and from multiple regions across the country. It should be noted that no patients or community members were included in any of the data collection activities. Similarly, none of the survey respondents were researchers or staff of non-governmental/international organizations and the survey response rate was poor. Second, time, resource, and space restrictions were faced by project organizers in conducting this activity; therefore, a purposeful convenience sample was used to identify stakeholders to participate in the pre-workshop survey and in-person workshop. Finally, cultural barriers and the use of external ("out-of-country") facilitators may have prevented a more robust understanding of the data. To reduce the impact of this limitation, in-country experts were consulted throughout the process to enhance comprehension of the data and its relevance to the local context.

Recommendations to inform a country-specific implementation plan

Multiple recommendations emerged from the pre-workshop and workshop findings that require action at the policy, research, and practice levels. The recommendations are presented in **Figure 2,** with distinction among levels of action (i.e., policy, research, practice) and timelines for action (i.e., shorter and longer-term goals).

Figure 2. Recommendations for future directions in Uganda, stratified by shorter- and longer-term actions for policy, research and practice areas.

RESEARCH	POLICY	PRACTICE
**Shorter-term actions:**<br><br>Conduct an audit of misoprostol use in healthcare facilities.<br><br>Engage researchers to develop a guideline implementation process/outcomes evaluation plan for the prioritized recommendations.	**Shorter-term actions**:<br><br>Review current drug ordering policies and drug quality regulations.<br><br>Conduct an audit of staffing complement at all healthcare facilities.<br><br>Review role definitions of all healthcare worker cadres and village health teams.	**Shorter-term actions:**<br><br>Professional associations to distribute latest evidence<br><br>Develop site-specific, user-friendly protocols based on WHO guidelines at level of healthcare facility.<br><br>Identify healthcare workers to form quality improvement teams at every healthcare facility to champion and monitor guideline use.<br><br>Develop educational materials for patients and companions.<br><br>Increase opportunities for training at the bedside, informal coaching programs, and new staff orientation.
**Longer-term actions:**<br><br>Conduct a national study on misuse of misoprostol and related harms (e.g., prevalence of PPH).<br><br>Develop an evidence-brief for policymakers based on results of study to inform changes to misoprostol regulation.	**Longer-term actions:**<br><br>Implement updated policies on drug procurement and distribution based on reviews.<br><br>Lift ban on recruitment of healthcare workers.<br><br>Implement redistribution policy to ensure all healthcare facilities are meeting recommended staff complement.<br><br>Create incentive programs and designated housing for healthcare workers in rural/remote areas.<br><br>Recruit and train more inspectors; create policy for frequency of site visits and regulations for practice (including penalties for not complying with professional standards/guidelines).	**Longer-term actions:**<br><br>Recruit and train onsite clinical instructors and supervisors<br><br>Develop plan and evaluate impacts of quality improvement teams.<br><br>Create formal linkages between healthcare facilities and village health teams.<br><br>Make continuing medical education and knowledge of current guideline recommendations a prerequisite for renewing annual practice licenses.

Summary and conclusions

The process of selecting priority maternal and perinatal health recommendations and exploring barriers and facilitators to implementation of the four priority WHO guidelines yielded valuable information to inform implementation planning in Uganda. The findings of the pre-workshop survey aligned with those of the in-person workshop, with the workshop providing an opportunity to explore perceptions and priorities in greater depth. Both data collection methods helped to inform concrete strategies for moving forward in facilitating the implementation of priority guideline recommendations in the local context. The most salient points that emerged across the pre-workshop and workshop activities were as follows:

- There is a need and an opportunity to improve implementation of the priority recommendations across the four selected WHO guidelines.
- Drug procurement, management, and distribution practices are not operating at an optimal level. Examples were provided of drugs expiring on the shelves before they are used and of insufficient drug supplies. This suggests a need for a national and facility level review of current drug procurement and monitoring policies/practices.
- There is concern around the potential misuse of misoprostol. Further research is required to better understand how misoprostol can be safely used in the community, to understand the extent and types of misoprostol misuse currently going on and how to improve use of misoprostol in health facilities in Uganda. Results of this research could, in turn, support changes to policy.
- Recruitment of more healthcare workers (physicians and MWs) is needed across the healthcare system, but particularly in rural/remote areas; infrastructure (e.g., housing for healthcare workers, schools for the children of healthcare workers, etc.) and incentives (i.e., a competitive salary comparable to urban centres) are needed as a preliminary step. Eliminating the current recruitment ban on the hiring of physicians and MWs in Uganda may also be considered as an opportunity to increase access to human resources and ultimately frontline capacity to implement guideline recommendations.
- There is a need to create more formal linkages between healthcare facilities and village health teams to better coordinate and standardize maternal healthcare for the community.
- Patients and the wider community (e.g., family members and caregivers) would benefit from increased awareness about the harms and benefits of certain guideline recommendations (e.g., benefits of a companion during labour; medical causes of eclampsia). This could be achieved through strategies and activities directed at patients and the wider community (e.g., radio/SMS campaigns, birth plans, educational materials, community talks/meetings).
- Increased opportunities for training is essential to improving the implementation of guideline recommendations including: onsite orientation for new staff; use of simulation training/simulation centres; training at the bedside; improved supervision; use of clinical instructors; and development of coaching programs between more experienced healthcare workers and new professionals.

It should be noted that the recommended (shorter-term) action for practice to form quality improvement teams at every healthcare facility is not well-supported by the research literature as an effective implementation strategy on its own[17]. We therefore further recommend that the formation of quality improvement teams be used in conjunction with other evidence-based strategies.

The methods used to inform the implementation strategies discussed in this report are transferable to other priority areas and other guidelines, particularly those in the area of MNH. Many of the barriers, facilitators, and resultant implementation strategies identified regarding the four WHO maternal and perinatal guidelines are applicable to other priority areas in healthcare; therefore, these findings can inform and be integrated into future barrier and facilitator assessments and guideline implementation planning initiatives in Uganda.

To facilitate guideline implementation, as informed by the findings presented in this report, we recommend creating a working group for planning, championing, and evaluating guideline implementation activities in Uganda. This working group would benefit from multidisciplinary participation and representation from multiple levels and geographic regions across the country, including (but not limited to): Ministry of Health Uganda staff, representatives of professional associations and regulatory bodies, physicians, MWs, community health partners, and researchers. Implementation support in terms of training and coaching can be provided by the GREAT Network throughout the process to aid in-country stakeholders in achieving implementation goals.

References

1. Straus SE, Moore JE, Uka S, Marquez C, Gülmezoglu AM: **Determinants of implementation of maternal health guidelines in Kosovo: Mixed methods study**. Imp Sci 2013, **8**:108.

2. McGlynn EA, *et al*.: **The quality of healthcare delivered to adults in the US**. *N Engl J Med* 2003, 348:2635-2645.

3. Davis D, Evans M, Jadad A, Perrier L, Rath D, Ryan D, Sibbald G, *et al*.: **The case for KT: shortening the journey from evidence to effect.** *BMJ* 2003, **327**(7405)**:**33-35.

4. Madon T, Hofman KJ, Kupfer L, Glass RI: **Public health. Implementation science.** *Science* 2007, **318**(5857)**:**1728-1729.

5. Shah BR, Mamdani M, Jaakkimainen L, Hux JE: **Risk modification for diabetic patients. Are other risk factors treated as diligently as glycemia?** *Can J Clin Pharmacol* 2004; **11**(2):e239- e244.

6. Pimlott NJ, Hux JE, Wilson LM, Kahan M, Li C, Rosser WW: **Educating physicians to reduce benzodiazepine use by elderly patients: a randomized controlled trial**. *CMAJ* 2003; **168**(7):835-839.

7. Kennedy J, Quan H, Ghali WA, Feasby TE: **Variations in rates of appropriate and inappropriate carotid endarterectomy for stroke prevention in 4 Canadian provinces**. *CMAJ* 2004; **171**(5):455- 459.

8. Haines A, Kuruvilla S, Borchert M: **Bridging the implementation gap between knowledge and action for health**. *Bull World Health Organ* 2004, **82**(10):724- 731.

9. World Health Organization: **Trends in maternal mortality: 1990 to 2010 WHO, UNICEF, UNFPA, and the World Bank Estimates.** https://www.unfpa.org/webdav/site/global/shared/documents/publications/2012/Trends_in_maternal_mortality_A4-1.pdf Accessed 12 Sep 2014.

10. World Health Organization: **WHO recommendations for the prevention and treatment of postpartum haemorrhage 2012**. http://www.who.int/reproductivehealth/publications/maternal_perinatal_health/9789241548502/en/ Accessed 12 Sep 2014.

11. World Health Organization: **WHO recommendations for the prevention and treatment of pre-eclampsia and eclampsia 2011**. http://www.who.int/reproductivehealth/publications/maternal_perinatal_health/9789241548335/en/ Accessed 12 Sep 2014.

12. World Health Organization: **WHO recommendations for induction of labour 2011**. http://www.who.int/reproductivehealth/publications/maternal_perinatal_health/9789241501156/en/ Accessed 12 Sep 2014.

13. World Health Organization: **WHO recommendations for augmentation of labour 2014**. http://www.who.int/reproductivehealth/publications/maternal_perinatal_health/augmentation-labour/en/ Accessed 12 Sep 2014.

14. Jones J, Hunter D: **Consensus methods for medical and health services research.** *BMJ* 1995, **311:**376-380.

15. Fitch K, *et al*.: **The RAND/UCLA appropriateness method user's manual**. RAND Corporation; 2001.

16. Braun V, Clarke V: **Using thematic analysis in psychology**. *Qual Res in Psychol* 2006; **3**(2): 77-101.

17. White DE *et al*.: **What is the value and impact of quality safety teams? A scoping review.** *Implement Sci* 2011; **6**(97).

18. Kamwesiga J: **Uganda healthcare system**. Kampala, Uganda: Makerere University, May 2011. http://docs.mak.ac.ug/sites/default/files/Health%20care%20system%20in%20Uganda2011.pdf Accessed 28 Nov 2014.

Appendix A: Uganda's healthcare system structure

Uganda's healthcare system runs on a referral basis; if a lower level facility (e.g., health centre II) cannot handle a case, it refers a patient to a unit the next level up. Each level of the healthcare system is defined below.

Level	Definition
Village health teams/community medicine distributors	The first contact for someone living in a rural area would be a medicine distributor or a member of a village health team (VHT). Each village is supposed to have these volunteers using bicycles. They have no access to medicine, but they can advise patients and refer them to health centres.
Health centre II	According to the Ugandan government's health policy, every parish is supposed to have one of these centres. A health centre II facility, serving a few thousand people, should be able to treat common diseases like malaria. It is supposed to be led by an enrolled nurse, working with a midwife. It runs an out-patient clinic, treating common diseases and offering antenatal care.
Health centre III	This facility should be found in every sub-county in Uganda. These centres should have about 18 staff, led by a senior clinical officer. It should also have a functioning laboratory.
Health centre IV/district hospital	This level of health facility serves a county. In addition to services found at health centre III, it should have wards for men, women, and children and should be able to admit patients. It should have a senior medical officer and another doctor as well as a theatre for carrying out emergency operations.
Regional referral hospital	There are 10 RRH which should have all the services offered at a health centre IV, plus specialized clinics – such as those for mental health and dentistry – and consultant physicians.
National referral hospital	At the top of the healthcare chain is the national referral hospital, located at Mulago in the capital Kampala.

Source: Adopted from Makerere University[18]

Appendix B: Pre-workshop survey

GREAT Project Assessment Survey- Uganda

Introduction

Welcome to the GREAT Project (Guideline-driven, Research priorities, Evidence synthesis, Application of evidence, and Transfer of knowledge). The purpose of the project is to improve the quality of care for mothers and infants in Uganda; to build capacity locally; and to develop a tailored strategy to implement the following four priority World Health Organization (WHO) guidelines on maternal and perinatal health: Prevention and Treatment of Postpartum Haemorrhage (2012) Prevention and Treatment of Pre-eclampsia and Eclampsia (2011) Induction of Labour (2011) Augmentation of Labour (2014)You are being invited to participate in a short survey to help the project team better understand the key priorities related to the identified WHO guidelines on maternal and perinatal health in the Ugandan context. Participation in the survey will take approximately 15- 20 minutes of your time. Survey responses are anonymous and will be used to inform the proceedings of a two-day in-person workshop to be held in Entebbe, Uganda in August 2014. By completing and submitting this survey, your consent to participate is implied. If you have any questions about the survey, please contact one of the following individuals: Dr. Denise Njama-Meya, PATH Uganda, dnjama@path.org Dr. Kidza Mugerwa, Makerere University, kidzamugerwa@yahoo.com Thank you very much for your time and participation.

Section 1: Demographic Information

1. In which district, county/municipality, AND/OR sub-county/village do you work? Please respond in the box provided below.

District: [ ]

Sub-district [ ]

2. At what level of the healthcare system do you work? Please check all responses that apply.

- Health centre II ☐
- Health centre III ☐
- Health centre IV ☐
- District/ general hospital ☐
- District health office ☐
- Regional referral hospital ☐
- National referral hospital ☐
- Ministry of Health headquarters ☐
- Non-government/ international organization ☐
- Professional regulatory body/ professional organization (e.g nursing association) ☐
- Other (please specify in question 3) ☐

3. What is your title/role description?

[ ]

4. How long have you been in this role?

- ○ Less than 1 year
- ○ 1-2 years
- ○ 3-5 years
- ○ 6-10 years
- ○ 11-20 years
- ○ More than 20 years

5. Please list up to five main tasks you carry out in your current job, in descending order, starting from the most common. Please respond in the box provided below.

1. [ ]
2. [ ]
3. [ ]
4. [ ]
5. [ ]

<u>Section 2: Guideline Prioritization</u>

In this section you are being asked to rate the priority of each of the four selected guidelines, relative to one another. Of the following four areas identified for the WHO maternal and perinatal guideline implementation, please rank in order of highest priority to lowest priority from your perspective. To make your selections, simply drag the topic area from the left side of the page and drop it in to the fields on the right side of the page, in order of priority (1= highest priority, 4= lowest priority).

	1 (highest priority)	2	3	4 (lowest priority)
Prevention and treatment of postpartum haemorrhage (PPH)	○	○	○	○
Pre-eclampsia / eclampsia	○	○	○	○
Induction of labour	○	○	○	○
Augmentation of labour	○	○	○	○

<u>Section 3: Prioritization of Recommendations</u>

In this section, you are being asked to select the priority areas from a list of recommendations for each of the four selected WHO guidelines on maternal and perinatal health: Prevention and Treatment of Postpartum Haemorrhage (2012) Prevention and Treatment of Pre-eclampsia and Eclampsia (2011) Induction of Labour (2011) Augmentation of Labour (2014) These guidelines will appear in a random order, not necessarily the order you prioritized them.

Prevention and Treatment of Postpartum Haemorrhage (PPH)

From the list of recommendations below, taken from the WHO guideline on the prevention and treatment of postpartum haemorrhage (PPH), please select the <u>FIVE</u> recommendations that you feel are/should be priorities in Uganda at this time. To make your selections, simply drag the recommendation from the left side of the page and drop it in to the fields on the right side of the page, in order of importance, where 1 = most important and 5 - least important. The strength and quality of the recommendation are provided in brackets (Strength, Quality).

	1 (most important)	2	3	4	5
The use of uterotonics for the prevention of PPH during the third stage of labour is recommended for all births (Strong, Moderate)	○	○	○	○	○
Oxytocin (10 IU, IV/IM) is the recommended uterotonic drug for the prevention of PPH (Strong, Moderate)	○	○	○	○	○
In settings where oxytocin is unavailable, the use of other injectable uterotonics (if appropriate ergometrine/ methylergometrine or the fixed drug combination of oxytocin and ergometrine) or oral misoprostol (600 µg) is recommended. (Strong, Moderate)	○	○	○	○	○
In settings where skilled birth attendants are not present and oxytocin is unavailable, the administration of misoprostol (600 µg PO) by community healthcare workers and lay health workers is recommended for the prevention of PPH.(Strong, Moderate)	○	○	○	○	○
Late cord clamping (performed after 1 to 3 minutes after birth) is recommended for all births while initiating simultaneous essential newborn care (Strong, Moderate)	○	○	○	○	○
Early cord clamping (<1 minute after birth) is not recommended unless the neonate is asphyxiated and needs to be moved immediately for resuscitation (Strong, Moderate)	○	○	○	○	○
Postpartum abdominal uterine tonus assessment for early identification of uterine atony is recommended for all women (Strong, Very low)	○	○	○	○	○
Oxytocin (IV or IM) is the recommended uterotonic drug for the prevention of PPH in caesarean section (Strong, Moderate)	○	○	○	○	○
Controlled cord traction is the recommended method for removal of the placenta in caesarean section (Strong, Moderate)	○	○	○	○	○
Intravenous oxytocin alone is the recommended uterotonic drug for the treatment of PPH (Strong, Moderate)	○	○	○	○	○
If intravenous oxytocin is unavailable, or if the bleeding does not respond to oxytocin, the use of intravenous ergometrine, oxytocin-ergometrine fixed dose, or a	○	○	○	○	○

prostaglandin drug (including sublingual misoprostol, 800 µg) is recommended. (Strong, low)					
The use of isotonic crystalloids is recommended in preference to the use of colloids for the initial intravenous fluid resuscitation of women with PPH (Strong, low)	○	○	○	○	○
Uterine massage is recommended for the treatment of PPH (Strong, very low)	○	○	○	○	○
If women do not respond to treatment using uterotonics, or if uterotonics are unavailable, the use of intrauterine balloon tamponade is recommended for the treatment of PPH due to uterine atony. (Weak, very low)	○	○	○	○	○
If bleeding does not stop in spite of treatment using uterotonics and other available conservative interventions (e.g. uterine massage, balloon tamponade), the use of surgical interventions is recommended (Strong, very low)	○	○	○	○	○
The use of bimanual uterine compression is recommended as a temporizing measure until appropriate care is available for the treatment of PPH due to uterine atony after vaginal delivery. (Weak, very low)	○	○	○	○	○
The use of uterine packing is not recommended for the treatment of PPH due to uterine atony after vaginal birth (Weak, very low)	○	○	○	○	○
If the placenta is not expelled spontaneously, the use of IV/IM oxytocin (10 IU) in combination with controlled cord traction is recommended (Weak, very low)	○	○	○	○	○
A single dose of antibiotics (ampicillin or first-generation cephalosporin) is recommended if manual removal of the placenta is practised. (Weak, very low)	○	○	○	○	○
The use of formal protocols by health facilities for the prevention and treatment of PPH is recommended (Weak, moderate)	○	○	○	○	○
The use of formal protocols for referral of women to a higher level of care is recommended for health facilities (Weak, very low)	○	○	○	○	○
Monitoring the use of uterotonics after birth for the prevention of PPH is recommended as a process indicator for programmatic evaluation (Weak, very low)	○	○	○	○	○

<u>Section 3: Prioritization of Recommendations</u>

In this section, you are being asked to select the priority areas from a list of recommendations for each of the four selected WHO guidelines on maternal and perinatal health: Prevention and Treatment of Postpartum Haemorrhage (2012) Prevention and Treatment of Pre-eclampsia and Eclampsia (2011) Induction of Labour (2011) Augmentation of Labour (2014) These guidelines will appear in a random order, not necessarily the order you prioritized them.

Prevention and Treatment of Pre-Eclampsia and Eclampsia

From the list of recommendations below, taken from the WHO guideline on the prevention and treatment of pre-eclampsia and eclampsia, please select the <u>FIVE</u> recommendations that you feel are/should be priorities in Uganda at this time. To make your selections, simply drag the recommendation from the left side of the page and drop it in to the fields on the right side of the page, in order of importance, where 1 = most important and 5 - least important. The strength and quality of the recommendation are provided in brackets (Strength, Quality).

	1 (most important)	2	3	4	5
In areas where dietary calcium intake is low, calcium supplementation during pregnancy (at doses of 1.5–2.0 g elemental calcium/day) is recommended for the prevention of pre-eclampsia in all women, but especially those at high risk of developing pre-eclampsia (Strong, moderate)	○	○	○	○	○
Low-dose acetylsalicylic acid (aspirin, 75 mg) is recommended for the prevention of pre-eclampsia in women at high risk of developing the condition. (Strong, moderate)	○	○	○	○	○
Women with severe hypertension during pregnancy should receive treatment with antihypertensive drugs. (Strong, very low)	○	○	○	○	○
Magnesium sulfate is recommended for the prevention of eclampsia in women with severe pre-eclampsia in preference to other anticonvulsants. (Strong, high)	○	○	○	○	○
Magnesium sulfate is recommended for the treatment of women with eclampsia in preference to other anticonvulsants. (Strong, moderate)	○	○	○	○	○
The full intravenous or intramuscular magnesium sulfate regimens are recommended for the prevention and treatment of eclampsia. (Strong, moderate)	○	○	○	○	○
For settings where it is not possible to administer the full magnesium sulfate regimen, the use of magnesium sulfate loading dose followed by immediate transfer to a higher level health-care facility is recommended for women with severe pre-eclampsia and eclampsia. (Weak, very low)	○	○	○	○	○
Induction of labour is recommended for women with severe preeclampsia at a gestational age when the fetus is not viable or unlikely to achieve viability within one or two weeks. (Strong, very low)	○	○	○	○	○
In women with severe pre-eclampsia, a viable fetus and before 34 weeks of gestation, a policy of expectant management is recommended, provided that uncontrolled maternal hypertension, increasing maternal organ dysfunction or fetal distress are absent and can be monitored. (Weak, very low)	○	○	○	○	○
In women with severe pre-eclampsia, a viable fetus and between 34 and 36 (plus 6 days) weeks of gestation, a policy of expectant management may be recommended, provided that uncontrolled maternal hypertension, increasing maternal organ dysfunction or fetal distress are absent and can be monitored. (Weak, very low)	○	○	○	○	○
In women with severe pre-eclampsia at term, early delivery is recommended. (Strong, low)	○	○	○	○	○
In women with mild pre-eclampsia or mild gestational hypertension at term, induction of labour is recommended. (Weak, moderate)	○	○	○	○	○
In women treated with antihypertensive drugs antenatally, continued antihypertensive treatment postpartum is recommended. (Strong, very low)	○	○	○	○	○
Strict bedrest is not recommended for improving pregnancy outcomes in women with hypertension (with or without proteinuria) in pregnancy. (Weak, low)	○	○	○	○	○

Vitamin D supplementation during pregnancy is not recommended to prevent the development of preeclampsia and its complications. (Strong, very low)	○	○	○	○	○
Individual or combined vitamin C and vitamin E supplementation during pregnancy is not recommended to prevent the development of pre-eclampsia and its complications. (Strong, high)	○	○	○	○	○
Diuretics, particularly thiazides, are not recommended for the prevention of pre-eclampsia and its complications. (Strong, low)	○	○	○	○	○

<u>Section 3: Prioritization of Recommendations</u>

In this section, you are being asked to select the priority areas from a list of recommendations for each of the four selected WHO guidelines on maternal and perinatal health: Prevention and Treatment of Postpartum Haemorrhage (2012) Prevention and Treatment of Pre-eclampsia and Eclampsia (2011) Induction of Labour (2011) Augmentation of Labour (2014) These guidelines will appear in a random order, not necessarily the order you prioritized them.

Induction of Labour

From the list of recommendations below, taken from the WHO guideline for induction of labour, please select the <u>FIVE</u> recommendations that you feel are/should be priorities in Uganda at this time. To make your selections, simply drag the recommendation from the left side of the page and drop it in to the fields on the right side of the page, in order of importance, where 1 = most important and 5 - least important. The strength and quality of the recommendation are provided in brackets (Strength, Quality).

	1 (most important)	2	3	4	5
Induction of labour is recommended for women who are known with certainty to have reached 41 weeks (>40 weeks + 7 days) of gestation. (Weak, low)	○	○	○	○	○
If gestational diabetes is the only abnormality, induction of labour before 41 weeks of gestation is not recommended. (Weak, very low)	○	○	○	○	○
Induction of labour at term is not recommended for suspected fetal macrosomia. (Weak, low)	○	○	○	○	○
Induction of labour is recommended for women with prelabour rupture of membranes at term. (Strong, high)	○	○	○	○	○
If prostaglandins are not available, intravenous oxytocin alone should be used for induction of labour. Amniotomy alone is not recommended for induction of labour. (Weak, moderate)	○	○	○	○	○
Oral misoprostol (25 µg, 2-hourly) is recommended for induction of labour. (Strong, moderate)	○	○	○	○	○
Low-dose vaginal misoprostol (25 µg, 6-hourly) is recommended for induction of labour. (Strong, moderate)	○	○	○	○	○
Misoprostol is not recommended for induction of labour in women with previous caesarean section (Strong, low)	○	○	○	○	○

Low doses of vaginal prostaglandins are recommended for induction of labour. (Strong, moderate)	○	○	○	○	○
Balloon catheter is recommended for induction of labour (Strong, moderate)	○	○	○	○	○
The combination of balloon catheter plus oxytocin is recommended as an alternative method of induction of labour when prostaglandins (including misoprostol) are not available or are contraindicated. (Weak, low)	○	○	○	○	○
In the third trimester, in women with a dead or an anomalous fetus, oral or vaginal misoprostol are recommended for induction of labour. (Strong, low)	○	○	○	○	○
Sweeping membranes is recommended for reducing formal induction of labour. (Strong, moderate)	○	○	○	○	○
Betamimetics are recommended for women with uterine hyperstimulation during induction of labour. (Weak, low)	○	○	○	○	○

<u>Section 3: Prioritization of Recommendations</u>

In this section, you are being asked to select the priority areas from a list of recommendations for each of the four selected WHO guidelines on maternal and perinatal health: Prevention and Treatment of Postpartum Haemorrhage (2012) Prevention and Treatment of Pre-eclampsia and Eclampsia (2011) Induction of Labour (2011) Augmentation of Labour (2014) These guidelines will appear in a random order, not necessarily the order you prioritized them.

Augmentation of Labour

From the list of recommendations below, taken from the WHO guideline for augmentation of labour, please select the <u>FIVE</u> recommendations that you feel are/should be priorities in Uganda at this time. To make your selections, simply drag the recommendation from the left side of the page and drop it in to the fields on the right side of the page, in order of importance, where 1 = most important and 5 - least important. The strength and quality of the recommendation are provided in brackets (Strength, Quality).

	1 (most important)	2	3	4	5
Active phase partograph with a four-hour action line is recommended for monitoring the progress of labour. (Strong, very low)	○	○	○	○	○
Digital vaginal examination at intervals of four hours is recommended for routine assessment and identification of delay in active labour. (Weak, very low)	○	○	○	○	○
A package of care for active management of labour for prevention of delay in labour is not recommended. (Weak, low)	○	○	○	○	○
The use of early amniotomy with early oxytocin augmentation for prevention of delay in labour is not recommended. (Weak, very low)	○	○	○	○	○
The use of oxytocin for prevention of delay in labour in women receiving epidural analgesia is not recommended. (Weak, low)	○	○	○	○	○
The use of amniotomy alone for prevention of delay in labour is not recommended. (Weak, very low)	○	○	○	○	○
The use of antispasmodic agents for prevention of delay in labour is not	○	○	○	○	○

recommended. (Weak, very low)

The use of intravenous fluids with the aim of shortening the duration of labour is not recommended. (Strong, very low)

For women at low risk, oral fluid and food intake during labour is recommended. (Weak, very low)

Encouraging the adoption of mobility and upright position during labour in women at low risk is recommended. (Strong, very low)

Continuous companionship during labour is recommended for improving labour outcomes. (Strong, moderate)

The use of oxytocin alone for treatment of delay in labour is recommended. (Weak, very low)

Augmentation with intravenous oxytocin prior to confirmation of delay in labour is not recommended. (Weak, very low)

High starting and increment dosage regimen of oxytocin is not recommended for labour augmentation. (Weak, very low)

The use of oral misoprostol for labour augmentation is not recommended. (Strong, very low)

The use of amniotomy alone for treatment of delay in labour is not recommended (Weak, very low)

The use of amniotomy and oxytocin for treatment of confirmed delay in labour is recommended. (Weak, very low)

6. Are you aware of any other stakeholders in your community (for example members of the public, community leaders, healthcare managers, healthcare professionals, healthcare workers, policymakers, or key community members) who have knowledge of guideline implementation in the area of maternal and perinatal health and may be willing to participate in this survey?

Name 1:

Title/Role 1:

Email Address 1:

Mailing Address 1:

7. Is there anything that you would like to add?

Thank you very much for participating in this survey.

Appendix C: Focus group discussion guides

**<u>VERSION: PHYSICIANS & MIDWIVES</u>**	
**Question 1**	Keeping in mind the context of maternal and newborn health in Uganda at this time, what <u>three</u> recommendations are the most important to implement for the :<br><br>- Induction of labour guideline?<br>- Augmentation of labour guideline?<br>- Prevention and treatment of PPH guideline?<br>- Prevention and treatment of preeclampsia and eclampsia guideline?<br><br><u>Probes</u> (*note*: *use probes if the participants did not provide enough information in their responses to the above question*)<br><br>• Why did you select those specific guideline recommendations?<br>• What factors did you consider when selecting the guideline recommendations?
**Question 2**	Which of these recommendations are within your scope of practice?<br><br>In other words, which guideline recommendations would you perform as part of your professional role if they were implemented?
**Question 3**	How confident do you feel in your knowledge to practice these recommendations? In your skills? In your ability?<br><br><u>Probe</u><br><br>○ Please elaborate/expand on answer.<br>○ Do you think you will require additional training to perform these tasks as recommended by the WHO?
**Question 4**	Thinking about the top three recommendations for each guideline as a whole, what do you think are the potential barriers or challenges to implementing these guideline recommendations in Uganda?<br><u>Probes</u><br>○ What are some of the barriers or challenges at the systems level?<br>*Examples include funding, policy, healthcare structure, geography, current cultural and political climate in Uganda, etc.*<br>○ What are some of the barriers or challenges at the level of the healthcare provider?<br>*Examples include skills, attitudes/beliefs, leadership, interprofessional working climate, etc.*<br>○ What are some of the barriers or challenges at the level of the patients and communities?<br>*Examples include cultural beliefs, health seeking behaviours, preferences for care, etc.*

<table>
<tr><td>Question 5</td><td>Again, thinking about the top three recommendations for each guideline as a whole, what do you think are the potential facilitators that could aid in the implementation of these guidelines?

<u>Probes</u>
What are some of the facilitators at the systems level?
Examples include alignment with current initiatives, political turnover/opportunity, updating health training curricula, etc.
What are some of the facilitators at the level of the healthcare provider?
Examples include champions at each clinical level, strong leadership, reward systems/positive reinforcement, training, etc.
What are some of the facilitators at the level of the patients and communities?
Examples include cultural beliefs, health seeking behaviours, preferences for care, etc.</td></tr>
<tr><td>Question 6</td><td>Do you feel confident that these guideline recommendations can be adhered to? In other words, that the recommendations will be practiced and that practices will be sustained?

Probe
Please elaborate/expand on answer.</td></tr>
<tr><td>Question 7</td><td>How do you currently monitor/measure maternal and newborn health practices?

<u>Probe</u>
What kinds of data do you collect or record, and how do you collect/record this data?
In your opinion, is this a sufficient monitoring/measuring system?
How are people in your profession held accountable to professional standards in Uganda?</td></tr>
<tr><td>Question 8</td><td>Do you have any additional suggestions that could help with the implementation of the selected WHO guidelines?

Is there anything else that anyone would like to add?</td></tr>
<tr><td colspan="2">Thank participants and wrap up</td></tr>
</table>

VERSION: INTERPROFESSIONAL (IP) GROUP	
**Question 1**	Keeping in mind the context of maternal and newborn health in Uganda at this time, what <u>three</u> recommendations are the most important to implement for the:<br><br>- Induction of labour guideline?<br>- Augmentation of labour guideline?<br>- Prevention and treatment of PPH guideline?<br>- Prevention and treatment of preeclampsia and eclampsia guideline?<br><br><u>Probes</u> (*note*: *use probes if the participants did not provide enough information in their responses to the above question*)<br><br>• Why did you select those specific guideline recommendations?<br>• What factors did you consider when selecting the guideline recommendations?
**Question 2**	Thinking about the top three recommendations for each guideline as a whole, what do you think are the potential barriers or challenges to implementing these guideline recommendations in Uganda?<br><br><u>Probes</u><br>• What are some of the barriers or challenges at the systems level? *Examples include funding, policy, healthcare structure, geography, current cultural and political climate in Uganda, etc.*<br>• What are some of the barriers or challenges at the level of the healthcare provider? *Examples include skills, attitudes/beliefs, leadership, interprofessional working climate, etc.*<br>• What are some of the barriers or challenges at the level of the patients and communities? *Examples include cultural beliefs, health seeking behaviours, preferences for care, etc.*
**Question 3**	Again, thinking about the top three recommendations for each guideline as a whole, what do you think are the potential facilitators that could aid in the implementation of these guidelines?<br><br><u>Probes</u><br>• What are some of the facilitators at the systems level? *Examples include alignment with current initiatives, political turnover/opportunity, updating health training curricula, etc.*<br>• What are some of the facilitators at the level of the healthcare provider? *Examples include champions at each clinical level, strong leadership, reward systems/positive reinforcement, training, etc.*<br>• What are some of the facilitators at the level of the patients and communities? *Examples include cultural beliefs, health seeking behaviours, preferences for care,*

	etc.
**Question 4**	Do you feel that there is sufficient readiness and buy-in in Uganda to implement these guideline recommendations?<br><br><u>Probes</u><br>• If yes, please describe readiness for change at each level: health systems, providers, and patients/communities.<br>• If no, why not? What would be required to make Uganda more prepared to implement these guideline recommendations?
**Question 5**	Do you feel confident that these guideline recommendations can be adhered to? In other words, that the recommendations will be practiced and that practices will be sustained?<br><br><u>Probe</u><br><br>• Please elaborate/expand on answer.
**Question 6**	How do you currently monitor/measure implementation efforts?<br><br><u>Probe</u><br><br>o In your opinion, is this a sufficient monitoring/measuring system?<br>o Would you need to change anything in order to effectively monitor/measure the implementation of the WHO guidelines and associated outcomes?<br>o What groups/individuals would need be involved in the monitoring/measurement process, who aren't already engaged?
**Question 7**	Do you have any additional suggestions that could help with the implementation of the selected WHO guidelines?
**Question 8**	Before we wrap up today's discussion, is there anything else that anyone would like to add?
**Thank participants and wrap up**	

Appendix D: Demographic information of pre-workshop survey respondents

Region (categories are not mutually exclusive)	n (n=16)	%
Kampala	6	37.5%
Mityana	4	25.0%
Soroti	2	12.5%
Wakiso	2	12.5%
Kamuli	2	12.5%
Kaliro	2	12.5%
Mayuge	2	12.5%
Kumi	2	12.5%
Kayunga	2	12.5%
Luwero	2	12.5%
Nakasongola	2	12.5%
Mapigi	2	12.5%
Sembabule	2	12.5%
Kalangala	2	12.5%
Kyenjojo	2	12.5%
Kamwenge	2	12.5%
Kasese	2	12.5%
Jinja	1	6.3%
Bugiri	1	6.3%
**Level of the healthcare system (categories are not mutually exclusive)**	**n (n=15)**	**%**
Health Centre III	5	33.3%
Regional referral hospital	4	26.7%
Health Centre II	3	20.0%
Health Centre IV	2	13.3%
District/general hospital	2	13.3%
Ministry of Health headquarters	2	13.3%
Profession regulatory body/professional organization	2	13.3%
District/health office	1	6.7%
National referral hospital	1	6.7%
Non-government/international organization	1	6.7%
**Professional Role (categories are not mutually exclusive)**	**n (n=15)**	**%**
Midwives/ senior nursing officers/nursing officers	6	40.0%
Policymakers/policy advisors/policy consultants	6	40.0%
Directors/administrators	2	13.3%
Physicians	1	6.7%
Researchers/academics	0	0.0%
NGOs and international organizations	0	0.0%

Appendix E: Pre-workshop survey findings for priority recommendations for prevention and treatment of PPH guideline

Prevention and treatment of PPH	Priority	n	N Total	Total Score
The use of uterotonics for the prevention of PPH during the third stage of labour is recommended for all births (Strong, Moderate)	1 (highest priority)	8	10	47
	2	1		
	3	1		
	4	0		
	5 (lowest priority)	0		
In settings where skilled birth attendants are not present and oxytocin is unavailable, the administration of misoprostol (600 µg PO) by community healthcare workers and lay health workers is recommended for the prevention of PPH (Strong, Moderate)	1 (highest priority)	2	12	36
	2	1		
	3	4		
	4	5		
	5 (lowest priority)	0		
Oxytocin (10 IU, IV/IM) is the recommended uterotonic drug for the prevention of PPH (Strong, Moderate)	1 (highest priority)	2	8	34
	2	6		
	3	0		
	4	0		
	5 (lowest priority)	0		
In settings where oxytocin is unavailable, the use of other injectable uterotonics (if appropriate ergometrine/ methylergometrine or the fixed drug combination of oxytocin and ergometrine) or oral misoprostol (600 µg) is recommended. (Strong, Moderate)	1 (highest priority)	0	7	22
	2	2		
	3	4		
	4	1		
	5 (lowest priority)	0		

*Note: The number of respondents who identified a given recommendation within each of the five categories = n; the total number of respondents who selected the recommendation as one of their top five priorities = N Total; the calculated total score = Total Score. The total number of respondents (N Total) varies as it is dependent on the number of respondents who selected a given recommendation among their top five priorities.

Appendix F: Pre-workshop survey findings for priority recommendations for prevention and treatment of pre-eclampsia and eclampsia guideline

Prevention and treatment of pre-eclampsia and eclampsia	Priority	n	N Total	Total Score
Magnesium sulfate is recommended for the prevention of eclampsia in women with severe pre-eclampsia in preference to other anticonvulsants. (Strong, high)	1 (highest priority)	7	10	46
	2	2		
	3	1		
	4	0		
	5 (lowest priority)	0		
Women with severe hypertension during pregnancy should receive treatment with antihypertensive drugs. (Strong, very low)	1 (highest priority)	3	10	34
	2	2		
	3	2		
	4	2		
	5 (lowest priority)	1		
Magnesium sulfate is recommended for the treatment of women with eclampsia in preference to other anticonvulsants. (Strong, moderate)	1 (highest priority)	2	10	32
	2	2		
	3	2		
	4	4		
	5 (lowest priority)	0		
For settings where it is not possible to administer the full magnesium sulfate regimen, the use of magnesium sulfate loading dose followed by immediate transfer to a higher level health-care facility is recommended for women with severe pre-eclampsia and eclampsia. (Weak, very low)	1 (highest priority)	1	8	25
	2	2		
	3	2		
	4	3		
	5 (lowest priority)	0		

*Note: The number of respondents who identified a given recommendation within each of the five categories = n; the total number of respondents who selected the recommendation as one of their top five priorities = N Total; the calculated total score = Total Score. The total number of respondents (N Total) varies as it is dependent on the number of respondents who selected a given recommendation among their top five priorities.

Appendix G: Pre-workshop survey findings for priority recommendations for augmentation of labour guideline

Augmentation of labour	Priority	n	N Total	Total Score
Active phase partograph with a four-hour action line is recommended for monitoring the progress of labour. (Strong, very low)	1 (highest priority)	11	12	59
	2	1		
	3	0		
	4	0		
	5 (lowest priority)	0		
Encouraging the adoption of mobility and upright position during labour in women at low risk is recommended. (Strong, very low)	1 (highest priority)	1	10	34
	2	4		
	3	3		
	4	2		
	5 (lowest priority)	0		
Digital vaginal examination at intervals of four hours is recommended for routine assessment and identification of delay in active labour. (Weak, very low)	1 (highest priority)	0	7	20
	2	2		
	3	3		
	4	1		
	5 (lowest priority)	1		

*Note: The number of respondents who identified a given recommendation within each of the five categories = n; the total number of respondents who selected the recommendation as one of their top five priorities = N Total; the calculated total score = Total Score. The total number of respondents (N Total) varies as it is dependent on the number of respondents who selected a given recommendation among their top five priorities.

Appendix H: Pre-workshop survey findings for priority recommendations for induction of labour guideline

Induction of labour	Priority	n	N Total	Total Score
Induction of labour is recommended for women with prelabour rupture of membranes at term. (Strong, high)	1 (highest priority)	3	9	35
	2	3		
	3	2		
	4	1		
	5 (lowest priority)	0		
Induction of labour is recommended for women who are known with certainty to have reached 41 weeks (>40 weeks + 7 days) of gestation. (Weak, low)	1 (highest priority)	5	7	30
	2	1		
	3	0		
	4	0		
	5 (lowest priority)	1		
If prostaglandins are not available, intravenous oxytocin alone should be used for induction of labour. Amniotomy alone is not recommended for induction of labour. (Weak, moderate)	1 (highest priority)	1	8	22
	2	1		
	3	3		
	4	1		
	5 (lowest priority)	2		

*Note: The number of respondents who identified a given recommendation within each of the five categories = n; the total number of respondents who selected the recommendation as one of their top five priorities = N Total; the calculated total score = Total Score. The total number of respondents (N Total) varies as it is dependent on the number of respondents who selected a given recommendation among their top five priorities.

Appendix I: Median score and interquartile range (IQR) for feasibility rankings by guideline recommendation

	Recommendation	Score [median (IQR 25th, 75th)]
Prevention and treatment of PPH	The use of uterotonics for the prevention of PPH during the third stage of labour is recommended for all births.	9 (9, 9)
	Oxytocin (10 IU, IV/IM) is the recommended uterotonic drug for the prevention of PPH.	8 (8,9)
	In settings where oxytocin is unavailable, the use of other injectable uterotonics (if appropriate ergometrine/methylergometrine or the fixed drug combination of oxytocin and ergometrine) or oral misoprostal (600 µg) is recommended.	8 (8,9)
	In settings where skilled birth attendants are not present and oxytocin is unavailable, the administration of misoprostol (600 ug PO) by community healthcare workers and lay health workers is recommended for the prevention of PPH.	8 (6,8)
	If bleeding does not stop in spite of treatment using uterotonics and other available conservative interventions (e.g., uterine massage, balloon tamponade), the use of surgical intervention is recommended.	8 (7,8)
Prevention and Treatment of Pre-eclampsia / eclampsia	Magnesium sulfate is recommended for the prevention of eclampsia in women with severe pre-eclampsia in preference to other anticonvulsants	8 (8,9)
	Magnesium sulfate is recommended for the treatment of women with eclampsia in preference to other anticonvulsants.	8 (8,9)
	The full intravenous or intramuscular magnesium sulfate regimens are recommended for the prevention and treatment of eclampsia.	8 (7,8)
	For settings where it is not possible to administer the full magnesium sulfate regimen, the use of magnesium sulfate loading dose followed by immediate transfer to a higher level healthcare facility is recommended for women with severe pre-eclampsia and eclampsia.	8 (7,9)
Induction of labour	Oral misoprostol (25 µg, 2-hourly) is recommended for induction of labour.	8 (6,8)
	Induction of labour is recommended for women with prelabour rupture of membranes at term.	8 (8, 9)
	Induction of labour is not recommended in women with an uncomplicated pregnancy at gestational age less than 41 weeks*.	8 (8,9)
Augmentation of labour	Active phase partograph with a four-hour action line is recommended for monitoring the progress of labour*.	8 (8,8)
	Encouraging the adoption of mobility and upright position during labour in women at low risk is recommended.	9 (9,9)
	Continuous companionship during labour is recommended for improving labour outcomes.	7 (7,8)
	The use of oral misoprostol for labour augmentation is not recommended*.	9 (8,9)

*Recommendation was re-rated due to disparate responses observed among participants. The median and IQR is provided for the re-rated rankings for these recommendations.
