# Supplementary material for: Barriers, Facilitators and Priorities for Implementation of WHO Maternal and Perinatal Health Guidelines in Four Lower-Income Countries: A GREAT Network Research Activity
Source: PLoS One. 2016 Nov 2;11(11):e0160020. doi: 10.1371/journal.pone.0160020 (PMC5091885; doi:10.1371/journal.pone.0160020)
Supplement: S3 File — (PDF) [file pone.0160020.s003.pdf]

Understanding Barriers and Facilitators to Implementation of Maternal Health Guidelines in Tanzania: A Great Network Research Activity

---

Final report on findings

Dar es Salaam, Tanzania

5 and 6 November 2014

Prepared by:

Sobia Khan[1], Caitlyn Timmings[1], Dr. Joshua Vogel[2], Shusmita Islam[1], Dr. Lisa Puchalski Ritchie[1,3], Dr. Dina Khan[2], , Dr. Julia E. Moore[1], Dr. A. Metin Gülmezoglu[2], Dr. Ahmad Makuwani [4], Dr. Ama Kasalanga [4], Dr. Godfrey Mbaruku[5], Dr. Mwifadhi Mrisho[5] and *Dr.Sharon E. Straus[1,3]*

[1]Knowledge Translation Program, Li Ka Shing Knowledge Institute, St. Michael's Hospital, Canada

[2]UNDP/UNFPA/UNICEF/WHO/World Bank Special Programme of Research, Development and Research Training in Human Reproduction (HRP), Department of Reproductive Health and Research, World Health Organization, Headquarters, Switzerland

[3]University of Toronto, Canada

[4] Ministry of Health and Social Welfare, Tanzania

[5] Ifakara Health Institute, Tanzania

Acknowledgements

We would like to thank the Ministry of Health Tanzania, Ifakara Health Institute, and PATH for graciously hosting us in Dar es Salaam, and would like to especially thank Drs. Makuwani, Kasangala, Mbaruku and Mrisho for their expertise and guidance throughout the process. We would also like to thank Amos Mugisha from PATH Tanzania for his support and assistance in planning the workshop and associated acitivites. We would like to acknowledge WHO, PATH and the United Nations (UN) Commission on Life Saving Commodities for funding the project activities. We also wish to acknowledge the GREAT Network for their strategic guidance in designing this activity. This activity is a part of a series of projects that the GREAT Network is involved with in partnership with low and middle income countries.

Contact

For questions about this report, please contact:

Sobia Khan, Research Coordinator
Knowledge Translation Program
Li Ka Shing Knowledge Institute
St. Michael's Hospital
Toronto, Canada
Email: khans@smh.ca
Phone: 416-864-6060 ext. 77522

Abbreviations

**CCHP**	Comprehensive Council Health Plan
**CHW**	Community Health Worker
**DL**	District level
**FGD**	Focus Group Discussion
**EmONC**	Emergency Obstetric and Newborn Care
**GREAT**	Guideline-driven, Research priorities, Evidence synthesis, Application of evidence, and Transfer of knowledge
**HRH**	Human Resources for Health
**IHI**	Ifakara Health Institute
**IP**	Interprofessional
**IQR**	Interquartile Range
**IV**	Intravenous
**KT**	Knowledge Translation
**LMIC**	Low and Middle Income Country
**NL**	National Level
**MDG**	Millennium Development Goal
**MMR**	Maternal Mortality Rate
**MNCH**	Maternal and Newborn Child Health
**MNH**	Maternal and Newborn Health
**MoHSW**	Ministry of Health and Social Welfare
**PPH**	Post-Partum Haemorrhage
**RCH**	Reproductive and Child Health
**TBA**	Traditional Birth Attendant
**SBA**	Skilled Birth Attendant
**SMH**	St. Michael's Hospital
**UN**	United Nations
**WHO**	World Health Organization

Executive Summary

Background

The World Health Organization (WHO, Switzerland), St. Michael's Hospital (Canada), Ministry of Health and Social Welfare (Tanzania), Ifakara Health Institute (Tanzania), and PATH (Tanzania) formed a collaboration to strategize the implementation of four key WHO maternal and perinatal guidelines:

1) Prevention and treatment of post-partum haemorrhage (PPH) (2012);
2) Prevention and treatment of pre-eclampsia and eclampsia (2011);
3) Induction of labour (2012); and
4) Augmentation of labour (2014).

An in-country workshop was held to determine key recommendations that will inform the development of the multi-level implementation strategy for improving use of the guidelines nationally. Recommendations resulted from:

1) identifying barriers and facilitators to the implementation of the four priority guidelines in Tanzania;
2) identifying the most important and feasible recommendations for implementation; and
3) providing suggestions for potential implementation strategies based on the barriers and facilitators identified, and the perceived feasibility of implementation.

The purpose of this report is to provide health care system stakeholders in Tanzania with key findings from pre-workshop and workshop activities and to inform future activities to optimize implementation of these guidelines.

Methods

Mixed methods were used to collect data on priorities, barriers, facilitators, and potential implementation strategies for the four identified guidelines in Tanzania. Primary data collection occurred during the in-country two-day workshop, this involved focus group discussions, ranking exercise and small and large group discussions. The exercise aimed to explore barriers and facilitators; identify guideline priority areas; and develop potential implementation strategies to fit the local context. Prior to the workshop, a survey was administered to inform workshop proceedings.

Findings

Fifteen stakeholders participated in the pre-workshop survey and twenty-eight stakeholders participated in the in-person workshop. Stakeholders represented multiple disciplines from diverse geographic regions and levels of the health care system including: health care administrators, policymakers, non-governmental organization

staff, representatives from professional associations, frontline health care providers (e.g., physicians, nurses, and midwives), and researchers/academics.

Findings from the focus group discussions described **issues at the level of the health care system,** which included factors related to policies and wider systemic conditions in Tanzania that can affect implementation of the WHO guideline recommendations. These factors included: *access to resources; continuity of care; monitoring and evaluation; policies; and dissemination of guidelines*. **Issues at the level of the health care provider** that may affect guideline implementation were prevalent, and included: *beliefs, attitudes, and buy-in about the use of guideline recommendations; knowledge and skills needed to implement the guidelines; as well as training, coaching, and professional development around guideline implementation.* Finally, **issues at the level of the patient/community** that may affect guideline implementation included: *health-seeking behavior and preferences for care; community champions; and socioeconomic status.*

The ranking exercise resulted in a participant-driven assessment of the feasibility of 12 guideline recommendations that were deemed to be priorities in Tanzania. Within subsequent small group discussions, multiple implementation strategies were suggested to overcome barriers.

Recommendations and conclusion

Key messages that emerged from the pre-workshop and workshop activities are as follows:

- Drug procurement, management, and distribution practices are not operating optimally. Key suggestions to improve drug ordering and monitoring across the country included accountability measures for timely request and reporting, and implementing cost-sharing programs. There is a need to ensure that oxytocin is stored at 2 – 8 degrees (Celsius) at all levels of handling to maintain the cold chain system. This may minimize “drug waste” (i.e., medications expiring or becoming unstable due to storage).
- Ensure access to equipment such as refrigerators for drug storage, and basic supplies such as gloves and blood pressure cuffs. Budgeting for essential equipment and supplies can be improved in a Comprehensive Council Health Plan to ensure adequate funding.
- Recruitment and retention strategies focusing on rural areas in particular should be strengthened to ensure adequate human resources for health. Cross-training of existing staff in maternal and perinatal health so that they can be re-distributed within and across facilities as needed may reduce the burden on overworked teams in all areas of Tanzania.
- Increased opportunities for training are essential to improve the implementation of guideline recommendations, with more focus on pre-service training and a refresher course for in-service training. The training should be competency- based, and should include continuing medical education, supportive supervision and mentorship programs.

- A focus on interprofessional training and promotion of a collaborative health care team model was cited as a key area for improvement that could improve attitudes, buy-in, and provider confidence in implementing a guideline recommendation.
- There is a need to create more formal linkages between the various levels of health care facilities to better coordinate and standardize maternal health care for the community. Opportunities to form linkages through technology (e.g., telemedicine) are currently being piloted in Tanzania.
- There is a need for wider dissemination of guidelines beyond distributing them directly to users. This could be achieved through strategies such as mass media campaigns, educational materials and community champions.

Many of the barriers, facilitators, and resultant implementation strategies identified regarding the four WHO maternal and perinatal guidelines are applicable to other priority areas in health care; therefore, these findings can inform and be integrated into future barrier and facilitator assessments and guideline implementation planning initiatives in Tanzania.

Table of Contents

Acknowledgements 2
Abbreviations 3
Executive Summary 4
Background 8
  Development of an International Partnership 8
  Purpose of report 9
Methods 10
  Participant recruitment 10
  Pre-workshop survey 10
  In-person workshop 10
    Focus groups 10
    Ranking exercise 11
    Small group discussions 11
  Analysis 11
  Triangulation of methods 12
Findings 13
  Pre-workshop survey 13
    Demographics 13
    Prioritizing guidelines 13
    Prioritizing recommendations within guidelines 14
  Focus groups 14
    Perceptions on the WHO guidelines and priority recommendations 15
    Factors affecting implementation of priority recommendations in the Tanzanian context: Identifying barriers and facilitators 16
  Ranking exercise 25
  Recommendations to inform a country-specific implementation plan 25
Limitations 28
Summary and conclusions 28
References 30
Appendix A: Pre-workshop survey 32
Appendix B: Focus group discussion guides 39
Appendix C: Pre-workshop survey findings for priority recommendations 40
Appendix D: Median score for feasibility rankings by guideline recommendation 43

Background

Despite a growing body of knowledge to support the use of evidence-based guidelines in clinical practice, health care systems worldwide are failing to use research evidence optimally to improve the quality of health care delivery[1]. Inadequate use of evidence in practice often results in inefficiencies, and reduced quantity and quality of life[1-6].

Low and middle-income countries (LMICs), including Tanzania, often face numerous challenges in applying research evidence[7]. According to the Ministry of Health and Social Welfare's (MoHSW) assessment of Maternal and Newborn Child Health (MNCH) program indicators related to the 2015 One Plan (National Road Map Strategic Plan to Accelerate Reduction of Maternal, Newborn and Child Deaths in Tanzania 2008-2015), approximately 7,900 women still die each year from complications of pregnancy and childbirth. Contributing factors include limited access to, and poor quality of, health services and emergency obstetric and newborn care (EmONC); poor referral systems; shortage of skilled human resources for health (HRH); and lack of appropriate infrastructure, essential equipment; and medicines. This assessment described gaps in the implementation of policies/guidelines that hinder access to and provision of quality MNCH services[8]. Recognizing these challenges faced by LMICs such as Tanzania, there is a growing interest in how knowledge translation (KT) approaches can be tailored and applied to the area of MNCH to improve implementation of evidence-based clinical practices.

The World Health Organization (WHO) has partnered with the KT Program based at St. Michael's Hospital (SMH) in Toronto, Canada to establish an international partnership called the GREAT (Guideline-driven, Research priorities, Evidence synthesis, Application of evidence, and Transfer of knowledge) Network, funded by the Canadian Institutes of Health Research. The GREAT Network uses a unique evidence-based KT approach to support LMICs in implementing evidence-based clinical practice guidelines that can reduce maternal morbidity and mortality. The GREAT Network brings together relevant health care stakeholders in LMICs to identify and assess the priorities, barriers, and facilitators to guideline implementation, and supports the efforts of stakeholders to develop a guideline implementation strategy tailored to the local context.

Development of an International Partnership

A partnership was established between the KT Program at SMH, WHO (Department of Reproductive Health and Research, Switzerland), PATH and health care system stakeholders of Tanzania to provide technical support to increase the uptake of evidence-based guidelines in Tanzania.

The objectives of this partnership include:

1. Providing key recommendations to inform the development of a multi-level implementation strategy for improving use of guidelines nationally;

2. Supporting local stakeholders in the development and delivery of the implementation strategy; and
3. Supporting local stakeholders in the development of a monitoring and evaluation plan to assess impact of guideline implementation.

An in-country workshop, funded by WHO, PATH and the UN Commission on Life-Saving Commodities, was the initial activity conducted as part of this partnership. Informed by consultations with Ifakara Health Institute (IHI), PATH, WHO, and the MoHSW the following four WHO guidelines focused on maternal and newborn health (MNH) were selected as key priorities for the in-country workshop and related implementation activities:

- Prevention and treatment of post-partum haemorrhage (PPH) (2012)[9];
- Prevention and Treatment of pre-eclampsia and eclampsia (2011)[10];
- Induction of labour (2012)[11]; and
- Augmentation of labour (2014)[12].

Purpose of report

This report provides key findings from the in-country workshop held in November 2014. The aim of the workshop was to meet the first objective of the international partnership – to provide key recommendations to inform the development of a multi-level implementation strategy for improving use of the selected WHO guidelines in Tanzania. The selected WHO MNH guidelines and key recommendations described in this report directly align with Tanzania's 2013 Implementation Plan for Life-Saving Commodities for Women and Children[13].

Methods

Mixed methods were used to collect data including:

1) a pre-workshop survey;
2) in-workshop focus groups and small group discussions; and
3) an in-workshop ranking exercise.

These methods are briefly outlined below.

Participant recruitment

Participants were identified in consultation with MoHSW, IHI, PATH, and the WHO. To ensure representation from across the health care system, individuals with roles as health care administrators, policymakers, non-governmental organization staff, representatives from professional associations (e.g., Tanzania Midwives' Association), frontline health care providers (e.g., physicians, nurses and midwives), and researcher/academics were identified. Individuals representing different levels of the health care system were also identified to ensure representation from health facilities, as well as district, regional and referral hospitals. Geography was a key consideration in participant selection to ensure representation of stakeholders from both rural and urban centres across the country.

Pre-workshop survey

The pre-workshop survey was designed to provide a preliminary understanding of key priorities related to the selected WHO MNH guidelines in the Tanzanian context. Surveys were administered between October and November 2014. The survey [Appendix A] was electronically disseminated to a wide variety of stakeholders by e-mail invitation, and was available for completion on a web-based platform or a paper-based format. Consent was implied by completion of the survey.

In-person workshop

A sample of survey respondents and additional participants who represented the stakeholder groups of interest (described above) were invited to participate in a two-day in-person workshop in Dar es Salaam, Tanzania. At the workshop, stakeholders participated in focus group discussion (FGDs) on Day One, and in a ranking exercise and small group discussion on Day Two.

Focus groups

Participants were divided into four FGDs, each composed of six to eight participants. FGDs were organized according to role and/or level of the health care system:

1) clinicians (including physician, nurses and midwives);
2) district level (DL) MoHSW (including district and regional medical officers);
3) national level (NL) MoHSW (including national level program managers and departmental directors);

4) interprofessional (IP) group (including researchers/academics, non-governmental organizations, and international organizations).

FGD sessions lasted approximately 90 minutes and were conducted in English using a semi-structured discussion guide [appendix B]. The FGDs centered on identifying priority recommendations based on importance as well as barriers and facilitators to implementing these recommendations in the Tanzanian context.

Ranking exercise

A shortlist of recommendations was generated based on selections made in the **Day One** FGDs and following deliberations among facilitators and local experts. On **Day Two**, workshop facilitators engaged participants in a nominal group process[14] to rate the feasibility of implementing each of the identified guideline recommendations. Consistent with the RAND Appropriateness Method[15], participants individually ranked each recommendation, using a 9-point Likert scale (where 1= extremely not feasible and 9= extremely feasible). When responses were highly disparate, large group discussion took place and responses were re-ranked with the aim of reaching a higher level of agreement.

Small group discussions

Following the ranking exercise, small group breakout discussions were conducted by facilitators. Participants were guided in an exercise to map implementation barriers to the priority recommendations, followed by an exercise to identify context appropriate implementation strategies that could address identified barriers.

Analysis

Pre-workshop survey data were analyzed using descriptive statistics. For Section 2 (Guideline prioritization), a numerical count was used to depict the ranking of the four guidelines. For Section 3 (Prioritization of recommendations), data were recoded so that the highest ranking received the highest score (e.g., 1st ranked priority= assigned score of 4). The Total Score was then calculated to account for both the assigned rank and the number of times the guideline or recommendation was identified as one of the top five priorities.

FGDs and small group discussions were digitally recorded and detailed notes were taken to supplement recordings. After familiarization of the data from the recordings and notes, data were qualitatively analyzed by an expert analyst at SMH who was also involved with the workshop, using a thematic analysis approach.[16] Themes were developed in consultation with meeting facilitators to discuss interpretations of the data for a shared understanding of key findings.

Results from the individual ranking exercise were analyzed using descriptive statistics [median, interquartile range (IQR) including the score for the 25th percentile and 75th percentile] of participant assigned feasibility

ratings for each of the identified recommendations. Small group discussions were analyzed using the same method as described for FGD sessions above.

Triangulation of methods

Using the technique of integration, data collected across all methodologies were considered in detail to draw meaningful and pertinent recommendations that are feasible and relevant for the Tanzanian context.

Findings

Pre-workshop survey

Survey findings are presented below according to: 1) priorities between guidelines; and 2) priorities within guidelines (i.e., priority recommendations).

Demographics

A total of 15 stakeholders participated in the pre-workshop survey. Survey respondents represented six different regions across Tanzania with Dar es Salaam the most highly represented region (50%). The survey respondents also varied in terms of role and the level of the health care system in which they were situated.

Prioritizing guidelines

The majority of respondents (n=11) selected the prevention and treatment of PPH guideline as the highest priority in Tanzania (**Table 1**). The prevention and treatment of pre-eclampsia and eclampsia (n=10), induction of labour (n=8), and augmentation of labour (n= 9) guidelines were ranked as the second, third and fourth highest priorities, respectively.

Table 1. WHO maternal and perinatal guidelines ranked in order of importance by pre-workshop survey respondents

Guideline	Priority	n (N=15)	%
Prevention and treatment of PPH	1 (highest priority)	11	73
	2	4	27
	3	0	0
	4 (lowest priority)	0	0
Prevention and treatment pre-eclampsia and eclampsia	1 (highest priority)	4	27
	2	10	67
	3	1	7
	4 (lowest priority)	0	0
Induction of labour	1 (highest priority)	0	0
	2	1	7
	3	8	53
	4 (lowest priority)	6	40
Augmentation of labour	1 (highest priority)	0	0
	2	0	0
	3	6	40
	4 (lowest priority)	9	60

Prioritizing recommendations within guidelines

Using a scale of 1 to 5 (where 1=highest priority and 5=lowest priority), survey respondents identified the five most important recommendations within each of the four guidelines and ranked them in order of importance for implementation in Tanzania. Detailed results are presented in [Appendix C].

A descriptive analysis of the survey identified key clinical areas that emerged in relation to recommendations for each guideline (**Table 2**). Due to the low number of completed survey responses received, a meaningful analysis of the rankings could not be conducted.

Table 2. Clinical areas of interest identified in relation to each of the four WHO guidelines

Guideline	Clinical area of interest identified
Prevention and treatment of PPH	• *Use of uterotonics*<br>• *Use of controlled cord traction (CCT)*
Prevention and treatment of pre-eclampsia and eclampsia	• *Calcium supplementation*<br>• *Use of magnesium sulfate*<br>• *Use of acetylsalicylic acid*<br>• *Use of antihypertensive drugs*
Induction of labour	• *Induction of labour for women with premature/prelabour rupture of membranes*<br>• *Use of vaginal prostaglandins*
Augmentation of labour	• *Use of active phase partograph*<br>• *Digital vaginal examination*<br>• *Continuous companionship*<br>• *Adoption of mobility*

Focus Group Discussions

A total of 32 stakeholders participated in the in-country workshop across both days. Key discussion points have been synthesized across FGDs and are organized into perceptions on the WHO guidelines and priority recommendations as well as factors that influence implementation of priority guideline recommendations. For an overview of the major themes and sub-themes identified at each level, refer to Figure 1 below.

Figure 1. Overview of FGD findings on perceptions on the WHO guidelines and barriers and facilitators to guideline implementation

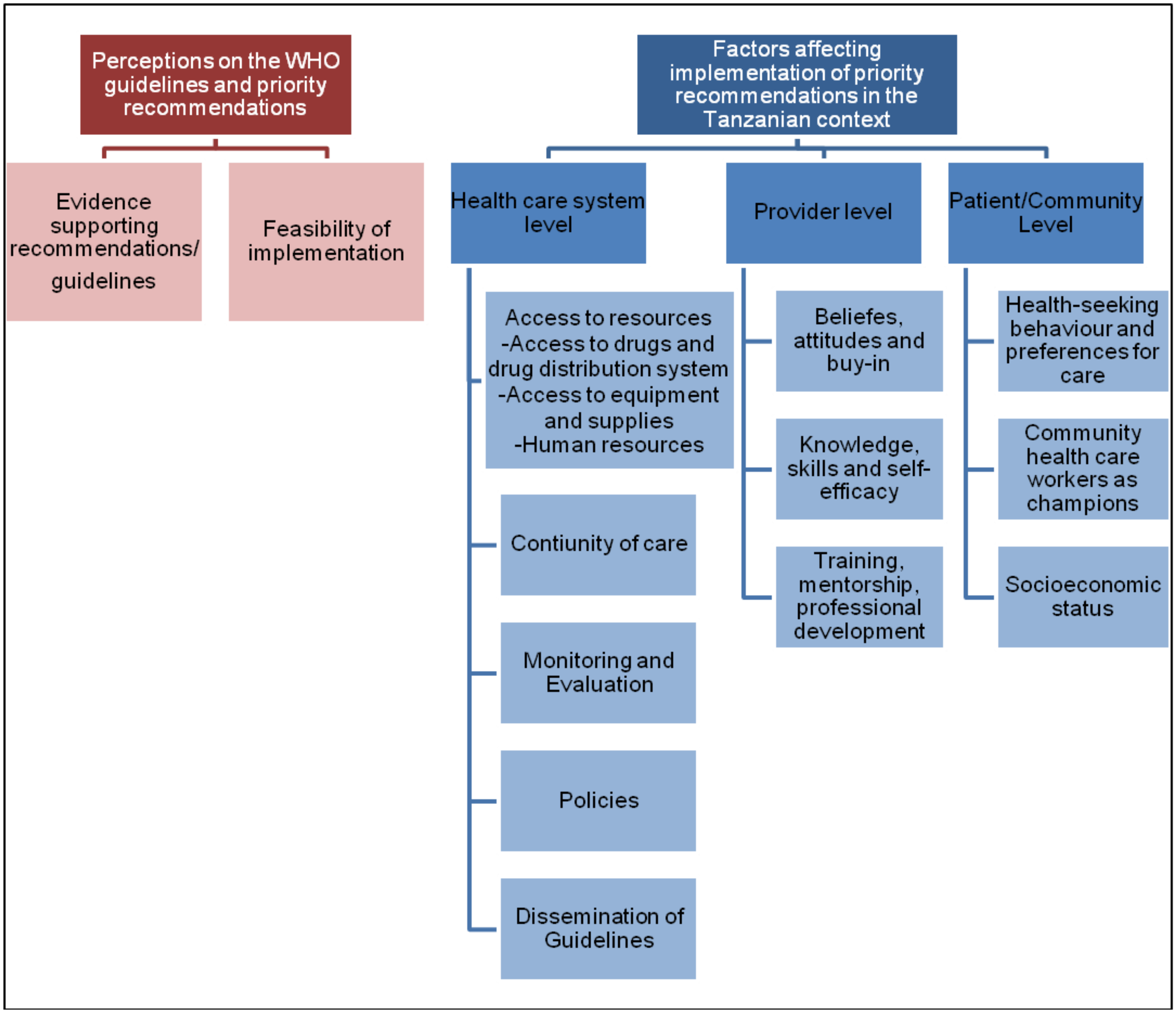

Perceptions on the WHO guidelines and priority recommendations

FGD participants discussed their perceptions on the characteristics of the guidelines and recommendations. It is important to understand these perceptions, as agreement and buy-in for the recommendations are pre-requisites for successful implementation.

Evidence supporting recommendations/guidelines

Overall, participants from all FGDs were interested in discussing the evidence that supported the guideline recommendations and in learning that sufficient research evidence exists to support the recommendations. As well, clinician FGD participants were keen to hear about the experiences of their peers who had implemented the recommendations; in some cases, FGD participants could be convinced to choose a recommendation as a

priority area based on the anecdotal evidence described by their clinician peers. There was also general discussion about the willingness to adopt WHO guidelines, where FGD members generally felt that recommendations with strong global evidence could be used as a basis for national guidelines. However, participants in the clinician FGD felt that there was a need for additional statements in the guideline recommendations to make them more specific, which would enhance the practice of the guidelines (e.g., adding in a statement about ruling out obstruction before administering a uterotonic).

Feasibility of implementation

Overall, FGD participants noted that recommendations perceived as easy to practice were the most acceptable, and therefore more amenable to broad implementation. "Easiness" was defined as having the necessary resources secured (e.g., recommendations related to uterotonic use) or as not requiring any additional resources (e.g., positioning during labour). Recommendations that were not as feasible to implement were those that would require a policy or cultural (i.e., work and/or community culture) shift, or those that would require improved or additional resources/facilities/materials (e.g., companionship during labour, which would necessitate private rooms). Participants' definitions of feasibility were considered when ranking the feasibility of the individual guideline recommendations (see results on "Ranking Exercise").

Factors affecting implementation of priority recommendations in the Tanzanian context: Identifying barriers and facilitators

FGD participants discussed factors that influence implementation of priority guideline recommendations and operate at the level of: (1) the health care system; (2) the provider; and (3) the patient/community.

Health care system level

Various factors were identified to operate at the level of the health care system or to be a product of systemic conditions in Tanzania that can affect the implementation of the WHO guideline recommendations. These factors include: access to resources, equipment and supplies; continuity of care; monitoring and evaluation; policies; and dissemination of guidelines.

Access to Resources

> *"Postpartum hemorrhage of course has been addressed, but the main problem is availability of equipment and drugs; that's the main problem on postpartum hemorrhage". – DL FGD Participant*

A common theme that emerged across all FGDs was that limited access to resources in health care facilities makes it difficult for health care providers to implement guideline recommendations. Participants discussed

how lack of access to drugs, equipment, supplies and adequate HRH directly impede the implementation of guideline recommendations, particularly in lower level facilities and rural areas.

Access to drugs and the drug distribution system

Limited access to drugs and challenges in the drug distribution system were identified as barriers to the successful implementation of recommendations pertaining to uterotonic drugs. The clinician FGD discussed that the medical supplies department has a well-defined system of drug distribution, but not all facilities receive adequate supplies. Uterotonic drugs such as oxytocin, misoprostol and ergometrine are unavailable in some facilities, especially rural facilities, due to stock-out and inefficiencies in the drug ordering system. In the NL FGD, lack of collaboration within the health system was identified as compounding these inefficiencies, especially in cases where the person responsible for ordering drugs may not understand the needs of frontline workers in the area of maternal health care. Due to budget considerations, it may not be feasible for facilities to ensure that first, second and third line uterotonics (oxytocin, ergometrine and misoprostol, respectively) are stocked.

The NL FGD suggested that if a facility ensures that a consistent supply of oxytocin is maintained, they may choose not to stock ergometrine or misoprostol (which may expire before they are actually needed) and instead allocate the money towards purchasing other necessary supplies.

Similar to the case of uterotonic drugs, limited availability of injection magnesium sulfate in rural health facilities and medical stores was identified by both DL and IP FGDs as a barrier to implementing recommendations pertaining to the prevention and treatment of preeclampsia/eclampsia.

Access to equipment/supplies

In addition to availability, appropriate storage of uterotonic drugs was a concern identified by the NL FGD, particularly in the case of oxytocin. Participants expressed uncertainty over the appropriate storage method to be used at lower level facilities, such as whether oxytocin should be refrigerated, the ideal temperature for refrigeration and whether it could be stored in vaccine refrigerators or if additional refrigerators would be required for adequate storage.

Lack of gloves, syringes, blood pressure cuffs, anti-shock garments, clean water and saline were also discussed. Limited availability of blood pressure machines at health facilities was identified as a potential barrier to implementing pre-eclampsia/eclampsia recommendations. Similarly, limited availability of ultrasound machines to determine gestation age at lower level facilities was identified by the NL FGD as a barrier to induction of labour guideline recommendations.

Human Resources for Health (HRH)

"How will you see the patient when there is not one but there are 60 women lying there and all are in second stage? How will you see that one mother?" –DL FGD Participant

A shortage of health care providers was described as a barrier experienced throughout the health care system in Tanzania. Due to the large number of deliveries of newborns per day in each facility (5- 10 deliveries per day in most hospitals and upwards of 80-120 deliveries per day in very large hospitals as stated by one district level facility), a number of key challenges were identified as affecting implementation of WHO guidelines. These include:

- Health care providers not being able to remember or not having the time to administer uterotonic drugs at the appropriate time;
- Health care providers not being able to adequately monitor pregnant women for complications after administering uterotonic drugs, and;
- Physicians not being available to conduct surgical interventions if complications arise.

A participant from the DL FGD explained that lack of time and capacity required for a health care provider to dedicate their attention to one pregnant woman may lead them to avoid induction or augmentation of labour.

Continuity of Care

FGD participants described multiple factors related to the transfer of mothers to higher level facilities and of post-natal care that may affect the implementation of guidelines. First, FGD participants noted that transfer of patient when complications are experienced during delivery (e.g. pre-eclampsia/eclampsia) can be difficult as a result of numerous systems-level challenges:

"Another barrier is our referral system. We might have a good system but the problem is implementing or using the system is difficult because of our geographical setup and the roughness of our roads. Even if we have very good system but how to refer is another very big challenge because you may find that between health facilities there are maybe 250 kilometers and we don't have enough ambulances." – IP FGD Participant*

Participants within the IP FGD who worked at the community level also discussed the state of post-natal follow-up, where the problem is two-fold: appointments are typically made for the baby and no follow up of the mother after delivery; and even when appointments are made, less than half of the women attend follow-up appointments. Participants provided examples of women walking home a long distance after delivery, and perhaps not willing to attend post-natal care appointments due to the lack and cost of transport and distance to access care. Similarly, these factors create barriers for health care workers to provide necessary services,

as skilled birth attendants (SBAs) who live far from health care facilities experience difficulties travelling to facilities at night and often there is no electricity.

Monitoring and Evaluation

FGD participants described three key priority areas that can be either improved and/or leveraged as facilitators to evaluate guideline implementation efforts:

1) Monitoring the movement of drugs through the distribution system to assess availability and stock-outs. Tracer drugs are being used in some parts of the country and could be leveraged to determine stocks of drugs pertaining to the selected guideline priority areas to ensure that stocks are available equally across districts.

**Note that most health facilities are located 50 kilometers or less from one another*

2) Information about births is collected and maintained inconsistently. Currently, some facilities are utilizing paper records, while others are maintaining large electronic databases. Implementing similar prospective data collection and storage mechanisms nationally could enhance the quality of data collected at the country level.
3) Methods of evaluation should include development of indicators for guidelines and assessing these indicators on a regular basis. FGD participants described excellent data collection on existing indicators at the level of the front-line clinicians, however use of the data for the purpose of monitoring and evaluation is lacking.

Policies

Participants indicated that despite the presence of good political will towards guideline implementation, there are opportunities for policies to be changed to better facilitate implementation of certain priority recommendations. Overall, these policy changes would help support an implementation mechanism for guidelines (when the implementation plan is developed and established). An example provided within the IP FGD involved reviewing policies on approving the use of misoprostol by community health workers (CHWs). Another discussion focused on how the government's plan to build health centres in each village would facilitate implementation of policies advocating for women to use health facilities for child birth. Clinicians mentioned that the health care system and funding policies should place more focus on facilitating guideline implementation.

Dissemination of Guidelines

Another barrier to implementation featured across the FGDs was lack of awareness of the existence of guidelines by health care workers. Participants perceived that more effective mechanisms may be necessary to better disseminate information to frontline health care workers so that they understand and are familiar with

current best practices. In some cases, although frontline workers may be aware of guidelines they may not use them because they lack time to enhance their understanding of the guidelines or to receive guidance on how to apply the recommendation in their practice. Another problem identified was that it may be difficult for frontline health care workers to stay up to date with current recommendations and make informed decisions.

> *“We have these guidelines but when that is disseminated to the lower levels it's also another problem that people who are working at the grassroots, at the lower level, …they get these guidelines late when they are about to be reviewed again" - NL FGD participant*

Provider level

Factors affecting the implementation of priority WHO guideline recommendations identified at the provider level, include: beliefs, attitudes and buy-in; knowledge, skills and self-efficacy; and training, coaching, and professional development.

Beliefs, attitudes, and buy-in

FGDs identified the attitudes, beliefs, and buy-in of health care providers as factors that can either facilitate or hinder implementation of guideline recommendations in practice. Two FGDs (clinician and DL) identified resistance to change as a provider level barrier to implementing guideline recommendations. Resistance may stem from beliefs about the consequences of practicing a recommendation. Guideline recommendations that centre on medication delivery and dosing were generally associated with potential fear of harms to the pregnant women, either because of a fear of the drug’s strength, lack of confidence in the capability of some health care providers (e.g., nurses, midwives) to administer the appropriate dose of the drug, or the inability of some health care providers in busy facilities to monitor the women after drug administration. There is also a fear that misoprostol can be misused for abortion:

> *“The issue is the misoprostol intended for PPH, can it be misused for abortion. That’s where we don’t have evidence it is just an argument. No one has got evidence because no one has ever conducted a study in Tanzania to see how many women, who are inducing abortion, are using a miso[prostol] which was intended for PPH management or prevention. So since we don’t have that evidence, we cannot even produce it here. It is just a fear that is there, it is a genuine fear which we cannot activate because we don’t have any other facts...” – IP FGD Participant*

Concerns over infection prevention and control measures may interfere with certain practices such as the use of anti-shock garments as a temporary measure for managing PPH and amniotomy in the induction or augmentation of labour due to concerns of the risk of contamination if not adequate cleaned/sterilized.

One facilitator for improving guideline implementation identified across two FGDs (clinician and DL) was strong leadership or supervision (described by participants as “champions for change”) that could motivate frontline clinicians to incorporate guideline recommendations into their practice. Participants described an existing national supervision system of health care facilities, centralized and conducted by the MoHSW, that could be leveraged to enforce change in areas where it is working well currently and strengthened in some regions where supportive supervision is infrequent. Another facilitator described by the clinician and DL FGDs was using rewards as incentives to enhance buy-in of the guideline recommendations. For clinicians, acknowledgment of staff’s good work by leadership was considered a motivating factor for implementing a guideline recommendation. One participant in the DL FGD described a monetary incentives system that is currently being piloted in two districts and could be rolled out nationally. In this system, facilities that meet targets for clinical practice, including the appropriate use of guidelines, are given bonuses. The participant reported that this approach was found to have increased the use of multiple guidelines (including some MNH guidelines) by almost 70%. This example suggests that a monetary incentives system may facilitate guideline implementation, although sustainability of this type of system was not discussed.

Knowledge, skills and self-efficacy

> *"You don’t wait until someone has PPH for you to remember your skills. Because it’s just a matter of a few minutes and either you lose a mother or you save the mother." - DL FGD Participant*

Ashortage of health care providers to mitigate extensive workloads and lack of knowledge or skills of some health care providers, especially in rural areas, were identified as barriers to providing evidence-based MNH in Tanzania. It was mentioned in the clinician FGD that health care providers might know about guideline recommendations but not be equipped with the skills and competencies to perform certain recommended tasks.

While discussing self-efficacy (i.e., self-confidence in the capability to perform a task or to change a behaviour) for the prevention and management of PPH, some participants within the clinician FGD expressed confidence in performing recommended practices while others stated that they could benefit from additional training, specifically in relation to using uterotonics and using uterine massage for the prevention and treatment of PPH.

In the DL FGD, it was perceived that knowledge of PPH guideline recommendations among health care providers is higher than that of pre-eclampsia and eclampsia recommendations. Lack of knowledge and skills were cited as factors that may prohibit providers from administering injection magnesium sulfate due to limited understanding of the appropriate dosage and method of administration. As injection magnesium

sulfate has not been traditionally used to treat pre-eclampsia/eclampsia, participants described challenges related to its use:

> *"Another hindering factor in probably the use of guidelines among these clinicians is probably they feel they know. They feel they have been practicing this for years and years. They have been treating eclampsia without magnesium sulfate and they know that the guideline is there in the drawer probably and they feel like they are busy. They have cured patients so probably they feel like they don't have time to take out the guideline and read." - DL FGD Participant*

Participants discussed that doctors working on the labour ward may attend seminars to learn how to administer loading doses, but this knowledge is often not shared with the rest of the facility staff. The issue of possible misuse of injection magnesium sulfate was also identified, as some clinicians may prescribe it before fully examining the patient, resulting in complications such as renal function problems.

Training, mentorship, professional development

The need for continuous professional development for health care providers in the form of continuous medical education, on the job training, refresher courses and mentorship was identified in all FGDs as a requirement to ensure retention of skills and sustain adherence to recommendations. In the NL FGD, participants discussed how inadequacies in the training curriculum may contribute to lack of compliance with guideline recommendations and that the curriculum should be improved to include specific training on how to interpret, adapt and use guidelines. In two of the FGDs (DL and IP), participants described how the modality of training should be changed to include more practical, hands-on training, especially for new graduates. The need for mentorship was also identified:

> *"We put people in the class but then after that there is nothing going on. Just giving them guidelines, just giving them the slides and knowledge, doesn't change them much. So I think we have to change the modality of our training. We can start with class type training but I think that now we need to move to the mentorship or mentoring our health workers." - DL FGD Participant*

A number of participants in the IP FGD mentioned the benefits of creating simulations and promoting the use and availability of mannequins for health workers to practice how to manage PPH in emergency situations.

In addition to improving curriculum and access to training overall, specific areas were identified as training priorities:

- Training SBAs to administer oxytocin;
- Training staff in lower/dispensary levels to use misoprostol and adapt policy accordingly;

- Training staff in lower/dispensary level to use manual compression and anti-shock garments for the prevention of PPH;
- Training health care providers across health centres/facilities nationally to ensure that many individuals are trained to perform surgical interventions such as caesarean sections; and
- Training dispensary level medical attendants and midwives how to manage EmONC.

Training priorities specific to management of pre-eclampsia/eclampsia included:

- Training dispensary level medical attendants and midwives to quickly identify a woman with the signs of eclampsia and start initial management while referring to higher facilities without delay; and
- Training clinicians on the choice and the route of administration of drugs for severe hypertension during pregnancy.

Finally, when training is provided, it was mentioned that challenges exist in terms of feedback or knowledge exchange; those who attend trainings do not routinely share their training experience and knowledge with colleagues in their health care settings where the recommendations are supposed to be implemented.

Patient/community level

When considering how the patient/community can influence implementation of the WHO guideline recommendations, factors in the following categories were described: health-seeking behavior and preference for care, community champions and socioeconomic status.

Health-seeking behavior and preference for care

Health-seeking behaviours of pregnant women can either facilitate or hinder implementation of guideline recommendations. Clinicians discussed that pregnant women may not seek care or comply with instructions to deliver in health facilities, given that 50% of pregnant women prefer to deliver their children at home in Tanzania. In two FGDs (NL and DL), participants mentioned that pregnant women may not comply with recommendations to deliver at higher level facilities but instead arrive at dispensary level facilities when they are already experiencing labour complications:

> *"On the patient side, they come to the health facility when the condition is already bad. So they come there and think, 'Okay, I'll just go to the hospital,' but eclampsia has been there for three days and now they come to the hospital, so the patient side is a problem. It's a barrier." - DL FGD Participant*

Raising community awareness on the risks of PPH was identified as a potential strategy to encourage pregnant women to go to higher level facilities earlier. Limited health-seeking behaviour was also

identified as a barrier to women at risk of preeclampsia or eclampsia as women with hypertension may not make follow-up visits to clinics to continue to monitor their blood pressure after delivery. As described earlier, in the IP FGD, Tanzania was identified as a country with very low post-natal checkup rates.

Preferences of pregnant women may also affect the implementation of guideline recommendations in relation to drug usage and administration. In the clinician FGD, a barrier to PPH was identified as some women refuse needle administration of oxytocin. In these cases oral administration of misoprostol may be used instead. In the IP FGD, however, it was mentioned that some women may not want to take oral medication while in labour, and in these cases, misoprostol may need to be administered vaginally. Therefore, guideline recommendations pertaining to both oral and vaginal administration of misoprostol should be known by clinicians and implemented based on patient preferences.

When discussing the use of anti-shock garments, some participants in the IP FGD voiced concern that clinicians would not apply the garment due to the perception that patients would not want to wear a device that has been re-used. A concern for privacy was identified as a potential barrier to companionship during labour.

Community Health Workers (CHWs) and Traditional Birth Attendants (TBAs) as Champions

> *"Community healthcare workers are the ones who will tell these women; pregnant women, to come and deliver at the facility, but then you have your chance of giving the oxytocin in the first minute of birth." - Clinician FGD Participant*

Participants in the clinician FGD explained that CHWs and TBAs are well known to pregnant women and efforts should be made to educate CHWs in early identification of potential labour complications and communication of appropriate information to pregnant women so they seek appropriate and timely care.

Socioeconomic status

A pervasive barrier that was identified as a determinant of health-seeking behaviour at the patient/community level was the socioeconomic status of the pregnant woman. Lack of economic resources can deter or delay a woman from seeking health care, contributing to maternal morbidity and mortality rates in Tanzania. For example, women with financial constraints are perceived to be less likely to comply with a health care provider’s recommendation to transfer to a referral hospital due to lack of access or inability to afford transportation.

Ranking exercise

Twelve recommendations across the four guidelines were selected as priority areas by FGD participants on Day One of the in-person workshop. Results of the Day Two ranking exercise are presented in Appendix D; in this exercise participants ranked the feasibility of recommendations (i.e., the extent to which it is feasible to implement a given recommendation in the Tanzanian context) on a scale from 1 to 9, with 1 being not feasible and 9 being very feasible to implement. The following five recommendations were deemed to be most feasible to implement in the Tanzanian context, with a median score of 9 (“extremely feasible”):

- In settings where oxytocin is unavailable, the use of other injectable uterotonics (ergometrine/ methylergometrine) or oral misoprostol is recommended. (Prevention and treatment of PPH guideline).
- The use of uterotonics for the prevention of PPH during the third stage of labour is recommended for all births. (Prevention and treatment of PPH guideline).
- Injection magnesium sulfate is recommended for the prevention of eclampsia in women with severe pre-eclampsia in preference to other anticonvulsants. (Prevention and treatment of Pre-eclampsia/eclampsia guideline).
- Injection magnesium sulfate is recommended for the treatment of women with eclampsia in preference to other anticonvulsants. (Prevention and treatment of Pre-eclampsia/eclampsia guideline).
- Active phase partograph with a four-hour action line is recommended for monitoring the progress of labour. (Augmentation of labour guideline).

Recommendations to inform a country-specific implementation plan

On Day 2, small group participants considered implementation strategies and activities that could address barriers to implementation of the twelve priority recommendations selected in the ranking exercise. Each small group focused on implementation strategies that were specific to the recommendations assigned to their group for discussion, and described recommendations that were broadly applicable across multiple guideline areas. A summary of the implementation strategies identified by workshop participants is presented in **Table 3.**

Table 3. Recommended strategies/activities to address perceived barriers to practicing guideline recommendations

Level of barrier	Category of barrier	Recommended implementation strategies/activities
Health care system level	Access to drugs and the drug distribution system	• Maintain a cold chain of oxytocin storage at all levels, were oxytocin is kept between 2 and 8 degrees (Celsius) to prevent degradation of the drug and avoid future drug waste.<br>• Require that request and reporting of drugs is completed on time to minimize stock-outs.<br>• If stock-outs occur, implement a cost-sharing program (facility absorbs partial cost of drugs to buy locally from private vendors until stock is replenished)
	Access to equipment/supplies	• Ensure that facilities have refrigerators to store oxytocin, or change facility policies to ensure that refrigerators used to store vaccines can also accommodate drugs like oxytocin. A suggested alternative was keeping cool boxes to store oxytocin.<br>• With a shift to patient-centred receipt of funding, facilities will need to be built to attract more patients and therefore should be conducive to the patient needs. This can be a facilitator for building labour wards that keep the woman's right to privacy while allowing space for companionship during labour in mind, and for providing better health services<br>• Budget all essentials in a Comprehensive Council Health Plan (CCHP) to govern procurement, supervision and training
	Human resources for health	• Re-distribute health care workers internally (within institutions) and externally (across institutions) to concentrate on maternal and newborn health services. To do this, all health care workers should be sufficiently trained to work in different clinical areas when required.<br>• Promote teamwork and shared responsibilities of health care staff within institutions. All facility staff should be able to work in the labour ward if needed and should be trained to do so<br>• Incentivize overtime with higher pay for overtime work<br>• Create competitive salaries and modern infrastructure (electricity, housing) in rural/remote communities to incentivize health care workers to work in underrepresented areas<br>• Support the current initiative at the national level to create more health care providers, but emphasize the quality of health care providers through better training and supervision, and potentially lengthened nursing programs (increase from 2 years to 3 – 4 years)
	Continuity of care	• Promote linkage of services between facilities. Women can be linked to clinical postnatal care by medical officers.<br>• Consider using telemedicine as a tool to link lower level and higher level facilities.<br>• Provide adequate transport services for transfer of women between facilities.
	Monitoring and evaluation	• Develop clinical indicators that will enable systematic and standardized monitoring of conditions and clinical practice (e.g., number of women receiving uterotonics)<br>• Use tracer drugs to monitor stocks of essential drugs (e.g., oxytocin, magnesium sulfate)<br>• Implement national logistics systems (already piloted in two districts) to monitor established indicators and drug distribution
	Policies/ political context	• Incorporate into policies an increased role for nurses and midwives in terms of consulting and decision making for patient care
Provider	Beliefs, attitudes, and buy-in	• Engage stakeholders (professional associations, front line clinicians) prior to the rollout of the guideline, to enable them to comment on the guideline prior to dissemination<br>• Create an organizational culture that promotes and supports accountability to professional standards and guidelines.<br>• Promote interprofessional collaboration and teamwork, so that all professionals support one another and are recognized

Level of barrier	Category of barrier	Recommended implementation strategies/activities
		for good work<br>• Implement reward systems within facilities, to increase motivation and buy-in and provide incentives for practicing the guideline recommendations. Rewards include implementing a“star” system, or ensuring the leaders vocally praise practices that meet or exceed expectations.<br>• Establish mentoring programs between more experienced and less experienced health care workers.<br>• Implement more supportive supervision at the facility level, and cascade supervision within districts (i.e., allowing health care providers to make short visits to hospitals or dispensaries to be mentored by staff in a different setting)<br>• Develop a supervisor checklist at the Department of Reproductive and Child Health (RCH) level, to be used by RCH supervisors who visit facilities so that supervision can be standardized and that practices requiring supervision are not missed<br>• Sensitize senior management to the issues faced by front line workers through enhanced communication, so that they are aware of issues and are more willing to constructively support staff
	Knowledge, skills and self- efficacy	• Develop protocols based on the guidelines at higher levels (Ministry/regional/district) and distribute to facilities for on-site guidance. Protocols should be user-friendly, ready-to-use, and visible (e.g., posted on wards) to act as reminders for health care workers.<br>• Provide guideline-relevant training (see below) to nursing and midwives, and adapt policies to empower them to approach physicians when a woman’s health is at risk and to be part of the decision making process
	Training, coaching, and professional development	• Strengthen orientation (pre-service), in-service training and professional development (e.g., continuous medical education) opportunities for all professions, including nurses and midwives. Training should have a hands-on component to help prepare workers for situations that they may encounter infrequently in their practice.<br>• Promote interprofessional training, to enhance collaboration and role recognition across the health care team.Scale up the practice of placing health care workers on a “probation” period after pre-service training that enables supervisors to assess competencies and skills prior to sending staff to other facilities for work. Probation should be a 2 – 3 week period.<br>• Train health care workers on appropriate, effective, and transparent communication with pregnant women, and on the rights of the patients so that women do not feel ill-treated when coming to the labour ward<br>• Ensure that school curricula for health care providers are updated to include the most recent guidelines.
Patient/ Community	Health-seeking behavior and patient preferences for care	• Advocate for the importance of the role of nurses and midwives, so that pregnant women feel comfortable in their care and will not blame these health care providers for negative outcomes<br>• Educate pregnant women on aspects of their care during labour (e.g., positioning and companionship), to help increase patient knowledge and subsequent buy-in for practices<br>• Consider scale up telephone reminders program to encourage women to attend antenatal care<br>• Use mass media to promote awareness of the benefits of the recommendations (e.g., companionship during labour)
	Socioeconomic status	• Tanzania has already extended free health care to pregnant women. Additional supports may include integrating cost-sharing for supplies and transport within the CCHP budget.

*Note that *Dissemination of Guidelines* and *Community Champions* were removed from this list as they were seen as facilitators rather than barriers

Limitations

There are three main limitations to this research. First, data were collected from a small sample of participants that may not be representative of the entire population working in the MNCH sector of Tanzania. Steps were taken, however, to ensure the sample was diverse and included representation from all or most major stakeholder groups and from multiple regions across the country. It should be noted that no patients or community members were included in any of the data collection activities. The pre-workshop survey response rate was not optimal and did not yield the level of response anticipated. Second, time, resource, and space restrictions were faced by project organizers in conducting this activity; therefore, a purposeful convenience sample was used to identify stakeholders to participate in the pre-workshop survey and in-person workshop. Finally, cultural barriers and local contextual factors may have prevented a more robust understanding of the data. To reduce the impact of this limitation, in-country experts were consulted throughout the process to enhance comprehension of the data and its relevance to the local context.

Summary and conclusions

The process of selecting priority maternal and perinatal health recommendations and exploring barriers and facilitators to implementation of the four priority WHO guidelines yielded valuable information to inform implementation planning in Tanzania. The most salient points that emerged across the pre-workshop and workshop activities were as follows:

- Drug procurement, management, and distribution practices are not operating optimally. Key suggestions to improve drug ordering and monitoring across the country included accountability measures for timely request and reporting, and implementing cost-sharing programs. There is a need to ensure that oxytocin is stored at 2 – 8 degrees (Celsius) at all levels of handling to maintain the cold chain system. This may minimize “drug waste” (i.e., medications expiring or becoming unstable due to storage).
- Ensure access to equipment such as refrigerators for drug storage, and basic supplies such as gloves and blood pressure cuffs. Budgeting for essential equipment and supplies can be improved in a Comprehensive Council Health Plan to ensure adequate funding.
- Recruitment and retention strategies focusing on rural areas in particular should be strengthened to ensure adequate human resources for health. Cross-training existing staff in maternal and perinatal health so that they can be re-distributed within and across facilities as needed may reduce the burden on overworked teams in all areas of Tanzania.

- Increased opportunities for training are essential to improve the implementation of guideline recommendations, with more focus on pre-service training and a refresher course for in-service training. The training should be competency- based, and should include continuing medical education, supportive supervision and mentorship programs.
- A focus on interprofessional training and promotion of a collaborative health care team model was cited as a key area for improvement that could improve attitudes, buy-in, and provider confidence in implementing a guideline recommendation.
- There is a need to create more formal linkages between the various levels of health care facilities to better coordinate and standardize maternal health care for the community. Opportunities to form linkages through technology (e.g., telemedicine) are currently being piloted in Tanzania.
- There is a need for wider dissemination of guidelines beyond distributing them directly to users. This could be achieved through strategies such as mass media campaigns, educational materials and community champions.

The methods used to inform the implementation strategies discussed in this report and many of the barriers, facilitators, and resultant implementation strategies identified regarding the four WHO MNH guidelines are transferable to other priority areas and guidelines. Therefore, these findings can inform and be integrated into future barrier and facilitator assessments and guideline implementation planning initiatives in Tanzania.

To facilitate guideline implementation, as informed by the findings presented in this report, we recommend creating a working group for planning, championing, and evaluating guideline implementation activities in Tanzania. This working group would benefit from multidisciplinary participation and representation from multiple levels and geographic regions across the country, including (but not limited to): MoHSW Tanzania staff, representatives of professional associations and regulatory bodies, physicians, midwives, community health partners, and researchers. Implementation support in terms of training and coaching can be provided by the GREAT Network throughout the process to aid in-country stakeholders in achieving implementation goals.

References

1. McGlynn EA, *et al*.: **The quality of health care delivered to adults in the US**. *N Engl J Med* 2003, 348:2635-2645.

2. Davis D, Evans M, Jadad A, Perrier L, Rath D, Ryan D, Sibbald G, *et al*.: **The case for KT: shortening the journey from evidence to effect.** *BMJ* 2003, **327**(7405)**:**33-35.

3. Madon T, Hofman KJ, Kupfer L, Glass RI: **Public health. Implementation science.** *Science* 2007, **318**(5857)**:**1728-1729.

4. Shah BR, Mamdani M, Jaakkimainen L, Hux JE: **Risk modification for diabetic patients. Are other risk factors treated as diligently as glycemia?** Can J ClinPharmacol 2004; **11**(2):e239- e244.

5. Pimlott NJ, Hux JE, Wilson LM, Kahan M, Li C, Rosser WW: **Educating physicians to reduce benzodiazepine use by elderly patients: a randomized controlled trial**. CMAJ 2003; **168**(7):835-839.

6. Kennedy J, Quan H, Ghali WA, Feasby TE: **Variations in rates of appropriate and inappropriate carotid endarterectomy for stroke prevention in 4 Canadian provinces**. CMAJ 2004; **171**(5):455- 459.

7. Haines A, Kuruvilla S, Borchert M: **Bridging the implementation gap between knowledge and action for health**. Bull World Health Organ 2004, **82**(10):724- 731.

8. Ministry of Health and Social Welfare [United Republic of Tanzania] (2014). ***The National Road Map Strategic Plan to Accelerate Reduction of Maternal Newborn and Child Deaths in Tanzania 2008-2015. Sharpened One Plan April 2014****.* Dar Es Salaam.

9. World Health Organization: **WHO recommendations for the prevention and treatment of postpartum haemorrhage 2012**. http://www.who.int/reproductivehealth/publications/maternal_perinatal_health/9789241548502/en/ Accessed 12 Sep 2014.

10. World Health Organization: **WHO recommendations for the prevention and treatment of pre-eclampsia and eclampsia 2011**. http://www.who.int/reproductivehealth/publications/maternal_perinatal_health/9789241548335/en/ Accessed 12 Sep 2014.

11. World Health Organization: **WHO recommendations for induction of labour 2011**. http://www.who.int/reproductivehealth/publications/maternal_perinatal_health/9789241501156/en/ Accessed 12 Sep 2014.

12. World Health Organization: **WHO recommendations for augmentation of labour 2014**. http://www.who.int/reproductivehealth/publications/maternal_perinatal_health/augmentation-labour/en/ Accessed 12 Sep 2014.

13. **Tanzania Plan for Implementation of the Recommendations of the UN Commission on Life Saving Commodities on Women and Children**. August 2013.

14. Jones J, Hunter D: **Consensus methods for medical and health services research.** *BMJ* 1995, **311:**376-380.

15. Fitch K, *et al*.: *The RAND/UCLA appropriateness method user's manual*. RAND Corporation; 2001.

16. Braun V, Clarke V: **Using thematic analysis in psychology**. Qual Res in Psychol 2006; **3**(2): 77-101.

Appendix A: Pre-workshop survey

GREAT Project Assessment Survey - Tanzania

Introduction
Welcome to the GREAT Project (Guideline-driven, Research priorities, Evidence synthesis, Application of evidence, and Transfer of knowledge). The purpose of the project is to improve the quality of care for mothers and infants in Tanzania; to build capacity locally; and to develop a tailored strategy to implement the following four priority World Health Organization (WHO) guidelines on maternal and perinatal health:
Prevention and Treatment of Post-Partum Haemorrhage (2012);
http://www.who.int/reproductivehealth/publications/maternal_perinatal_health/9789241548502/en/
Prevention and Treatment of Pre-Eclampsia/Eclampsia (2011);
http://www.who.int/reproductivehealth/publications/maternal_perinatal_health/9789241548335/en/
Induction of Labour (2011);
http://www.who.int/reproductivehealth/publications/maternal_perinatal_health/9789241501156/en/
Augmentation of Labour (2014). http://apps.who.int/rhl/guidelines/augmentation-labour/en/

You are being invited to participate in a short survey to help the project team better understand the key priorities related to the identified WHO guidelines on maternal and perinatal health in the context of Tanzania.Participation in the survey will take approximately 15- 20 minutes of your time. Survey responses are anonymous and will be used to inform the proceedings of a two-day in-person workshop to be held in Dar es Salaam, Tanzania in November 2014.

By completing and submitting this survey, your consent to participate is implied.

If you have any questions about the survey, please contact one of the following individuals:
Dr. Mwifadhi Mrisho, Ifakara Health Institute, e:mail: mmrisho@ihi.or.tz
Dr. Godfrey Mbaruku, Ifakara Health Institute, e:mail: gmbaruku@ihi.or.tz
Thank you very much for your time and participation.

Section 1: Demographic Information

1. In which region/district/ward/village do you work? Please respond (if applicable) in the boxes provided below.

Region: ☐

District: ☐

Ward: ☐

Village: ☐

2. At what level of the health care system do you work? Please check all responses that apply.

National Hospital ☐
Consultant Hospital ☐
District Hospital ☐
Regional Referral Hospital ☐
Ministry of Health Headquarters ☐
Regional Health Team ☐
District Health Team ☐
Non-governmental Organization ☐

International Organization ☐
Professional Regulatory Body ☐
Professional Association ☐

3. What is your title/role description?

[ ]

4. How long have you been in this role?

- ○ Less than 1 year
- ○ 1-2 years
- ○ 3-5 years
- ○ 6-10 years
- ○ 11-20 years
- ○ More than 20 years

5. Please list up to five main tasks you carry out in your current job, in descending order, starting from the most common. Please respond in the box provided below.

1. [ ]
2. [ ]
3. [ ]
4. [ ]
5. [ ]

Section 2: Guideline Prioritization

In this section you are being asked to rate the priority of each of the four selected guidelines, relative to one another. Of the following four areas identified for the WHO maternal and perinatal guideline implementation, please rank in order of highest priority to lowest priority from your perspective. To make your selections, simply drag the topic area from the left side of the page and drop it in to the fields on the right side of the page, in order of priority (1= highest priority, 4= lowest priority).

	1 (highest priority)	2	3	4 (lowest priority)
Prevention and treatment of postpartum haemorrhage (PPH)	○	○	○	○
Pre-eclampsia / eclampsia	○	○	○	○
Induction of labour	○	○	○	○
Augmentation of labour	○	○	○	○

Section 3: Prioritization of Recommendations

In this section, you are being asked to select the priority areas from a list of recommendations for each of the four selected WHO guidelines on maternal and perinatal health: Prevention and Treatment of Postpartum Haemorrhage (2012) Prevention and Treatment of Pre-eclampsia and Eclampsia (2011) Induction of Labour (2011) Augmentation of Labour (2014) These guidelines will appear in a random order, not necessarily the order you prioritized them.

Prevention and Treatment of Postpartum Haemorrhage (PPH)

From the list of recommendations below, taken from the WHO guideline on the prevention and treatment of postpartum haemorrhage (PPH), please select the FIVE recommendations that you feel are/should be

priorities in Tanzania at this time. To make your selections, simply drag the recommendation from the left side of the page and drop it in to the fields on the right side of the page, in order of importance, where 1 = most important and 5 - least important. The strength and quality of the recommendation are provided in brackets (Strength, Quality).

	1 (most important)	2	3	4	5
The use of uterotonics for the prevention of PPH during the third stage of labour is recommended for all births (Strong, Moderate)	○	○	○	○	○
Oxytocin (10 IU, IV/IM) is the recommended uterotonic drug for the prevention of PPH (Strong, Moderate)	○	○	○	○	○
In settings where oxytocin is unavailable, the use of other injectable uterotonics (if appropriate ergometrine/ methylergometrine or the fixed drug combination of oxytocin and ergometrine) or oral misoprostol (600 µg) is recommended. (Strong, Moderate)	○	○	○	○	○
In settings where skilled birth attendants are not present and oxytocin is unavailable, the administration of misoprostol (600 µg PO) by community health care workers and lay health workers is recommended for the prevention of PPH.(Strong, Moderate)	○	○	○	○	○
In settings where skilled birth attendants are available, CCT is recommended for vaginal births if the care provider and the parturient woman regard a small reduction in blood loss and a small reduction in the duration of the third stage of labour as important (Weak, High)	○	○	○	○	○
Late cord clamping (performed after 1 to 3 minutes after birth) is recommended for all births while initiating simultaneous essential newborn care (Strong, Moderate)	○	○	○	○	○
Early cord clamping (	○	○	○	○	○
Sustained uterine massage is not recommended as an intervention to prevent PPH in women who have received prophylactic oxytocin. (Weak, Low)	○	○	○	○	○
Postpartum abdominal uterine tonus assessment for early identification of uterine atony is recommended for all women (Strong, Very low)	○	○	○	○	○
Oxytocin (IV or IM) is the recommended uterotonic drug for the prevention of PPH in caesarean section (Strong, Moderate)	○	○	○	○	○
Controlled cord traction is the recommended method for removal of the placenta in caesarean section (Strong, Moderate)	○	○	○	○	○
Intravenous oxytocin alone is the recommended uterotonic drug for the treatment of PPH (Strong, Moderate)	○	○	○	○	○
If intravenous oxytocin is unavailable, or if the bleeding does not respond to oxytocin, the use of intravenous ergometrine, oxytocin-ergometrine fixed dose, or a prostaglandin drug (including sublingual misoprostol, 800 µg) is recommended. (Strong, low)	○	○	○	○	○
The use of isotonic crystalloids is recommended in preference to the use of colloids for the initial intravenous fluid resuscitation of women with PPH (Strong, low)	○	○	○	○	○
Uterine massage is recommended for the treatment of PPH (Strong, very low)	○	○	○	○	○
If bleeding does not stop in spite of treatment using uterotonics and other available conservative interventions (e.g. uterine massage, balloon tamponade), the use of surgical interventions is recommended (Strong, very low)	○	○	○	○	○
The use of bimanual uterine compression is recommended as a temporizing measure until appropriate care is available for the treatment of PPH due to uterine atony after vaginal delivery. (Weak, very low)	○	○	○	○	○
The use of uterine packing is not recommended for the treatment of PPH due to uterine atony after vaginal birth (Weak, very low)	○	○	○	○	○

The use of ergometrine for the management of retained placenta is not recommended as this may cause tetanic uterine contractions which may delay the expulsion of the placenta. (Weak, very low)	○	○	○	○	○
A single dose of antibiotics (ampicillin or first-generation cephalosporin) is recommended if manual removal of the placenta is practised. (Weak, very low)	○	○	○	○	○
The use of formal protocols by health facilities for the prevention and treatment of PPH is recommended (Weak, moderate)	○	○	○	○	○
The use of formal protocols for referral of women to a higher level of care is recommended for health facilities (Weak, very low)	○	○	○	○	○
The use of simulations of PPH treatment is recommended for pre-service and in-service training programmes. (Weak, very low)	○	○	○	○	○
Monitoring the use of uterotonics after birth for the prevention of PPH is recommended as a process indicator for programmatic evaluation (Weak, very low)	○	○	○	○	○

Section 3: Prioritization of Recommendations

In this section, you are being asked to select the priority areas from a list of recommendations for each of the four selected WHO guidelines on maternal and perinatal health: Prevention and Treatment of Postpartum Haemorrhage (2012) Prevention and Treatment of Pre-eclampsia and Eclampsia (2011) Induction of Labour (2011) Augmentation of Labour (2014) These guidelines will appear in a random order, not necessarily the order you prioritized them.

Prevention and Treatment of Pre-Eclampsia and Eclampsia

From the list of recommendations below, taken from the WHO guideline on the prevention and treatment of pre-eclampsia and eclampsia, please select the FIVE recommendations that you feel are/should be priorities in Tanzania at this time. To make your selections, simply drag the recommendation from the left side of the page and drop it in to the fields on the right side of the page, in order of importance, where 1 = most important and 5 - least important. The strength and quality of the recommendation are provided in brackets (Strength, Quality).

	1 (most important)	2	3	4	5
In areas where dietary calcium intake is low, calcium supplementation during pregnancy (at doses of 1.5–2.0 g elemental calcium/day) is recommended for the prevention of pre-eclampsia in all women, but especially those at high risk of developing pre-eclampsia (Strong, moderate)	○	○	○	○	○
Low-dose acetylsalicylic acid (aspirin, 75 mg) is recommended for the prevention of pre-eclampsia in women at high risk of developing the condition. (Strong, moderate)	○	○	○	○	○
Low-dose acetylsalicylic acid (aspirin, 75 mg) for the prevention of pre-eclampsia and its related complications should be initiated before 20 weeks of pregnancy. (low, weak)	○	○	○	○	○
Women with severe hypertension during pregnancy should receive treatment with antihypertensive drugs. (Strong, very low)	○	○	○	○	○
Magnesium sulfate is recommended for the prevention of eclampsia in women with severe pre-eclampsia in preference to other anticonvulsants. (Strong, high)	○	○	○	○	○
Magnesium sulfate is recommended for the treatment of women with eclampsia in preference to other anticonvulsants. (Strong, moderate)	○	○	○	○	○
The full intravenous or intramuscular magnesium sulfate regimens are recommended for the prevention and treatment of eclampsia. ((Strong, moderate)	○	○	○	○	○

For settings where it is not possible to administer the full magnesium sulfate regimen, the use of magnesium sulfate loading dose followed by immediate transfer to a higher level health-care facility is recommended for women with severe pre-eclampsia and eclampsia. (Weak, very low)	○	○	○	○	○
Induction of labour is recommended for women with severe preeclampsia at a gestational age when the fetus is not viable or unlikely to achieve viability within one or two weeks. (Strong, very low)	○	○	○	○	○
In women with severe pre-eclampsia, a viable fetus and before 34 weeks of gestation, a policy of expectant management is recommended, provided that uncontrolled maternal hypertension, increasing maternal organ dysfunction or fetal distress are absent and can be monitored. (Weak, very low)	○	○	○	○	○
In women with severe pre-eclampsia, a viable fetus and between 34 and 36 (plus 6 days) weeks of gestation, a policy of expectant management may be recommended, provided that uncontrolled maternal hypertension, increasing maternal organ dysfunction or fetal distress are absent and can be monitored. (Weak, very low)	○	○	○	○	○
In women with severe pre-eclampsia at term, early delivery is recommended. (Strong, low)	○	○	○	○	○
In women with mild pre-eclampsia or mild gestational hypertension at term, induction of labour is recommended. (Weak, moderate)	○	○	○	○	○
In women treated with antihypertensive drugs antenatally, continued antihypertensive treatment postpartum is recommended. (Strong, very low)	○	○	○	○	○
Treatment with antihypertensive drugs is recommended for severe postpartum hypertension. (Strong, very low)	○	○	○	○	○
Advice to rest at home is not recommended as an intervention for the primary prevention of pre-eclampsia and hypertensive disorders of pregnancy in women considered to be at risk of developing those conditions. (Weak, low)	○	○	○	○	○
Strict bedrest is not recommended for improving pregnancy outcomes in women with hypertension (with or without proteinuria) in pregnancy. (Weak, low)	○	○	○	○	○
Restriction in dietary salt intake during pregnancy with the aim of preventing the development of pre-eclampsia and its complications is not recommended. (Weak, moderate)	○	○	○	○	○
Individual or combined vitamin C and vitamin E supplementation during pregnancy is not recommended to prevent the development of pre-eclampsia and its complications. (Strong, high)	○	○	○	○	○

Section 3: Prioritization of Recommendations

In this section, you are being asked to select the priority areas from a list of recommendations for each of the four selected WHO guidelines on maternal and perinatal health: Prevention and Treatment of Postpartum Haemorrhage (2012) Prevention and Treatment of Pre-eclampsia and Eclampsia (2011) Induction of Labour (2011) Augmentation of Labour (2014) These guidelines will appear in a random order, not necessarily the order you prioritized them.

Induction of Labour

From the list of recommendations below, taken from the WHO guideline for induction of labour, please select the <u>FIVE</u> recommendations that you feel are/should be priorities in Tanzania at this time. To make your selections, simply drag the recommendation from the left side of the page and drop it in to the fields on the right side of the page, in order of importance, where 1 = most important and 5 - least important. The strength and quality of the recommendation are provided in brackets (Strength, Quality).

	1 (most	2	3	4	5

	important)				
Induction of labour is recommended for women who are known with certainty to have reached 41 weeks (>40 weeks + 7 days) of gestation. (Weak, low)	○	○	○	○	○
If gestational diabetes is the only abnormality, induction of labour before 41 weeks of gestation is not recommended. (Weak, very low)	○	○	○	○	○
Induction of labour at term is not recommended for suspected fetal macrosomia. (Weak, low)	○	○	○	○	○
Induction of labour is recommended for women with prelabour rupture of membranes at term. (Strong, high)	○	○	○	○	○
If prostaglandins are not available, intravenous oxytocin alone should be used for induction of labour. Amniotomy alone is not recommended for induction of labour. (Weak, moderate)	○	○	○	○	○
Oral misoprostol (25 µg, 2-hourly) is recommended for induction of labour. (Strong, moderate)	○	○	○	○	○
Low-dose vaginal misoprostol (25 µg, 6-hourly) is recommended for induction of labour. (Strong, moderate)	○	○	○	○	○
Misoprostol is not recommended for induction of labour in women with previous caesarean section (Strong, low)	○	○	○	○	○
Low doses of vaginal prostaglandins are recommended for induction of labour. (Strong, moderate)	○	○	○	○	○
Balloon catheter is recommended for induction of labour (Strong, moderate)	○	○	○	○	○
The combination of balloon catheter plus oxytocin is recommended as an alternative method of induction of labour when prostaglandins (including misoprostol) are not available or are contraindicated. (Weak, low)	○	○	○	○	○
Sweeping membranes is recommended for reducing formal induction of labour. (Strong, moderate)	○	○	○	○	○
Outpatient induction of labour is not recommended for improving birth outcomes. (Low, weak)	○	○	○	○	○

Section 3: Prioritization of Recommendations

In this section, you are being asked to select the priority areas from a list of recommendations for each of the four selected WHO guidelines on maternal and perinatal health: Prevention and Treatment of Postpartum Haemorrhage (2012) Prevention and Treatment of Pre-eclampsia and Eclampsia (2011) Induction of Labour (2011) Augmentation of Labour (2014) These guidelines will appear in a random order, not necessarily the order you prioritized them.

Augmentation of Labour

From the list of recommendations below, taken from the WHO guideline for augmentation of labour, please select the FIVE recommendations that you feel are/should be priorities in Tanzania at this time. To make your selections, simply drag the recommendation from the left side of the page and drop it in to the fields on the right side of the page, in order of importance, where 1 = most important and 5 - least important. The strength and quality of the recommendation are provided in brackets (Strength, Quality).

	1 (most important)	2	3	4	5
Active phase partograph with a four-hour action line is recommended for monitoring the progress of labour. (Strong, very low)	○	○	○	○	○
Digital vaginal examination at intervals of four hours is recommended for routine assessment and identification of delay in active labour. (Weak, very low)	○	○	○	○	○
The use of early amniotomy with early oxytocin augmentation for prevention of delay in labour is not recommended. (Weak, very low)	○	○	○	○	○

The use of oxytocin for prevention of delay in labour in women receiving epidural analgesia is not recommended. (Weak, low)	○	○	○	○	○
The use of amniotomy alone for prevention of delay in labour is not recommended. (Weak, very low)	○	○	○	○	○
The use of antispasmodic agents for prevention of delay in labour is not recommended. (Weak, very low)	○	○	○	○	○
Pain relief for preventing delay and reducing the use of augmentation in labour is not recommended. (Weak, low)	○	○	○	○	○
The use of intravenous fluids with the aim of shortening the duration of labour is not recommended. (Strong, very low)	○	○	○	○	○
For women at low risk, oral fluid and food intake during labour is recommended. (Weak, very low)	○	○	○	○	○
Encouraging the adoption of mobility and upright position during labour in women at low risk is recommended. (Strong, very low)	○	○	○	○	○
Continuous companionship during labour is recommended for improving labour outcomes. (Strong, moderate)	○	○	○	○	○
The use of oxytocin alone for treatment of delay in labour is recommended. (Weak, very low)	○	○	○	○	○
Augmentation with intravenous oxytocin prior to confirmation of delay in labour is not recommended. (Weak, very low)	○	○	○	○	○
High starting and increment dosage regimen of oxytocin is not recommended for labour augmentation. (Weak, very low)	○	○	○	○	○
The use of oral misoprostol for labour augmentation is not recommended. (Strong, very low)	○	○	○	○	○
The use of amniotomy alone for treatment of delay in labour is not recommended (Weak, very low)	○	○	○	○	○
The use of amniotomy and oxytocin for treatment of confirmed delay in labour is recommended. (Weak, very low)	○	○	○	○	○

6. Are you aware of any other stakeholders in your community (for example members of the public, community leaders, health care managers, health care professionals, health care workers, policymakers, or key community members) who have knowledge of guideline implementation in the area of maternal and perinatal health and may be willing to participate in this survey?

Name 1:

Title/Role 1:

Email Address 1:

Mailing Address 1:

Name 2:

Title/Role 2:

Email Address 2:

Mailing Address 2:

7. Is there anything that you would like to add?

Thank you very much for participating in this survey.

Appendix B: Focus group discussion guides

**Focus Group Discussion Guide: Version DL, NL and IP FGD**	
**Question 1**	Keeping in mind the context of maternal and newborn health in Tanzania at this time, what three recommendations are the most important to implement for the:<br>- Induction of labour guideline?<br>- Augmentation of labour guideline?<br>- Prevention and treatment of PPH guideline?<br>- Prevention and treatment of preeclampsia and eclampsia guideline?
**Question 2**	Thinking about the top three recommendations for each guideline as a whole, what do you think are the potential barriers or challenges to implementing these guideline recommendations in Tanzania?
**Question 3**	Again, thinking about the top three recommendations for each guideline as a whole, what do you think are the potential facilitators that could aid in the implementation of these guidelines?
**Question 4**	Do you feel that there is sufficient readiness and buy-in in Tanzania to implement these guideline recommendations?
**Question 5**	Do you feel confident that these guideline recommendations can be adhered to? In other words, that the recommendations will be practiced and that practices will be sustained?
**Question 6**	How do you currently monitor/measure implementation efforts?
**Question 7**	Do you have any additional suggestions that could help with the implementation of the selected WHO guidelines?
**Question 8**	Before we wrap up today's discussion, is there anything else that anyone would like to add?

**Focus Group Discussion Guide: Version Clinician FGD**	
**Question 1**	Keeping in mind the context of maternal and newborn health in Tanzania at this time, what three recommendations are the most important to implement for the :<br>- Induction of labour guideline?<br>- Augmentation of labour guideline?<br>- Prevention and treatment of PPH guideline?<br>- Prevention and treatment of preeclampsia and eclampsia guideline?
**Question 2**	Which of these recommendations are within your scope of practice?<br>In other words, which guideline recommendations would you perform as part of your professional role if they were implemented?
**Question 3**	How confident do you feel in your knowledge to practice these recommendations? In your skills? In your ability?
**Question 4**	Thinking about the top three recommendations for each guideline as a whole, what do you think are the potential barriers or challenges to implementing these guideline recommendations in Tanzania?
**Question 5**	Again, thinking about the top three recommendations for each guideline as a whole, what do you think are the potential facilitators that could aid in the implementation of these guidelines?
**Question 6**	Do you feel confident that these guideline recommendations can be adhered to? In other words, that the recommendations will be practiced and that practices will be sustained?
**Question 7**	How do you currently monitor/measure maternal and newborn health practices?
**Question 8**	Do you have any additional suggestions that could help with the implementation of the selected WHO guidelines?<br>Is there anything else that anyone would like to add?

Appendix C: Pre-workshop survey findings for priority recommendations

Pre-workshop survey findings for priority recommendations for prevention and treatment of PPH guideline.

Prevention and treatment of PPH	Priority	n	N Total	Total Score
The use of uterotonics for the prevention of PPH during the third stage of labour is recommended for all births (Strong, Moderate)	1 (highest priority)	4	8	31
	2	1		
	3	2		
	4	0		
	5 (lowest priority)	1		
Oxytocin (10 IU, IV/IM) is the recommended uterotonic drug for the prevention of PPH (Strong, Moderate)	1 (highest priority)	3	7	31
	2	4		
	3	0		
	4	0		
	5 (lowest priority)	0		
In settings where oxytocin is unavailable, the use of other injectable uterotonics (if appropriate ergometrine/ methylergometrine or the fixed drug combination of oxytocin and ergometrine) or oral misoprostol (600 µg) is recommended. (Strong, Moderate)	1 (highest priority)	0	5	14
	2	1		
	3	3		
	4	0		
	5 (lowest priority)	1		
In settings where skilled birth attendants are available, CCT is recommended for vaginal births if the care provider and the parturient woman regard a small reduction in blood loss and a small reduction in the duration of the third stage of labour as important (Weak, High)	1 (highest priority)	0	4	11
	2	2		
	3	0		
	4	1		
	5 (lowest priority)	1		
In settings where skilled birth attendants are not present and oxytocin is unavailable, the administration of misoprostol (600 µg PO) by community health care workers and lay health workers is recommended for the prevention of PPH.(Strong, Moderate)	1 (highest priority)	0	5	9
	2	0		
	3	1		
	4	2		
	5 (lowest priority)	2		
Late cord clamping (performed after 1 to 3 minutes after birth) is recommended for all births while initiating simultaneous essential newborn care (Strong, Moderate)	1 (highest priority)	0	3	4
	2	0		
	3	0		
	4	1		
	5 (lowest priority)	2		

*Note: The number of respondents who identified a given recommendation within each of the five categories = n; the total number of respondents who selected the recommendation as one of their top five priorities = N Total; the calculated total score = Total Score. The total number of respondents (N Total) varies as it is dependent on the number of respondents who selected a given recommendation among their top five priorities.

Pre-workshop survey findings for priority recommendations for prevention and treatment of pre-eclampsia/eclampsia guideline.

Pre-eclampsia/eclampsia	Priority	n	N Total	Total Score
Magnesium sulfate is recommended for the treatment of women with eclampsia in preference to other anticonvulsants. (Strong, moderate)	1 (highest priority)	1	6	17
	2	2		
	3	0		
	4	1		
	5 (lowest priority)	2		
In areas where dietary calcium intake is low, calcium supplementation during pregnancy (at doses of 1.5–2.0 g elemental calcium/day) is recommended for the prevention of pre-eclampsia in all women, but especially those at high risk of developing pre-eclampsia (Strong, moderate)	1 (highest priority)	2	4	17
	2	1		
	3	1		
	4	0		
	5 (lowest priority)	0		
Magnesium sulfate is recommended for the prevention of eclampsia in women with severe pre-eclampsia in preference to other anticonvulsants. (Strong, high)	1 (highest priority)	2	4	15
	2	0		
	3	1		
	4	1		
	5 (lowest priority)	0		
Low-dose acetylsalicylic acid (aspirin, 75 mg) is recommended for the prevention of pre-eclampsia in women at high risk of developing the condition. (Strong, moderate)	1 (highest priority)	1	3	13
	2	2		
	3	0		
	4	0		
	5 (lowest priority)	0		
Women with severe hypertension during pregnancy should receive treatment with antihypertensive drugs. (Strong, very low)	1 (highest priority)	0	4	7
	2	0		
	3	1		
	4	1		
	5 (lowest priority)	2		

*Note: The number of respondents who identified a given recommendation within each of the five categories = n; the total number of respondents who selected the recommendation as one of their top five priorities = N Total; the calculated total score = Total Score. The total number of respondents (N Total) varies as it is dependent on the number of respondents who selected a given recommendation among their top five priorities.

Pre-workshop survey findings for priority recommendations for induction of labour guideline.

Induction of Labour	Priority	n	N Total	Total Score
Induction of labour is recommended for women with prelabour rupture of membranes at term. (Strong, high)	1 (highest priority)	2	5	21
	2	2		
	3	1		
	4	0		
	5 (lowest priority)	0		

If prostaglandins are not available, intravenous oxytocin alone should be used for induction of labour. Amniotomy alone is not recommended for induction of labour. (Weak, moderate)	1 (highest priority)	0	3	5
	2	0		
	3	1		
	4	0		
	5 (lowest priority)	2		
Low doses of vaginal prostaglandins are recommended for induction of labour. (Strong, moderate)	1 (highest priority)	0	3	7
	2	0		
	3	1		
	4	2		
	5 (lowest priority)	0		

*Note: The number of respondents who identified a given recommendation within each of the five categories = n; the total number of respondents who selected the recommendation as one of their top five priorities = N Total; the calculated total score = Total Score. The total number of respondents (N Total) varies as it is dependent on the number of respondents who selected a given recommendation among their top five priorities.

Pre-workshop survey findings for priority recommendations for augmentation of labour guideline.

Augmentation of Labour	Priority	n	N Total	Total Score
Active phase partograph with a four-hour action line is recommended for monitoring the progress of labour. (Strong, very low)	1 (highest priority)	6	8	38
	2	2		
	3	0		
	4	0		
	5 (lowest priority)	0		
Digital vaginal examination at intervals of four hours is recommended for routine assessment and identification of delay in active labour. (Weak, very low)	1 (highest priority)	1	6	23
	2	3		
	3	2		
	4	0		
	5 (lowest priority)	0		
Continuous companionship during labour is recommended for improving labour outcomes. (Strong, moderate)	1 (highest priority)	1	5	14
	2	1		
	3	0		
	4	2		
	5 (lowest priority)	1		
Encouraging the adoption of mobility and upright position during labour in women at low risk is recommended. (Strong, very low)	1 (highest priority)	0	3	7
	2	0		
	3	1		
	4	2		
	5 (lowest priority)	0		

*Note: The number of respondents who identified a given recommendation within each of the five categories = n; the total number of respondents who selected the recommendation as one of their top five priorities = N Total; the calculated total score = Total Score. The total number of respondents (N Total) varies as it is dependent on the number of respondents who selected a given recommendation among their top five priorities.

Appendix D: Median score and interquartile range (IQR) for feasibility rankings by guideline recommendation

	Recommendation	Score [median (IQR 25th ,75th)]
Prevention and treatment of PPH	Oxytocin (10 IU, IV/IM) is the recommended uterotonic drug for the prevention of PPH.	8 (8, 8)
	In settings where oxytocin is unavailable, the use of other injectable uterotonics (if appropriate ergometrine/methylergometrine or the fixed drug combination of oxytocin and ergometrine) or oral misoprostal (600 µg) is recommended.	9 (8, 9)
	The use of uterotonics for the prevention of PPH during the third stage of labour is recommended for all births.	9 (9, 9)
Prevention and Treatment of Pre-eclampsia / eclampsia	Magnesium sulfate is recommended for the prevention of eclampsia in women with severe pre-eclampsia in preference to other anticonvulsants	9 (9, 9)
	Magnesium sulfate is recommended for the treatment of women with eclampsia in preference to other anticonvulsants.	9 (8, 9)
	Women with severe hypertension during pregnancy should receive treatment with antihypertensive drugs.	7 (6, 8)
	For settings where it is not possible to administer the full magnesium sulfate regimen, the use of magnesium sulfate loading dose followed by immediate transfer to a higher level health care facility is recommended for women with severe pre-eclampsia and eclampsia.	8 (8, 9)
Induction of labour	Induction of labour is recommended for women who are known with certainty to have reached 41 weeks of gestation.	8 (7, 9)
	Induction of labour is not recommended in women with an uncomplicated pregnancy at gestational age less than 41 weeks.	8 (6, 9)
Augmentation of labour	Active phase partograph with a four-hour action line is recommended for monitoring the progress of labour.	9 (8, 9)
	Encouraging the adoption of mobility and upright position during labour in women at low risk is recommended.	8 (8, 9)
	Continuous companionship during labour is recommended for improving labour outcomes.	6 (4, 7)
