# Supplementary material for: Barriers, Facilitators and Priorities for Implementation of WHO Maternal and Perinatal Health Guidelines in Four Lower-Income Countries: A GREAT Network Research Activity
Source: PLoS One. 2016 Nov 2;11(11):e0160020. doi: 10.1371/journal.pone.0160020 (PMC5091885; doi:10.1371/journal.pone.0160020)
Supplement: S4 File — (PDF) [file pone.0160020.s004.pdf]

Understanding Barriers and Facilitators to Implementation of Maternal Health Guidelines in Ethiopia: A GREAT Network Research Activity

Final report on findings

Bishoftu, Ethiopia

4 and 5 May 2015

Prepared by:

*Caitlyn Timmings[1], Sobia Khan[1], Yolanda Scoleri[1], Dr. Lisa Puchalski Ritchie[1,3], Dr. Dina N. Khan[2], Dr. Joshua P. Vogel[2], Dr. Julia E. Moore[1], Shusmita Islam[1], Dr. Azmach Hadush[4], Dr. Luwam Teshome[5], Mr. Atkure Defar[6], Dr. Marta Minwyelet Terefe[4], Dr. A. Metin Gülmezoglu[2],*and *Dr.Sharon E. Straus[1,3]*

[1] Knowledge Translation Program, Li Ka Shing Knowledge Institute, St. Michael's Hospital, Canada

[2] UNDP/UNFPA/UNICEF/WHO/World Bank Special Programme of Research, Development and Research Training in Human Reproduction (HRP), Department of Reproductive Health and Research, World Health Organization, Geneva, Switzerland

[3] University of Toronto, Canada

[4] Federal Ministry of Health, Ethiopia

[5] World Health Organization Country Office, Ethiopia

[6] Ethiopian Public Health Institute

TABLE OF CONTENTS

ACKNOWLEDGEMENTS 2
CONTACT 2
ABBREVIATIONS 3
EXECUTIVE SUMMARY 4
BACKGROUND 7
Development of an International Partnership 7
Purpose of Report 8
METHODS 8
Participant Recruitment 8
Pre-Workshop Survey 8
In-Person Workshop 9
Focus Groups 9
Ranking Exercise 9
Small Group Discussions 9
Analysis 10
Triangulation of Methods 10
FINDINGS 11
Pre-workshop Survey 11
Section 1: Demographics 11
Section 2: Prioritizing Guidelines 12
Section 3: Prioritizing Recommendations within each Clinical Area 12
Focus Group Discussions 15
Health Care System Level 16
Health Care Provider Level 20
Pregnant Women and the Community Level 22
Ranking Exercise 23
Potential Implementation Strategies to Inform a Country-Specific Implementation Plan 24
LIMITATIONS 28
RECOMMENDATIONS AND CONCLUSION 28
REFERENCES 30
Appendix A: Pre-workshop survey 32
Appendix B: Focus group discussion guides 43
Appendix C: Pre-workshop survey findings on the selection and implementation of priority recommendations in the Prevention and Treatment of PPH guideline 47
Appendix D: Median score and interquartile range (IQR) for feasibility rankings for recommendations of the Prevention and Treatment of PPH guideline 49

ACKNOWLEDGEMENTS

We would like to formally thank the Federal Ministry of Health Ethiopia, Ethiopian Public Health Institute, World Health Organization (WHO) Country Office (Ethiopia), and PATH (Ethiopia) for graciously hosting us in Bishoftu, and wish to especially thank Dr. Azmach Hadush, Dr. Luwam Teshome, Mr. Atkure Defar, and Dr. Marta Minwyelet Terefe for their expertise and guidance throughout the process. We would also like to thank Dr.Terefe Gelibo for his support in transcription and translation of qualitative data. We would like to acknowledge WHO, PATH, and the United Nations Commission on Life Saving Commodities for Women and Children for funding the project activities. We also wish to acknowledge the GREAT Network for their strategic guidance in designing this activity. This activity is a part of a series of projects that the GREAT Network is involved in partnership with low and middle income countries (website: http://greatnetworkglobal.org/).

CONTACT

For questions about this report, please contact:

Caitlyn Timmings, MPH
Research Coordinator
Knowledge Translation Program
Li Ka Shing Knowledge Institute
St. Michael's Hospital
Toronto, Canada
Email: timmingsc@smh.ca
Phone: 416-864-6060 ext. 77566

ABBREVIATIONS

**CO**	Country Office
**EPHI**	Ethiopian Public Health Institute
**FG**	Focus group
**FMoH**	Federal Ministry of Health (Ethiopia)
**GREAT**	Guideline-driven, Research priorities, Evidence synthesis, Application of evidence, and Transfer of knowledge
**HCWs**	Health care workers
**IV**	Intravenous
**KT**	Knowledge translation
**LMIC**	Low and middle income country
**MDG**	Millennium Development Goal
**MWs**	Midwives
**PPH**	Postpartum haemorrhage
**SMH**	St. Michael’s Hospital
**UN**	United Nations
**WHO**	World Health Organization

EXECUTIVE SUMMARY

Background

An international collaboration was formed between the World Health Organization (WHO, Switzerland), St. Michael's Hospital (SMH, Canada), Federal Ministry of Health (FMoH) Ethiopia, Ethiopian Public Health Institute (EPHI), WHO Country Office (CO) Ethiopia, and PATH (Ethiopia) to strategize the implementation of the WHO guideline on the Prevention and Treatment of Postpartum Haemorrhage (PPH) (2012) (http://apps.who.int/iris/bitstream/10665/75411/1/9789241548502_eng.pdf) in the Ethiopian context.

An in-country workshop was held in May 2015 in Bishoftu, Ethiopia to determine key recommendations that will inform the development of a multi-level implementation strategy for improving use of the WHO guidelines nationally. Recommendations resulted from:

1) Identifying barriers and facilitators to the implementation of the guideline in Ethiopia;
2) Identifying the most important and feasible recommendations for implementation; and
3) Providing suggestions for potential implementation strategies based on the barriers and facilitators identified, and the perceived feasibility of implementation.

The purpose of this report is to provide health care system stakeholders in Ethiopia with key findings from pre-workshop and workshop activities and to inform future implementation activities to optimize application of these guidelines.

Methods

A mixed methods approach was used to collect data on priorities, barriers, facilitators, and potential implementation strategies for the WHO recommendations on PPH in Ethiopia. Prior to the workshop, a survey was administered to inform workshop proceedings.

Primary data collection occurred during the in-country two-day workshop; this involved focus group discussions, an individual ranking exercise, and small and large group discussions. The workshop aimed to explore barriers and facilitators to guideline implementation; identify guideline priority areas; and develop potential implementation strategies to fit the local context.

Findings

Fifty three stakeholders participated in the pre-workshop survey and nineteen stakeholders participated in the in-person workshop. Stakeholders represented multiple disciplines from diverse geographic regions and levels of the health care system including: health care administrators, policymakers, non-governmental organization staff, representatives from professional associations, frontline health care providers (e.g., physicians and midwives), and health system researchers/academics.

Findings from the pre-workshop survey identified 'use of uterotonics' as the highest priority (66% of respondents) of the four clinical areas in which the recommendations of the Prevention and Treatment of PPH guideline were grouped. A total of five recommendations received a median score of 5 (5= extremely well implemented), when respondents were asked how well the guideline recommendations were currently being implemented in their individual settings from their experience.

Findings from the focus group discussions described issues at the level of the health care system, which included factors related to policies and wider systemic conditions in Ethiopia that can affect

implementation of the WHO guideline recommendations. These factors included: access to resources (e.g., drugs, supplies, personnel, facilities); drug procurement, distribution, management; data collection and monitoring; policies and incentives; readiness for change; and guidelines and protocols. Issues at the level of the health care provider that may affect guideline implementation were prevalent, and included: beliefs, attitudes, and buy-in about the use of guideline recommendations; knowledge and skills needed to implement the guidelines; training and supportive supervision around guideline implementation; and role definition. Finally, issues at the level of the patient/community that may affect guideline implementation included: traditional beliefs; knowledge and awareness; and access to health care services.

The ranking exercise resulted in an assessment of the feasibility of 11 guideline recommendations that were deemed to be priorities in Ethiopia by participants. Within subsequent small group discussions, multiple implementation strategies were suggested to overcome barriers.

Recommendations and conclusion

Informed by the findings of the pre-workshop and workshop activities, the following ten recommendations have been developed to guide next steps of guideline implementation in Ethiopia:

- **Recommendation #1**: Create a guideline implementation working group (WG) as a sub-group of the Federal Ministry of Health's maternal health case team. This guideline implementation WG should be multi-disciplinary and include representation from multiple levels.

The following six recommendations are intended for operationalization by the guideline implementation WG:

- **Recommendation #2**: Adapt the WHO maternal health guideline on Prevention and Treatment of PPH for the Ethiopian context using the ADAPTE process.
- **Recommendation #3**: Create standard protocols on how to implement the guideline recommendations and distribute to facilities for onsite guidance. Protocols should be user-friendly, ready-to-use, and visible (e.g., posted on wards) to act as reminders for HCWs.
- **Recommendation #4**: Select and implement priority clinical indicators as part of a monitoring and evaluation strategy on PPH prevention and management to enable systematic and standardized assessment of guideline implementation.
- **Recommendation #5**: Establish a mentorship program at the facility level between junior and senior HCWs to provide technical support and supportive supervision on implementation of the guideline recommendations protocols.
- **Recommendation #6**: Establish an interdisciplinary quality improvement team (e.g., including physicians, midwives, administrators) at each health care facility to identify priority areas for practice improvement based on the clinical indicators identified in recommendation #4. Quality improvement teams should develop and monitor quality improvement strategies for the priority areas at the facility level.
- **Recommendation #7**: Design and conduct a process and outcome evaluation of the guideline implementation approach.

The following three recommendations are intended for the guideline implementation WG in partnership with other key stakeholders (e.g., professional associations, other ministries, independent evaluators, etc.):

- **Recommendation #8**: Identify strategies to improve and standardize the benefits package offered to HCWs across all regions so that HCWs in rural regions receive the same compensation package as HCWs in urban regions.
- **Recommendation #9**: Conduct a process evaluation of the Health Extension Worker Program to improve functioning of the program in regions where it is not optimally working and share lessons learned from those regions where the program is working.
- **Recommendation #10**: Evaluate the Maternity Waiting Home initiative, which is currently being used in some remote areas to mitigate barriers experienced with the transportation to health facilities for deliveries during the rainy season. Key evaluation outcomes to consider could include: increased number of women giving birth at health care facilities, decreased incidence of PPH cases, decreased incidence of maternal deaths from PPH cases). If successful outcomes are demonstrated, consider scale up of this program.

Many of the barriers, facilitators, and resultant implementation strategies identified regarding the prioritized recommendations are applicable to other priority areas in health care; therefore, findings from this report can inform and be integrated into future barrier and facilitator assessments and guideline implementation planning initiatives in Ethiopia and in similar LMICs.

BACKGROUND

Despite a growing body of knowledge to support the use of evidence-based guidelines in clinical practice, health care systems worldwide are failing to use research evidence optimally to improve the quality of health care delivery[1]. Inadequate use of evidence in practice often results in inefficiencies, and reduced quantity and quality of life[1-6].

One of the health-related Millennium Development Goals (MDG) is to reduce maternal mortality by 75% between 1990 and 2015. The maternal mortality ratio for Ethiopia in 2013 was 420 maternal deaths per 100,000 live births[7]. A key barrier to reducing maternal mortality is lack of access to quality care during and after childbirth[7]. Furthermore, postpartum haemorrhage (PPH) has been identified as one of the main causes of maternal mortality in Ethiopia[8]. PPH-related maternal mortality has also been linked to mothers giving birth at home, without a skilled birth attendant present[9]. The current skilled birth attendant rate in Ethiopia is only 15%[10]. Evidence-based guidelines, which outline best clinical practice in maternal and perinatal health care, exist to address these gaps; however, low and middle-income countries (LMICs), including Ethiopia, often face numerous challenges in applying research evidence[11]. Recognizing these challenges faced by LMICs, there is a growing interest in how knowledge translation (KT) approaches can be tailored and applied to the area of maternal and perinatal health, and PPH specifically, to improve implementation of evidence-based clinical practices.

The World Health Organization (WHO) has partnered with the KT Program based at St. Michael's Hospital (SMH) in Toronto, Canada to establish an international partnership called the GREAT (Guideline-driven, Research priorities, Evidence synthesis, Application of evidence, and Transfer of knowledge) Network, funded by the Canadian Institutes of Health Research (www.greatnetworkglobal.org). The GREAT Network uses a unique evidence-based KT approach to support LMICs in implementing evidence-based clinical guidelines that can reduce maternal morbidity and mortality. The GREAT Network brings together relevant health care system stakeholders in LMICs to identify and assess the priorities, barriers, and facilitators related to guideline implementation in the area of maternal health. Furthermore, the GREAT Network supports the efforts of these key stakeholders to develop, operationalize, evaluate, and ultimately, sustain a guideline implementation strategy tailored to identified barriers, facilitators and priorities.

Development of an International Partnership

A partnership was established between the KT Program at SMH, WHO (Department of Reproductive Health and Research, Switzerland), PATH, and health care system stakeholders of Ethiopia including FMoH, Ethiopian Public Health Institute [EPHI], and WHO Ethiopia Country Office [CO], to provide technical support to increase the uptake of evidence-based maternal health guidelines in Ethiopia.

The objectives of this partnership include:

1. Providing key recommendations to inform the development of a multi-level implementation strategy for improving use of guidelines nationally;
2. Supporting local stakeholders in the development and delivery of the implementation strategy; and
3. Supporting local stakeholders in the development of a monitoring and evaluation plan to assess impact of guideline implementation and scale up and sustainability efforts.

The initial activity conducted as part of this partnership was a pre-workshop survey, followed by an in-country workshop, funded by WHO, PATH, and the United Nations (UN) Commission on Life-Saving Commodities (UNCoLSC) for Women and Children. Informed by joint in-country consultations with FMoH,

EPHI, PATH, and WHO CO Ethiopia, the WHO guideline on Prevention and Treatment of PPH (2012)[12] was selected as a key priority for the in-country workshop and related implementation activities.

Purpose of Report

This report provides key findings from the in-country workshop held in May 2015 and a pre-workshop survey. The aim of the in-country research activities was to meet the first objective of the international partnership: to provide key recommendations to inform the development of a multi-level implementation strategy for improving use of the selected WHO guideline in Ethiopia.

METHODS

A mixed methods approach was utilized which is outlined briefly below and included the following data collection activities:

1) a pre-workshop survey;
2) in-workshop focus groups and small group discussions; and
3) an in-workshop ranking exercise.

Participant Recruitment

Participants were identified in consultation with FMoH, EPHI, PATH, and the WHO Ethiopia CO. To ensure representation from across the health care system, individuals with roles as health care administrators, policymakers, non-governmental organization (NGO) staff, representatives from professional associations (e.g., Ethiopian Midwives' Association, Ethiopian Society of Obstetricians and Gynecologists), frontline health care providers (e.g., physicians, midwives, nurses), and health system researchers/academics were identified. Individuals representing different levels of the health care system were also identified to ensure representation from different types of health facilities, including district, regional, and referral hospitals. Geography was a key consideration in participant selection to ensure representation of stakeholders from both rural and urban centers across the country.

Pre-Workshop Survey

The pre-workshop survey was designed to inform workshop proceedings and provide a preliminary understanding of key priorities related to the WHO guideline on the Prevention and Treatment of PPH in the Ethiopian context. Surveys [**Appendix A**] were administered during April 2015 to relevant stakeholders of the health care system using both an electronic and paper-based version. A total of 82 stakeholders were invited to participate in the survey. Forty stakeholders received an email inviting them to participate in the electronic survey along with a link to the web-based survey platform. Forty-two stakeholders received the paper-based survey by post along with an invitation letter to participate in the survey. Stakeholders who received the survey by post were those who resided in remote geographic locations and/or who did not have internet access readily available. Consent was implied by completion of the survey. The survey was divided into 3 sections:

Section 1: Demographic information

Participants were asked a series of questions on their role/profession, number of years in their current role, geographic location).

Section 2: Prioritization of clinical areas in the guideline

In this section, participants were asked to prioritize guideline recommendations for the Prevention and Treatment of PPH based on the following four clinical areas: 1) use of uterotonics; 2) cord clamping; 3) uterine massage; and 4) protocols/training using a scale from 1 to 4, where 1 = first priority and 4 = fourth priority.

Section 3: Selection and implementation of recommendations within each clinical area in the guideline

Participants were asked to select recommendations within each of the four clinical areas that they felt were priorities in Ethiopia at this time. They were then asked to rate, from their perspective/experience, the level of implementation of each selected recommendation in their current work setting using a scale of 1 to 5, where 1 = not at all implemented and 5 = extremely well implemented.

In-Person Workshop

A sample of survey respondents and additional participants who represented the stakeholder groups of interest (described above) were invited to participate in a two-day in-person workshop held in Bishoftu, Ethiopia. At the workshop, stakeholders participated in focus group (FG) discussions on Day One, and in a ranking exercise and small group discussion on Day Two.

Focus Groups

Participants were divided into two FGs (approximately six to 13 participants per group). FGs were organized according to role and/or level of the health care system:

1) Midwives (MWs) group; and
2) Mixed group (including physicians, researchers, health care administrators, FMoH officials, NGO representatives, and professional association representatives).

FG sessions lasted approximately 90 minutes and were conducted in Amharic (MWs group) and English (Mixed group) using a semi-structured discussion guide [**Appendix B**]. The FGs centered on identifying priority recommendations from the Prevention and Treatment of PPH guideline based on perceived importance. Barriers and facilitators to implementing these recommendations in the Ethiopian context were also identified.

Ranking Exercise

A shortlist of recommendations was generated based on selections made in the Day One FGs and following deliberations among facilitators and local experts. On Day Two, workshop facilitators engaged participants in a modified Delphi[13] process to rate the feasibility of implementing each of the identified guideline recommendations. Consistent with the RAND Appropriateness Method[14], participants individually ranked each recommendation, using a 9-point Likert scale (where 1= extremely not feasible and 9= extremely feasible). When responses were highly disparate, large group discussion took place and responses were re-ranked with the aim of reaching a higher level of agreement.

Small Group Discussions

Following the ranking exercise, small group breakout discussions were conducted by facilitators using the same two groupings as used in FGs (i.e., MWs and mixed group). Participants were guided in an exercise to map implementation barriers to the priority recommendations, followed by an exercise to identify context appropriate implementation strategies that could address identified barriers.

Analysis

For the pre-workshop survey, descriptive statistics were used to analyze categorical and ordinal data. For Section 2 (prioritization of clinical areas in the guideline), weighted mean was calculated for each clinical area, whereby data were re-coded so that the highest ranking received the highest score (e.g., 1st ranked priority was assigned a score of 4). For Section 3 (selection and implementation of recommendations within each clinical area in the guideline), count was used to depict the number of respondents who identified the corresponding recommendation as a top priority. Respondent ratings of how well the recommendations that they identified as top priorities were currently being implemented in Ethiopia were analyzed using descriptive statistics [median, inter-quartile range (IQR) including the score for the 25th and 75th percentile].

FG sessions and small group discussions were digitally recorded and detailed notes were taken to supplement recordings. Data from the mixed group discussion were transcribed while those from the MWs group discussion were simultaneously transcribed and translated from Amharic to English. After familiarization of the data from the recordings and notes, data were qualitatively analyzed by an expert analyst at SMH using a thematic analysis approach[15]. Themes were developed in consultation with meeting facilitators to discuss interpretations of the data for a shared understanding of key findings.

Results from the individual ranking exercise were analyzed using descriptive statistics [median, interquartile range (IQR) including the score for the 25th and 75th percentile] of participant assigned feasibility ratings for each of the identified recommendations. Small group discussions were analyzed using the same methods as described for the FG sessions above.

Triangulation of Methods

Using the technique of integration, data collected across all methodologies were considered in detail to draw meaningful and pertinent recommendations that are feasible to implement and relevant for the Ethiopian context.

FINDINGS

Pre-Workshop Survey

Survey findings are presented below according to responses related to: (1) priority clinical areas of the WHO recommendations for the Prevention and Treatment of PPH; (2) priority recommendations within each clinical area; and (3) how well these recommendations are currently being implemented in Ethiopia. Demographic information of the survey respondents is also provided.

Section 1: Demographics

Fifty-three stakeholders participated in the pre-workshop survey (response rate = 65%). A description of the respondents is provided in **Table 1.** Survey respondents represented five different regions across Ethiopia. Addis Ababa was the most highly represented region (62.3%). Survey respondents also varied in the level of the health care system in which they were situated. Of note, 24.5% of respondents identified working in health center settings (i.e., at the primary level of care). Respondents reported various years in their current role, with 3-5 years and 1-2 years as the most common categories (37.7% and 26.4%, respectively).

Table 1. Demographic information of pre-workshop survey respondents

**Region (categories are not mutually exclusive)**	n (n=53)	%
Addis Ababa	33	62.3
Gambela	9	17.0
Somali	8	15.1
Southern Nations, Nationalities, and Peoples' Region	2	3.8
Amhara	1	1.9
**Level of the health care system (categories are not mutually exclusive)**	n (n=53)	%
Health Center	13	24.5
Ministry of Health	8	15.1
General Hospital	8	15.1
International Organization	7	13.2
District Hospital	4	7.5
Regional Referral Hospital	4	7.5
Specialized Hospital	3	5.7
Non-governmental Organization	3	5.7
Other (e.g., researchers/academics)	3	5.7
Regional Health Bureau	2	3.8
District Health Office	1	1.9
Non-Governmental organization supported clinic	1	1.9
Professional Association	1	1.9
**Number of Years in Current Role**	n (n=53)	%
3-5 years	20	37.7
1-2 years	14	26.4
Less than 1 year	6	11.3
6-10 years	6	11.3
11-20 years	5	9.4
More than 20 years	2	3.8

Section 2: Prioritizing Guidelines

Table 2 provides the ranking results and weighted mean for each of the four clinical areas of the guideline of interest. The clinical area of 'use of uterotonics' was ranked as the highest priority by 66% of respondents (weighted mean=3.64). The areas of 'protocols/training' and 'cord clamping' emerged as equal priority areas from participant ranking data (weighted mean=2.25 for both areas). The clinical area of 'uterine massage' was ranked as the lowest priority (weighted mean=1.87).

Table 2. Clinical areas of the WHO Prevention and Treatment of PPH guideline ranked in order of importance by pre-workshop survey respondents

<table>
<tr><th>Prioritization of Clinical Areas in the Prevention and Treatment of PPH Guideline</th><th>Priority</th><th>n
(N=53)</th><th>Weighted mean*</th></tr>
<tr><td rowspan="4">Recommendations related to the clinical area of Use of Uterotonics</td><td>1 (highest priority)</td><td>35</td><td rowspan="4">3.64</td></tr>
<tr><td>2</td><td>17</td></tr>
<tr><td>3</td><td>1</td></tr>
<tr><td>4 (lowest priority)</td><td>0</td></tr>
<tr><td rowspan="4">Recommendations related to the area of Protocols/Training</td><td>1 (highest priority)</td><td>15</td><td rowspan="4">2.25</td></tr>
<tr><td>2</td><td>4</td></tr>
<tr><td>3</td><td>13</td></tr>
<tr><td>4 (lowest priority)</td><td>21</td></tr>
<tr><td rowspan="4">Recommendations related to the clinical area of Cord Clamping</td><td>1 (highest priority)</td><td>2</td><td rowspan="4">2.25</td></tr>
<tr><td>2</td><td>20</td></tr>
<tr><td>3</td><td>20</td></tr>
<tr><td>4 (lowest priority)</td><td>11</td></tr>
<tr><td rowspan="4">Recommendations related to the clinical area of Uterine Massage</td><td>1 (highest priority)</td><td>1</td><td rowspan="4">1.87</td></tr>
<tr><td>2</td><td>12</td></tr>
<tr><td>3</td><td>19</td></tr>
<tr><td>4 (lowest priority)</td><td>21</td></tr>
</table>

*****Note: For the weighted mean calculation, each individual's response was given equal weight/value. Higher ranked priority items were re-coded and assigned a higher weight so that the highest ranking received the highest score (e.g., 1st ranked priority was assigned a score of 4).

Section 3: Prioritizing Recommendations within each Clinical Area

Recommendations identified by survey respondents as current priorities in Ethiopia for each of the four clinical areas are presented below with the strength of recommendation and quality of evidence indicated in parentheses, that is (strength of recommendation, quality of evidence). The median rank score is related to how well each priority recommendation is currently being implemented in the respondents current setting (i.e., is not reflective of implementation level country-wide). Detailed results are presented in **Appendix C**.

Use of Uteronics

Appendix C Table A provides the ranking results for the priority recommendations identified in the clinical area of 'use of uterotonics'. The following five recommendations were deemed to be the highest priorities, using a scale of 1 to 5, where 1 = not at all implemented and 5 = extremely well implemented, based on total count of responses received (n):

- The use of uterotonics for the prevention of PPH during the third stage of labour is recommended for all births (Strong, Moderate) (n=43 respondents selected as priority).
    - This recommendation was overall considered to be implemented extremely well by respondents (median score = 5).
- Oxytocin (10 IU, IV/IM) is the recommended uterotonic drug for the prevention of PPH (Strong, Moderate) (n=35 respondents selected as priority).
    - This recommendation was overall considered to be extremely well implemented by respondents (median score = 5).
- In settings where oxytocin is unavailable, the use of other injectable uterotonics (if appropriate ergometrine/ methylergometrine or the fixed drug combination of oxytocin and ergometrine) or oral misoprostol (600 µg) is recommended (Strong, Moderate).
    - This recommendation was overall considered to be well-implemented by respondents (median score = 4) (n=31 respondents selected as priority).
- In settings where skilled birth attendants are not present and oxytocin is unavailable, the administration of misoprostol (600 µg PO) by community health care workers and lay health workers is recommended for the prevention of PPH (Strong, Moderate).
    - This recommendation was overall considered to be well-implemented by respondents (median score = 4) (n=27 respondents selected as priority).
- If bleeding does not stop in spite of treatment using uterotonics and other available conservative interventions (e.g. uterine massage, balloon tamponade), the use of surgical interventions is recommended (Strong, very low).
    - This recommendation was overall considered to be well-implemented by respondents (median score = 4) (n=24 respondents selected as priority).

<u>Cord Clamping</u>
Appendix C Table B provides the ranking results for the priority recommendations identified in the clinical area of ‘cord clamping’. The following two recommendations were deemed to be the highest priorities, using a scale of 1 to 5, where 1 = not at all implemented and 5 = extremely well implemented, based on total count of responses received (n):

- In settings where skilled birth attendants are available, controlled cord traction (CCT) is recommended for vaginal births if the care provider and the parturient woman regard a small reduction in blood loss and a small reduction in the duration of the third stage of labour as important (Weak, High). This recommendation was considered to be implemented extremely well by respondents (median score = 5) (n=36 respondents selected as priority); and
- Early cord clamping (<1 minute after birth) is not recommended unless the neonate is asphyxiated and needs to be moved immediately for resuscitation (Strong, Moderate). This recommendation was considered to be implemented extremely well by respondents (median score = 5) (n=28 respondents selected as priority).

Uterine Massage

Appendix C Table C provides the ranking results for the priority recommendations identified in the clinical area of 'uterine massage'. The following two recommendations were deemed to be the highest priorities, using a scale of 1 to 5, where 1 = not at all implemented and 5 = extremely well implemented, based on total count of responses received (n):

- Postpartum abdominal uterine tonus assessment for early identification of uterine atony is recommended for all women (Strong, very low). This recommendation was considered to be implemented well by respondents (median score = 4) (n=43 respondents selected as priority); and
- Uterine massage is recommended for the treatment of PPH (Strong, very low). This recommendation was considered to be implemented extremely well by respondents (median score = 5) (n=28 respondents selected as priority).

Use of Protocols/Training

Appendix C Table D provides the ranking results for the priority recommendations identified in the clinical area of 'protocols/training'. The following two recommendations were deemed to be the highest priorities, using a scale of 1 to 5, where 1 = not at all implemented and 5 = extremely well implemented, based on total count of responses received (n):

- The use of formal protocols by health facilities for the prevention and treatment of PPH is recommended (Weak, moderate). This recommendation was considered to be implemented well by respondents (median score = 4) (n=48 respondents selected as priority); and
- The use of simulations of PPH treatment is recommended for pre-service and in-service training programmes (Weak, very low). This recommendation was considered to be implemented poorly by respondents (median score = 3) (n=28 respondents selected as priority).

Focus Group Discussions

Key discussion points have been synthesized across FGs and are organized into the following categories to reflect factors that influence implementation of priority guideline recommendations and operate at the level of: (1) the health care system; (2) the provider; and (3) pregnant women and the community. Major themes and sub-themes were identified at each level (Figure 1).

Figure 1. Overview of FG discussion findings on barriers and facilitators to guideline implementation

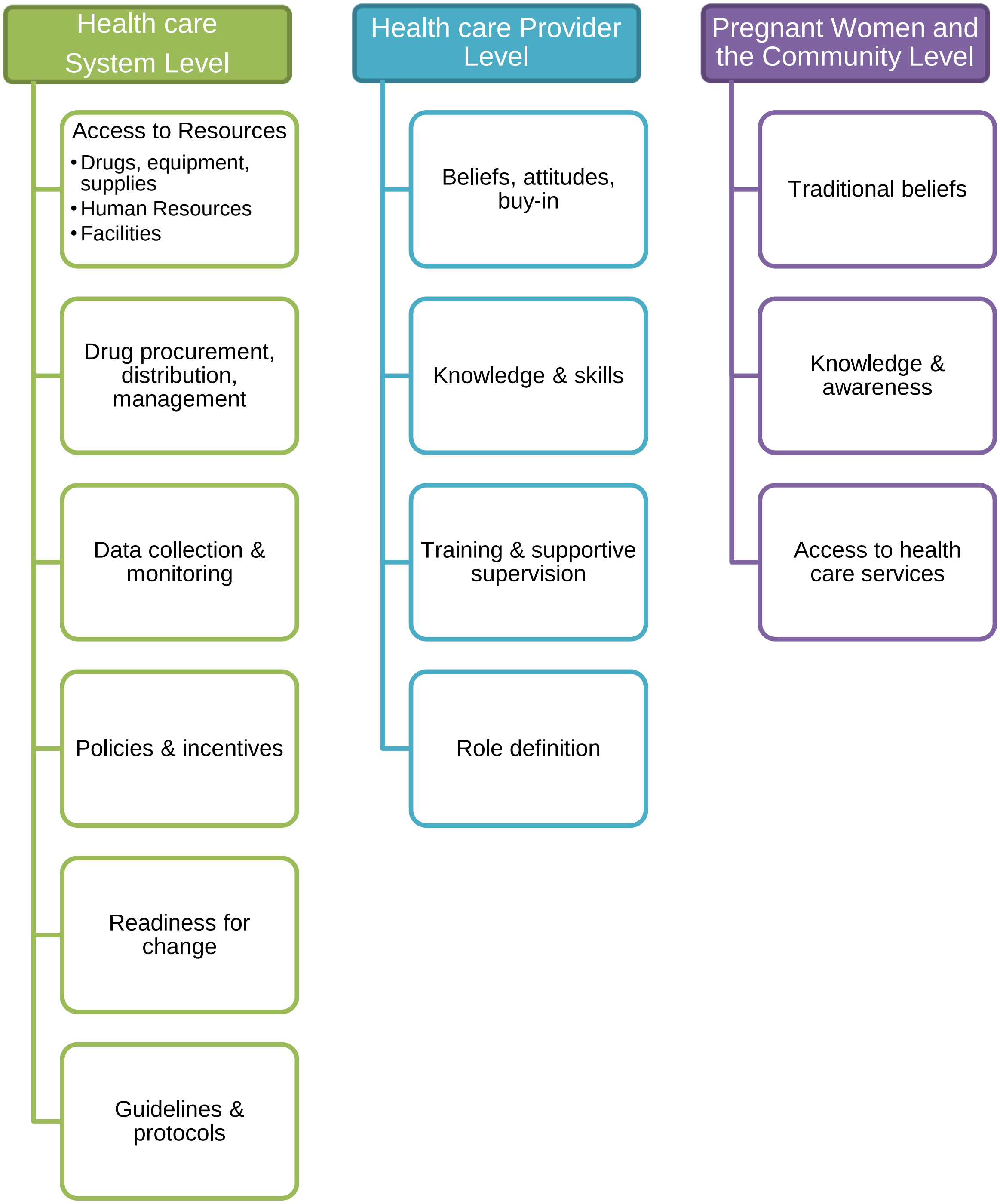

Health Care System Level

Factors were identified at the health care system level that can influence implementation of the WHO guideline recommendations. Factors in the following categories were described by participants: access to resources; drug procurement, distribution, management; data collection and monitoring; policies and incentives; readiness for change; and guidelines and protocols.

<u>Access to Resources</u>

Drugs, equipment, supplies

Participants considered the availability of drugs, equipment, and supplies as an important factor in determining the implementability of guideline recommendations in the Ethiopian context. Discussion occurred in both FGs around the appropriate selection of uterotonic agents, specifically misoprostol and oxytocin. Adequate refrigeration equipment and electricity governs whether misoprostol or oxytocin is used as the first line uterotonic agent. In rural health centers, where these commodities are scarce, misoprostol is more commonly used. In higher level, typically urban facilities, oxytocin is preferred. Both uterotonic agents were perceived by participants to be cheap, effective and safe. Participants also discussed the importance of bimanual compression, a simple intervention available without any additional cost. The challenges associated with PPH management related to the availability of additional supplies including intravenous (IV) uterotonic drugs, antibiotics, and catheters.

Resources are not equally available across health centers, and accessibility varies with the type of resource. For example, participants expressed a common belief that IV antibiotics and IV fluids are generally readily available for the prevention/ management of PPH in both rural and urban centers. This was perceived to be a facilitator for implementation of the guideline recommendations. Conversely, the shortage of blood at rural health facilities was perceived to be a prominent barrier to the management of PPH. Participants further noted that due to this shortage, IV fluids are used for emergency resuscitation and thus the use of IV fluids is also a priority. The general lack of supplies was noted as a barrier because even with the proper training, if the materials/supplies are not available, the guideline recommendations cannot be implemented.

The need for environmental restructuring due to limitations of health facilities was also discussed. Participants cited lack of access to blood transfusion facilities and intensive care units as barriers to guideline implementation. They also reported that blood services may be far from patients and blood boxes may not be available at primary hospitals and health centers. Participants from both FGs raised the issue of resources required for simulation training including a lack of simulation equipment (e.g., simulators) and physical space for simulators in health centers. Participants also cited transportation and ambulance services as a barrier to guideline implementation.

Human Resources
The availability of human resources was identified as an important factor in the implementation of the PPH guideline in the Ethiopian context. Issues regarding both recruitment and distribution/redistribution of health care workers (HCWs) were discussed by participants. Participants felt that there are an insufficient number of skilled HCWs/attendants nationally. Furthermore, the distribution of HCWs between urban and rural centers remains an issue; the current FMoH policy of MW distribution is a minimum of two per health center, but participants considered this to be an insufficient national standard. The disparities in the presence of skilled HCWs in urban versus rural health facilities, especially with regards to the number of MWs available per shift, was indicated as an important gap. For example, a participant shared that there might be up to eight MWs assigned per shift at health facilities in Addis Ababa versus zero to two midwives assigned to work the whole day in Gambela (a rural center). The availability and distribution of human resources has direct implications for the issue of time as a resource. It was discussed by participants that HCWs may become overworked, making it difficult for them to act in accordance with the PPH guideline recommendations.

Participants also noted that certain aspects of the guideline recommendations are dependent not only on the availability of HCWs, but on the availability of skilled HCWs including:

- In settings where skilled birth attendants are available, controlled cord traction is practiced.
- The frequent reassessment required with oxytocin is a challenge due to a lack of skilled HCWs and their time available to complete regular assessments.
- Uterine massage is an important intervention for the prevention and treatment of PPH, however, some centers may not have enough skilled staff available to implement this practice. In these facilities, it is difficult for the limited number of skilled attendants to conduct uterine massage while also managing other emergencies, so uterine massage may become a back-up as opposed to a top priority.

The participants discussed current efforts to increase human resources in Ethiopia as a facilitator to implementing the guideline recommendations. The process has started and they noted that recruitment and training of HCWs is an ongoing process. The national target is to produce a total 18,000 HCWs over the next 5 years, 3,000 of which would be physicians and 300 of which would be obstetricians and surgeons.

Facilities
Participants discussed the need for a national strategy to address issues related to infrastructure, including lack of facilities, inadequate operating conditions of existing facilities (e.g., lack of electricity and communication channels) and poor road conditions (e.g., due to drought experienced during the rainy season, which can cause roads to flood and be inaccessible). Another issue that was identified was unequal access to health centers across geographic areas. Therefore, it was identified as a desired facilitator to increase the number of health care facilities. For example, participants from Gambela stated that none of the health centers in that region are functional and that there is only one hospital, which is overcrowded with patients. The participants considered this to be a significant barrier to implementing guideline recommendations.

Drug procurement, distribution, management
The issue of drug procurement, distribution, and management was discussed by participants as affecting the implementation of the PPH guideline recommendations. Pharmaceutical management to regulate drug supply nationally as well as at the level of health care facilities was cited as a prominent challenge in Ethiopia. Participants described a drug distribution system in which medication is not adequately supplied

to all health centers. This challenge at the system level can lead to a lack of readily available supplies and stock outs. Participants noted that the problems related to drug stock outs most significantly affect rural centers where the majority of the population (84%) resides.

Improved supply chain management and distribution practices at the system level were considered to be an important facilitator for ensuring an adequate supply of oxytocin among other drugs to be readily available at the health facility level. More specifically, participants suggested that in order to rectify the issue of stock outs, timely ordering practices, procurement, and adequate storage of drugs is necessary as well as a comprehensive monitoring strategy for drug supplies to be implemented at the facility level.

Data collection & monitoring

Guideline implementation challenges raised by participants related to data and monitoring included: a lack of routine collection and reporting of data on maternal deaths; and a lack of facility-level audit and feedback for health care provider performance. Participants in both FGs discussed that many maternal deaths occur at home or in the community and that the cause of these deaths and associated factors are often not reported. This creates a barrier to accurately measuring how many women are dying from complications such as PPH, and how PPH is being managed across the country. Consequently, this lack of data contributes to challenges with informed decision-making at the level of the health care system and the health care provider in terms of what implementation strategies are needed and to what degree. Communication between the community and hospitals/health care centers regarding at-home births was identified as a desired facilitator. Issues were also raised regarding current data collection practices in health care centers related to maternal deaths. Specifically, it was stated that there are opportunities for improvement including the introduction of more rigorous documentation and monitoring practices as well as increased quality indicators and standardization of how data is captured.

Facility-level audit and feedback strategies related to HCW practices were also discussed. Participants identified onsite/facility-based supervision as a desired facilitator. For example, it was suggested that a community leader or head nurse could be identified to supervise selected deliveries with each MW and/or physician at a given facility and provide feedback on the observed practices as they comply with the guideline recommendation. This would help to ensure HCW practices are being monitored and are in compliance with the guidelines.

Policies & incentives

A common theme described by participants in both FGs was the prominent role played by policies and incentives to influence HCW practice and ultimately, implementation of the guideline recommendations. Low motivation among HCWs due to a lack of competitive benefits packages was raised as a key barrier to guideline implementation. Participants perceived that an increase in the benefits package offered to HCWs can renew their commitment and increase buy-in around efforts to improve guideline uptake.

Participants perceived that policies supporting the redistribution of HCWs across the country (therefore alleviating the current unequal distribution, particularly in rural health centers) are warranted. Increasing incentives, or offering a higher benefits package, for those to relocate their families and live and work in rural areas was discussed by participants as an action that could address the issue of HCW distribution. Participants did acknowledge the FMoH policy that mandates two MWs per health care facility, and complementary efforts to increase human resources in Ethiopia, as facilitators currently in progress. Other barriers discussed related to policies and incentives included the absence of a national policy to mandate the adoption of a standard procedure/protocol related to the WHO guidelines, and challenges

experienced with the health extension worker program not functioning optimally in certain regions of the country.

Finally, participants described a number of system level facilitators that are planned or currently in place in the area of policies and incentives including the following initiatives: the current national policy that supports administration of misoprostol; the policy directive around the requirement of continuing medical education credits for license renewal of HCWs as issued by the Food, Medicine, and Drug Administration Authority (effective since September 2014); the pilot implementation of a Health Information System to electronically manage the ordering of supplies and drugs and to provide timely information and data to inform decision-making related to supply chain management; and efforts related to environmental/social planning including plans to produce 13 more universities (with medical schools offered in the majority of the new universities) as well as a new medical education system to produce more physicians in different hospitals.

Readiness for change

Participants expressed opposing views with regards to the readiness of the system to change. At the Ministry level, the readiness for change related to guideline implementation was perceived to be high. However, at the provider level, some participants perceived resistance to change (i.e., lack of buy-in, low morale), while others disagreed indicating that providers would recognize the importance of the issue and will support change regardless of frustrations related to low morale. The participants also expressed the importance of a high degree of readiness at the facility level to ensure the guidelines are being implemented appropriately.

The readiness of HCWs to deliver services related to PPH prevention and management was also discussed. Some participants expressed that individuals at the Ministry level and those from urban health centers who have experience in implementing other provincial guidelines could offer guidance and support on how best to implement the recommendations in the PPH guideline in other settings or facilities where implementation is comparatively poor. Participants also discussed lack of HCW motivation as a barrier to guideline implementation and a factor that would reduce their level of readiness for change. For example, some HCWs are dissatisfied and demotivated, especially in remote areas, and this may be a barrier to implementation. It was the opinion of the participants that the guideline will be accepted (e.g., in terms of quality and relevance) but that implementation will be difficult because of this lack of motivation and job satisfaction.

Guidelines & protocols

The availability of guidelines and protocols was discussed as a key determinant of guideline implementation in Ethiopian health facilities. Participants identified a current implementation gap extending from policy to practice. The use of misoprostol was cited as an example of a policy that should be implemented widely but is not. Participants from both FGs cited lack of accessible, visible protocols in health facilities, especially in small centers, as a barrier to implementation of the guideline recommendations. Desired facilitators or opportunities to improve this issue were also discussed. Firstly, uniform protocols based on the guidelines could be created and mandated for use across the country. For example, a national obstetric protocol for health centers and hospitals exists which is currently being distributed to all health facilities in Ethiopia. Secondly, it was felt that these standards/guidelines need to be communicated to HCWs in an effective manner. It is important that providers have knowledge of these guidelines and disseminate it to fellow HCWs whether via training sessions, mentorship programs, or sharing of materials obtained at workshops. Lastly, the protocols must be distributed from the national level to the facility level for standard access and use across the country. The need to adapt guidelines to

different health care settings to account for regional variations in practice was also cited by participants as an important factor in influencing guideline implementation.

Summary of Key Barriers and Facilitators – Health Care System Level
**Barriers**
• Lack of refrigeration equipment and electricity required to store oxytocin in some health centers.<br>• Lack of blood supply at rural health centers.<br>• Lack of access to blood transfusion facilities and intensive care units.<br>• Lack of recruitment and distribution of skilled HCWs nationally.<br>• Lack and poor distribution of functional health centers in rural areas.<br>• Inadequate supply and distribution of medications leading to stock outs.<br>• Lack of data collection and communication regarding maternal deaths happening in communities across the country.<br>• Low motivation of HCWs as related to a lack of competitive benefits packages.<br>• Lack of national policy to mandate the adoption of a standard procedure related to the WHO guidelines.<br>• Lack of visible and available guidelines and protocols in health centers.
**Facilitators**
• Readily available supply of IV antibiotics and fluids in both rural and urban health centers.<br>• Current efforts to increase human resources in Ethiopia.<br>• A system to regulate timely ordering practices, procurement, and adequate storage of drugs is needed.<br>• Introduce more rigorous and standardized documentation and monitoring practices for data collection.<br>• Onsite, facility-based supervision is necessary to ensure HCW guideline compliance.<br>• Distribution of standardized and mandated protocols nationwide.<br>• Dissemination of guidelines via training sessions, mentorship programs, or sharing of materials obtained at workshop to ensure uptake of guideline information.

Health Care Provider Level

Factors were identified at the provider level that can influence implementation of the WHO guideline recommendations. Factors in the following categories were described by participants: beliefs, attitudes, buy in; knowledge and skills; training and supportive supervision; and role definition.

Beliefs, attitudes, buy-in

The beliefs and attitudes held by HCWs and their level of buy-in were identified as important factors in determining the acceptance and implementation of the PPH guideline in the Ethiopian context. Some participants indicated that provider resistance to using available guidelines and lack of adherence to recommendations is an issue. For example, senior HCWs may be resistant to professional development efforts or having their practice challenged. Conversely, other participants indicated that acceptance of the guideline would not be an issue. An additional barrier stated was the general motivation and satisfaction of HCWs across the country, which was currently felt to be low. The participants indicated that since the morale of health care workers (including physicians, MWs, and nurses) was low, they are less likely to buy-in and incorporate the guideline recommendations into their practice.

Knowledge & skills

The knowledge and skills of the HCWs were identified as key factors that can influence the uptake and implementation of the guideline recommendations. One of the main challenges discussed in both FGs

was the shortage of skilled providers/attendants in Ethiopia, especially in rural health facilities. For example, skilled providers are not always available to perform bimanual compression, one of the guideline recommendations. Another issue that was raised was that of staff trained in PPH management and the need for improvement of skills. The consensus was that there is a general lack of knowledge and of skills for certain procedures vital to management of PPH (e.g., controlled cord traction). Participants discussed the necessary knowledge required to diagnose PPH as well as recognize the importance of different interventions to manage PPH. It was noted that simulation exercises/drills would be beneficial in increasing health care professionals' response time to PPH, especially since PPH is not an "everyday occurrence". Participants reported that providers can lose the knowledge gained in training if they do not practice these skills regularly.

Training & supportive supervision

Participants identified training, supportive supervision, coaching, and mentorship as key activities that may facilitate implementation of guideline recommendations and ensure that providers are practicing according to the guidelines/standards. For example, participants felt that supervisors, coaches, and mentors should be available to support providers if they have any doubts or concerns with respect to managing PPH. It was considered important that trainers/supervisors be able to teach, encourage, and help providers solve problems in addition to imparting the necessary skills.

In particular, participants indicated a need for onsite/bedside training and feedback in order to aid providers in identifying what they did well and what areas of their PPH management strategies could use improvement. The current method of supervision from the district head office or regional bureau was discussed. Participants indicated that individuals providing the training/supervision may not have clinical experience but are required to supervise MWs and/or physicians. For example, a current issue is that some trainers lack practical skills needed to demonstrate certain techniques and are therefore not the most appropriate individuals to lead training sessions for frontline staff. Participants suggested a model for supervision that included instructors that are senior hospital staff (e.g., senior MWs) who have both the practical and theoretical knowledge and skills needed to effectively provide training.

Participants also discussed the lack of knowledge exchange opportunities between senior health care providers and newly recruited/trained providers. As a result, it was felt that evidence-based information is currently not being transferred between health care providers. The use of simulation for training employees and students in PPH management was a common suggestion in both FGs. It was specified that the trainer should be equipped with theoretical as well as practical skills to conduct the simulation and transfer their knowledge. A need for senior MWs to share their skills with younger, newer employees and students was expressed. Another area discussed was the lack of regular quality improvement and professional development activities (e.g., continuous training). Participants emphasized the importance of continuing medical education and its relevance to MWs, obstetricians, and physicians to evaluate their quality of care with regards to prevention and management of PPH.

Role Definition

Positive interprofessional dynamics and an understanding of one's role were discussed as factors that may facilitate guideline implementation at the provider level. One issue discussed by participants was that task-shifting, or role re-definition, may impact the quality of the service provided. Prevention and treatment of PPH directly involves various cadres of health care professionals (e.g., MWs, gynecologists, nurses) whose role is management of these conditions. However, it is most often the MWs who directly provide maternal and perinatal care. With regards to role re-definition and task shifting, it was also noted that nurses can administer IV fluids in health facilities if a MW is not present and misoprostol can be administered by other HCWs as needed.

Summary of Key Barriers and Facilitators – Health Care Provider Level
**Barriers**
• Provider resistance to guideline use and lack of adherence to recommendations.<br>• Low motivation/morale of HCWs nationwide.<br>• Shortage of skilled HCWs/birth attendants, especially in rural health centers.<br>• Lack of knowledge and of skills for certain procedures vital to management of PPH.<br>• Lack of regular quality improvement and professional development activities (e.g., continuous training).<br>• Lack of knowledge exchange opportunities between senior health care providers and newly recruited/trained providers.
**Facilitators**
• Simulation exercises/drills to increase HCWs' response time to PPH.<br>• Onsite/bedside training and feedback to aid HCWs in strategies related to PPH management.<br>• Individuals that have both the practical and theoretical knowledge and skills to effectively provide training.

Pregnant Women and the Community Level

Factors were identified at the level of pregnant women and the community that can influence implementation of the WHO guideline recommendations, as perceived by workshop participants. Factors in the following categories were described by participants: traditional beliefs; knowledge and awareness; and access to health care services.

Traditional beliefs

The role of traditional cultural beliefs was identified as a pervasive community level factor that can influence health care-seeking behaviours. It was identified by both FGs that the majority of women in Ethiopia give birth at home (quoted as greater than 70% of the population in one FG). A number of underlying factors contribute to this common occurrence including preferences and beliefs of the patient and/or her family members and misconceptions or fear of health care facilities and providers. For example, the traditional belief that women should give birth near the water has led to higher numbers of births in community settings. The strong cultural practice of home births was perceived as a prominent challenge to preventing and managing PPH as the majority of PPH cases occur when women gave birth at home and were unattended by a skilled HCW. It may then be too late for HCWs to treat PPH by the time the patient arrives at the health care facility. For example, mothers may come to the health care facility with PPH caused by atony, or retained placenta, but may seek health care at a stage when HCWs are no longer able to effectively treat the patient.

Knowledge & awareness

Knowledge and awareness of the individual woman and her family was identified as a prevalent factor influencing the patients' treatment decision-making. It was stated in both FGs that the majority of births are occurring outside of health facilities and are thus not attended by skilled providers. It was further noted that mothers and families make treatment decisions (e.g., where to deliver, uterine massage) based on their knowledge. Therefore, if mothers are not aware of the significant potential risks of delivering at home and/or the benefits of delivering in a health care facility they are less likely to make arrangements to go to a health center when it is time to deliver. Participants identified the need to educate the community about the importance of delivering babies at health centers as a potential facilitator.

Another common challenge that arose in both FGs was that of conducting uterine massage to reduce the risk of bleeding due to PPH. According to the participants, HCWs often encounter barriers when encouraging mothers to use uterine massage for a number of reasons. Firstly, the mother may experience pain during uterine massage and refuse to continue. Secondly, family members may refuse to massage the mother because they realize that she is in pain. However, the consensus was that it is important to educate both the mother and family members on the benefits of uterine massage and its ability to reduce the risk of bleeding. Furthermore, FG participants emphasized the importance of patient education on the benefits of self-massage as a facilitator to PPH prevention and management.

Access to health care services

Access to health care services was described as a significant barrier to improving patients' health care-seeking behaviours. Participants identified that even if a patient decides to go to a health care center for delivery, there are barriers including poor infrastructure, lack of transportation (e.g., lack of vehicle), lack of funds to pay for transportation, and poor road conditions (especially during floods) that may impede travel to, and ultimately access to, the health center. This is especially important in an emergency condition, such as PPH, when a patient is urgently in need of a skilled provider. Furthermore, the time it takes to travel to the health center, especially with poor road conditions, can further jeopardize the patient in an emergency situation.

Summary of Key Barriers and Facilitators – Pregnant Women and the Community level
**Barriers**
• Traditional beliefs and common practices leading to home births as opposed to health center births.<br>• Lack of knowledge and awareness of potential risks of delivering at home and/or the benefits of delivering in a health center.<br>• Pain and discomfort related to uterine massage practices.<br>• Challenges in accessing health care services and center (e.g., lack of vehicle, funds to pay for transportation, poor road conditions).
**Facilitators**
• Education of community members about the importance of delivering babies at health centers.<br>• Education of the mother and family members on the benefits of self-massage to prevent and manage PPH.

Ranking Exercise

On Day Two of the in-person workshop, participants were asked to rank the feasibility of the 11 recommendations from the Prevention and Treatment of PPH guideline that were deemed to be top priorities in Ethiopia based on FG findings and deliberations among the workshop facilitators and in-country experts (Day One). Tasks ranked as most feasible were described by workshop participants to be the easiest to operationalize. Those ranked as least feasible were recommendations that were described as complicated, introduced safety concerns, or were in direct opposition with current policies or practices. Five of the 11 recommendations were re-ranked due to disparate responses. All results are presented in **Appendix D**. The following five recommendations were deemed to be most feasible to implement in the Ethiopian context, each receiving a median score of 9 out of 9 ("extremely feasible"):

- The use of uterotonics for the prevention of PPH during the third stage of labour is recommended for all births. (Strong, moderate)
- Late cord clamping (performed after 1 to 3 minutes after birth) is recommended for all births while initiating simultaneous essential newborn care.(Strong, moderate)

- Postpartum abdominal uterine tonus assessment for early identification of uterine atony is recommended for all women. (Strong, very low)
- Uterine massage is recommended for the treatment of PPH. (Strong, very low)
- The use of uterine packing is not recommended for the treatment of PPH due to uterine atony after vaginal birth. (Weak, very low)

Potential Implementation Strategies to Inform a Country-Specific Implementation Plan

On Day 2, participants worked in small groups to consider implementation strategies and activities that could address barriers to implementation of the 11 priority recommendations selected in the ranking exercise. Each small group focused on specific barriers as determined by the group and discussed potential implementation strategies accordingly. **Table 3** provides a summary of the implementation strategies identified by workshop participants as well as those recommended by the study team. Due to time constraints, participants from each FG were only able to identify potential implementation strategies for some of the recommendations and related barriers, thus study team recommendations are also included. It should be noted that study team recommendations were developed by the SMH study team at the time of data analysis based on the pre-workshop survey and workshop findings. The SMH study team recommendations provide additional options of potential implementation strategies that in-country stakeholders may consider when moving forward with next steps.

Table 3. Recommended strategies/activities to address perceived barriers to implementing guideline recommendations

Level of barrier	Category of barrier	Recommended implementation strategies/activities
Health care system	Access to drugs, equipment, supplies	Recommended by Study Team<br>• Secure formulation of oxytocin that can be stored at room temperature to decrease number of cases where the drug becomes compromised due to improper storage.<br>• Maintain a cold chain of oxytocin storage at all levels, where oxytocin is kept between 2 and 8 degrees (Celsius) to prevent degradation of the drug and avoid future drug waste.
	Access to human resources	Recommended by Participants<br>• Design and implement graduate level programs (e.g., a Master degree) in midwifery and clinical midwifery as a strategy to produce and retain more MWs.<br>• Re-distribute HCWs internally (within institutions) and externally (across institutions) to concentrate on maternal and newborn health services. To do this, all HCWs should be sufficiently trained to work in different clinical areas as required.<br>• Support the current initiative at the national level to create more health care providers, but emphasize that the quality of health care providers is as important as the quantity. Quality can be ensured through improved training and supervision programs for HCWs at all levels.<br>• Conduct a review/needs assessment of the Health Extension Worker Program in rural regions to increase the number of HCWs and identify opportunities to improve functioning of the program in these regions.<br>• Improve quality of education received by individuals before they are admitted to university programs (e.g., Midwifery), especially in rural areas, to make sure they are able to receive training and practice at the appropriate level.<br>• Improve leadership programs to retain HCWs in rural, underserviced areas.<br>• Encourage government transparency with HCWs stating where they will be placed after their training, the length of time they will be working there, and the options they will have once that time is completed as a retention strategy for HCWs in rural areas.<br>• Create a specific focus for the education program for HCWs so that the increase in the number of students does not mean a compromise in the quality of their education and the quality of provider knowledge and skills. This would require facility expansion proportionally to the number of students accepted.<br>Recommended by Study Team<br>• Promote teamwork and shared responsibilities of HCWs within institutions. All facility staff should be able to work in the labour ward if needed and should be trained to do so.
	Access to facilities	Recommended by Participants<br>• With a shift to patient-centered receipt of funding, facilities will need to be designed to attract more patients and should be conducive to patient needs. For example, cleanliness of facility, welcoming staff, and incorporation of a traditional coffee ceremony.<br>Recommended by Study Team<br>• Ensure that facilities have refrigerators to store oxytocin, or change facility policies to ensure that refrigerators used to store vaccines can also accommodate drugs like oxytocin.
	Drug procurement, distribution, management	Recommended by Study Team<br>• Require that request and reporting of drugs is completed on time to minimize stock-outs.<br>• If stock-outs occur, implement a cost-sharing program between facility and government (e.g., facility absorbs partial cost of drugs to buy locally from private vendors until stock is replenished)<br>• Develop protocols, secure necessary equipment, and designate staff roles to monitor and record drug orders and quality control at (1) the level of the health care facility; and (2) at the national level to ensure drug orders are being delivered and to inform national drug ordering practices.<br>• Scale up implementation of the Health Information System to electronically manage the ordering and distribution of supplies across regions.
	Data collection & monitoring	Recommended by Study Team<br>• Develop clinical indicators that will enable systematic and standardized monitoring of conditions and clinical practice (e.g., number of women receiving uterotonics)<br>• Use tracer drugs to monitor stocks of essential drugs (e.g., oxytocin)
	Policies &	Recommended by Participants

Level of barrier	Category of barrier	Recommended implementation strategies/activities
	incentives	• Recognize the important role of MWs by offering a benefit package consisting of improved salary, duty hour payment, and better living arrangements. Standardize this benefit package across all regions.<br>• Create competitive salaries and modern infrastructure (electricity, housing) in rural/remote communities to incentivize HCWs to work in underrepresented areas.<br>• Promote transparency of the FMoH in outlining benefit packages for HCWs based in different regions.<br>• Create policies around holding medical schools to a certain standard of education to ensure the quality of physicians produced is high.<br><u>Recommended by Study Team</u><br>• Incorporate into policies an increased role for nurses and MWs in terms of consulting and decision-making for patient care.
	Guidelines & protocols	<u>Recommended by Study Team</u><br>• Engage stakeholders (professional associations, front line clinicians) prior to the rollout of the guideline, to enable them to comment on the guideline prior to dissemination.<br>• Develop standard protocols based on the guidelines at higher levels (Ministry/regional/district) and distribute to facilities for onsite guidance. Protocols should be user-friendly, ready-to-use, and visible (e.g., posted on wards) to act as reminders for health care workers.
Health care Provider	Beliefs, attitudes, buy-in	<u>Recommended by Study Team</u><br>• Create an organizational culture that promotes and supports accountability to professional standards and guidelines (e.g., if a physician frequently asks a MW for reports on a patient's partograph, this encourages the MW to use the partograph routinely).
	Knowledge & skills	<u>Recommended by Study Team</u><br>• Develop protocols based on the WHO guideline that are user-friendly, ready-to-use, and visible (e.g., posted on wards) to act as reminders for HCWs.<br>• Provide guideline-relevant training (see below) to HCWs, and adapt policies to empower MWs to approach physicians when a woman's health is at risk and to be part of the decision-making process.
	Training & supportive supervision	<u>Recommended by Participants</u><br>• Establish mentorship programs between more experienced and less experienced HCWs.<br>• Implement more supportive supervision at the facility level, and cascade supervision within districts<br>• Strengthen orientation (pre-service), in-service training, and professional development (e.g., continuing medical education) opportunities for all professions, including physicians, nurses, and MWs. Training should have a hands-on component to help prepare HCWs for situations that they may encounter infrequently in their practice.<br>• Train HCWs on appropriate, effective, and transparent communication with patients, and on the rights of the patients so that women do not feel ill-treated when coming to the labour wards<br>• Ensure that school curricula for HCWs are updated to include the most recent clinical guidelines.<br><u>Recommended by Study Team</u><br>• Consider the practice of placing HCWs on a "probation" period after pre-service training that enables supervisors to assess competencies and skills prior to sending staff to other facilities for work.
	Role definition	<u>Recommended by Participants</u><br>• Government recognition of importance of MWs role in maternal health care to improve the current lack of respect that is experienced by some MWs by other cadres of HCWs. This could improve interprofessional dynamics and collaboration between HCWs.<br>• Promote interprofessional collaboration and teamwork, so that all professionals support one another and are recognized for good work such as establishing interdisciplinary working groups in health facilities (e.g., an interdisciplinary quality improvement team).<br>• Sensitize senior management to the issues faced by front line workers through enhanced communication, so that they are aware of issues and are more willing to constructively support staff<br><u>Recommended by Study Team</u><br>• Promote interprofessional training, to enhance collaboration and role recognition across the health care team.
Pregnant women and the	Traditional beliefs	<u>Recommended by Participants</u><br>• Provide health education to pregnant women and communities via the health extension workers, religious leaders, and/or other government figures at the regional or town level about the misconceptions of some cultural beliefs that may act as barriers to women receiving health care.

Level of barrier	Category of barrier	Recommended implementation strategies/activities
community		• Make the health facility environment more welcoming (e.g., friendly staff, clean and safe environment) to pregnant women to make them feel more comfortable and to attract them to facilities.<br>• Quality improvement committee in health facilities consisting of MWs, laboratory professionals, pharmacy workers and clinical nurses focused on ensure the facility is clean and safe and thus appeals to mothers.<br>• Advocate for the importance of the role of nurses and midwives, so that pregnant women feel comfortable in their care and will not blame these health care providers for negative outcomes<br><u>Recommended by Study Team</u><br>• Consider scale up telephone/SMS reminders program to encourage women to attend antenatal care<br>• Use mass media to promote awareness of the benefits of the recommendations<br>• Use of community plays, posters, or talks held in the community or in the waiting areas of health centers.
	Knowledge & awareness	<u>Recommended by Participants</u><br>• Provide health education to pregnant women to encourage them to go to health facilities to deliver.<br>• Increase awareness among women and the community that care is provided to mothers for free in public health facilities, including antenatal, postnatal, and family planning.
	Access to health care services	<u>Recommended by Participants</u><br>• Scale up of the Maternity Waiting Home initiative, which is currently being used in some remote areas to mitigate barriers experienced with the transportation to health facilities for deliveries during the rainy season.<br><u>Recommended by Study Team</u><br>• Promote linkage of services between facilities. Women can be linked to clinical postnatal care by community leaders.<br>• Consider using telemedicine (i.e., the remote diagnosis and treatment of patients by means of telecommunications technology) as a tool to link lower level and higher level facilities.<br>• Provide adequate transport services for transfer of women between facilities.

LIMITATIONS

There are four main limitations to this research. First, data from workshop activities were collected from a small sample of participants. This sample may not be representative of the entire population working in the maternal health sector of Ethiopia. We acknowledge that implementation of national level guidelines requires participation at all levels and from diverse cadres of health care system stakeholders, and therefore steps were taken to ensure that there was representation from major stakeholder groups from different geographic regions and diverse professional backgrounds across the country. Second, the MWs focus group discussions were conducted in Amharic and a translator was required. This language barrier could have led to issues in interpretation or points being missed during the discussions. Third, project organizers conducting this activity faced time, resource, and space restrictions. To mitigate these restrictions, a purposeful convenience sample was used to identify stakeholders to participate in the pre-workshop survey and in-person workshop. Finally, the understanding and interpretation of the data was limited by cultural barriers and local contextual factors. To reduce the impact of this limitation, in-country experts were consulted throughout the process to enhance comprehension of the data and its relevance to the local context.

RECOMMENDATIONS AND CONCLUSION

The process of selecting priority maternal and perinatal health recommendations and exploring barriers and facilitators to implementation of the WHO guideline on Prevention and Treatment of PPH yielded valuable information to inform implementation planning in Ethiopia. The findings of the pre-workshop survey aligned with those of the in-person workshop, with the workshop providing an opportunity to explore perceptions and priorities in greater depth. Both data collection methods helped to inform concrete recommendations for moving forward in facilitating the implementation of priority guideline recommendations in the local context. Specifically, based on the findings that emerged across the pre-workshop and workshop activities, ten recommendations have been developed to guide next steps.

- **Recommendation #1**: Create a guideline implementation working group (WG) as a sub-group of the Federal Ministry of Health's maternal health case team. This guideline implementation WG should be multi-disciplinary and include representation from multiple levels.

Note: the intention is not for the guideline implementation WG to fulfill all ten of these recommendations on their own, but rather to play a key role in championing these initiatives and building strategic partnerships across sectors and at multiple levels as needed to move guideline implementation efforts forward in Ethiopia.

The following six recommendations are intended for operationalization by the guideline implementation WG:

- **Recommendation #2**: Adapt the WHO maternal health guideline on Prevention and Treatment of PPH for the Ethiopian context using the ADAPTE process.
- **Recommendation #3**: Create standard protocols on how to implement the guideline recommendations and distribute to facilities for onsite guidance. Protocols should be user-friendly, ready-to-use, and visible (e.g., posted on wards) to act as reminders for HCWs.
- **Recommendation #4**: Select and implement priority clinical indicators as part of a monitoring and evaluation strategy on PPH prevention and management to enable systematic and standardized assessment of guideline implementation.

- **Recommendation #5**: Establish a mentorship program at the facility level between junior and senior HCWs to provide technical support and supportive supervision on implementation of the guideline recommendations protocols.
- **Recommendation #6**: Establish an interdisciplinary quality improvement team (e.g., including physicians, midwives, administrators) at each health care facility to identify priority areas for practice improvement based on the clinical indicators identified in recommendation #4. Quality improvement teams should develop and monitor quality improvement strategies for the priority areas at the facility level.
- **Recommendation #7**: Design and conduct a process and outcome evaluation of the guideline implementation approach.

The following three recommendations are intended for the guideline implementation WG in partnership with other key stakeholders (e.g., professional associations, other ministries, independent evaluators, etc.):

- **Recommendation #8:** Identify strategies to improve and standardize the benefits package offered to HCWs across all regions so that HCWs in rural regions receive the same compensation package as HCWs in urban regions.
- **Recommendation #9**: Conduct a process evaluation of the Health Extension Worker Program to improve functioning of the program in regions where it is not optimally working and share lessons learned from those regions where the program is working.
- **Recommendation #10**: Evaluate the Maternity Waiting Home initiative, which is currently being used in some remote areas to mitigate barriers experienced with the transportation to health facilities for deliveries during the rainy season. Key evaluation outcomes to consider could include: increased number of women giving birth at health care facilities, decreased incidence of PPH cases, decreased incidence of maternal deaths from PPH cases). If successful outcomes are demonstrated, consider scale up of this program.

The methods used to inform the implementation strategies discussed in this report are transferable to other priority areas and other guidelines, particularly those in the area of maternal and perinatal health. Many of the barriers, facilitators, and recommended implementation strategies identified regarding the WHO guideline on Prevention and Treatment of PPH are applicable to other priority areas in health care; therefore, these findings can inform and be integrated into future barrier and facilitator assessments and guideline implementation planning initiatives in Ethiopia.

REFERENCES

1. McGlynn EA, *et al*.: **The quality of health care delivered to adults in the US**. *N Engl J Med* 2003, 348:2635-2645.

2. Davis D, Evans M, Jadad A, Perrier L, Rath D, Ryan D, Sibbald G, *et al*.: **The case for KT: shortening the journey from evidence to effect.** *BMJ* 2003, **327**(7405)**:**33-35.

3. Madon T, Hofman KJ, Kupfer L, Glass RI: **Public health. Implementation science.** *Science* 2007, **318**(5857)**:**1728-1729.

4. Shah BR, Mamdani M, Jaakkimainen L, Hux JE: **Risk modification for diabetic patients. Are other risk factors treated as diligently as glycemia?** Can J ClinPharmacol 2004; **11**(2):e239-e244.

5. Pimlott NJ, Hux JE, Wilson LM, Kahan M, Li C, Rosser WW: **Educating physicians to reduce benzodiazepine use by elderly patients: a randomized controlled trial**. CMAJ 2003; **168**(7):835-839.

6. Kennedy J, Quan H, Ghali WA, Feasby TE: **Variations in rates of appropriate and inappropriate carotid endarterectomy for stroke prevention in 4 Canadian provinces**. CMAJ 2004; **171**(5):455- 459.

7. World Health Organization: World Health Statistics 2014 Part I: Health-related Millennium Development Goals. http://www.who.int/gho/publications/world_health_statistics/EN_WHS2014_Part1.pdf?ua=1 Accessed 08 July 2015.

8. Gebrehiwot Y, Tewolde BT. **Improving maternity care in Ethiopia through facility based review of maternal deaths and near misses.** International Journal of Gynecology and Obstetrics 2014; **127**:S29-S34.

9. Hadis M, Dibaba A, Ababor S, Assefa Y. **Improving skilled birth attendance in Ethiopia (SURE policy brief)**. Addis Ababa Ethiopia: Ethiopian Public Health Institute, 2014. www.evipnet.org/sure

10. **Ethiopia Mini Demographic Health Survey 2014**. Adis Ababa Ethiopia: Central Statistical Agency, August 2014. http://www.unicef.org/ethiopia/Mini_DHS_2014__Final_Report.pdf

11. Haines A, Kuruvilla S, Borchert M: **Bridging the implementation gap between knowledge and action for health**. Bull World Health Organ 2004, **82**(10):724- 731.

12. World Health Organization: **WHO recommendations for the prevention and treatment of postpartum haemorrhage 2012**. http://www.who.int/reproductivehealth/publications/maternal_perinatal_health/9789241548502/en/ Accessed 12 Sep 2014.

13. Jones J, Hunter D: **Consensus methods for medical and health services research.** *BMJ* 1995, **311:**376-380.

14. Fitch K, *et al*.: **The RAND/UCLA appropriateness method user's manual**. RAND Corporation; 2001.

15. Braun V, Clarke V: **Using thematic analysis in psychology**. Qual Res in Psychol 2006; **3**(2): 77-101.

APPENDIX A: PRE-WORKSHOP SURVEY

GREAT Project Assessment Survey - Ethiopia

Introduction

Welcome to the GREAT Project (Guideline-driven, Research priorities, Evidence synthesis, Application of evidence, and Transfer of knowledge). The purpose of the project is to improve the quality of care for mothers and infants in Ethiopia; to build capacity locally; and to inform the development of a tailored strategy to implement the following World Health Organization (WHO) guideline on maternal and perinatal health:

Prevention and Treatment of Post-Partum Haemorrhage (PPH) (2012)
[Please see Appendix A: PPH Guideline Summary]

You are being invited to participate in a short survey to help the project team better understand the key priorities related to the WHO's Prevention and Treatment of PPH guideline in the Ethiopian context. Ultimately, your responses will be used to help inform the development of a strategy for adapting and implementing the Prevention and Treatment of PPH guideline in Ethiopia. Participation in the survey will take approximately 10- 15 minutes of your time. Survey responses are anonymous and will inform the proceedings of a two-day in-person workshop to be held in Ethiopia in May 2015.

By completing and submitting this survey, your consent to participate is implied.

Please complete the survey by **April 24th 2015.**

If you have any questions about the survey, please contact one of the following individuals:

Dr. Luwam Teshome (WHO, Ethiopia) - Email: teshomel@who.int, **Phone#** +251-911663707
Dr. Azmach Hadush (Ministry of Health, Ethiopia) - gebregiorgisaz@who.int

Thank you very much for your time and participation.

Section 1: Demographic Information

1. In which region/zone/district do you work? Please respond (if applicable) in the boxes provided below.

Region: ____________________

Zone: ____________________

District: ____________________

2. At what level of the health care system do you work? Please check all responses that apply.

Specialized Hospital	☐	District Health Office	☐
General Hospital	☐	Non-governmental Organization	☐
District Hospital	☐	International Organization	☐
Health center	☐	Professional Regulatory Body	☐
Regional Referral Hospital	☐	Private Hospital	☐
Ministry of Health	☐	Non-Governmental organization supported clinic	☐
Regional Health Bureau	☐	Professional Association	☐
Other (Please specify in Question 3)	☐		

3. What is your title/role description?

4. How long have you been in this role? (please check only one box)

- ○ Less than 1 year
- ○ 1-2 years
- ○ 3-5 years
- ○ 6-10 years
- ○ 11-20 years
- ○ More than 20 years

Section 2: Prioritization of Clinical Areas in the Prevention and Treatment of PPH Guideline

In this section, you are being asked to prioritize recommendations of the Prevention and Treatment of PPH Guideline.

In Table 1, the recommendations of the WHO guideline on Prevention and Treatment of PPH (2012) have been grouped according to the following four clinical areas:

- *Use of uterotonics;*
- *Cord clamping;*
- *Uterine massage; and*
- *Protocol/training.*

The strength and quality of the recommendation are provided in brackets (Strength, Quality).

Table 1. PPH clinical guideline areas and related recommendations

**Clinical**	**PPH recommendations related to this clinical area**
Use of Uterotonics	• Oxytocin (10 IU, IV/IM) is the recommended uterotonic drug for the prevention of PPH (Strong, Moderate)<br>• In settings where oxytocin is unavailable, the use of other injectable uterotonics (if appropriate ergometrine/ methylergometrine or the fixed drug combination of oxytocin and ergometrine) or oral misoprostol (600 µg) is recommended. (Strong, Moderate)<br>• In settings where skilled birth attendants are not present and oxytocin is unavailable, the administration of misoprostol (600 µg PO) by community health care workers and lay health workers is recommended for the prevention of PPH.(Strong, Moderate)<br>• The use of uterotonics for the prevention of PPH during the third stage of labour is recommended for all births (Strong, Moderate)<br>• Oxytocin (10 IU, IV/IM) is the recommended uterotonic drug for the prevention of PPH (Strong, Moderate)<br>• In settings where oxytocin is unavailable, the use of other injectable uterotonics (if appropriate ergometrine/ methylergometrine or the fixed drug combination of oxytocin and ergometrine) or oral misoprostol (600 µg) is recommended. (Strong, Moderate)<br>• In settings where skilled birth attendants are not present and oxytocin is unavailable, the administration of misoprostol (600 µg PO) by community health care workers and lay health workers is recommended for the prevention of PPH.(Strong, Moderate)<br>• Oxytocin (IV or IM) is the recommended uterotonic drug for the prevention of PPH in caesarean section (Strong, Moderate)<br>• Intravenous oxytocin alone is the recommended uterotonic drug for the treatment of PPH (Strong, Moderate)

	• Monitoring the use of uterotonics after birth for the prevention of PPH is recommended as a process indicator for programmatic evaluation (Weak, very low)<br>• A single dose of antibiotics (ampicillin or first-generation cephalosporin) is recommended if manual removal of the placenta is practised. (Weak, very low)
Cord Clamping	• In settings where skilled birth attendants are available, CCT is recommended for vaginal births if the care provider and the parturient woman regard a small reduction in blood loss and a small reduction in the duration of the third stage of labour as important (Weak, High)<br>• Late cord clamping (performed after 1 to 3 minutes after birth) is recommended for all births while initiating simultaneous essential newborn care (Strong, Moderate)<br>• Early cord clamping (<1 minute after birth) is not recommended unless the neonate is asphyxiated and needs to be moved immediately for resuscitation (Strong, Moderate)<br>• Controlled cord traction is the recommended method for removal of the placenta in caesarean section (Strong, Moderate)
Uterine Massage	• Sustained uterine massage is not recommended as an intervention to prevent PPH in women who have received prophylactic oxytocin. (Weak, Low)<br>• Postpartum abdominal uterine tonus assessment for early identification of uterine atony is recommended for all women (Strong, Very low)<br>• Uterine massage is recommended for the treatment of PPH (Strong, very low)<br>• The use of bimanual uterine compression is recommended as a temporizing measure until appropriate care is available for the treatment of PPH due to uterine atony after vaginal delivery. (Weak, very low)<br>• The use of uterine packing is not recommended for the treatment of PPH due to uterine atony after vaginal birth (Weak, very low)
Protocol/ Training	• The use of formal protocols by health facilities for the prevention and treatment of PPH is recommended (Weak, moderate)<br>• The use of formal protocols for referral of women to a higher level of care is recommended for health facilities (Weak, very low)<br>• The use of simulations of PPH treatment is recommended for pre-service and in-service training programmes. (Weak, very low)

1. Please *rank* the four *clinical areas of the PPH guideline* in order of importance in the context of Ethiopia at this time from 1 to 4, where 1 = first most important and 4 = fourth most important.

Clinical Area	Ranking [please write your response, a ranking of 1 to 4, directly in the box]
**Use of uterotonics**	
**Cord clamping**	
**Uterine massage**	
**Protocol/training**	

Section 3: Selection and Implementation of Recommendations within each Clinical Area in the Prevention and Treatment of PPH Guideline

In this section, you are being asked to ***select*** recommendations within each clinical area that you feel are priorities in Ethiopia at this time, and to ***rate*** how well the selected recommendations are being implemented within your individual setting and/or based on your perspective.

1. CLINICAL AREA 1: *USE OF UTEROTONICS*

From the list of recommendations presented below related to the clinical area of *use of uterotonics*, please *select* the 5 recommendations that you feel are priorities in Ethiopia at this time and *rate* your 5 selected recommendations based on how well you think these recommendations are currently being implemented at this time (on a scale from 1 to 5, where 1 = not at all and 5 = extremely well).

In this column, select your top 5 priority recommendations for the use of uterotonics, by checking the relevant boxes.

In this section, rate only your top 5 priority recommendations in terms of how well they are currently being implemented in Ethiopia, by checking the relevant boxes.

		**How well are your 5 selected recommendations currently being implemented in Ethiopia?**				
**Use of Uterotonics Recommendations (Strength, Quality)**	**Is this a priority recommendation? (select only 5)**	**1 (Not at all)**	**2**	**3**	**4**	**5 (Extremely well)**
In settings where oxytocin is unavailable, the use of other injectable uterotonics (if appropriate ergometrine/ methylergometrine or the fixed drug combination of oxytocin and ergometrine) or oral misoprostol (600 µg) is recommended. (Strong, Moderate)	☐	☐	☐	☐	☐	☐
In settings where skilled birth attendants are not present and oxytocin is unavailable, the administration of misoprostol (600 µg PO) by community health care workers and lay health workers is recommended for the prevention of PPH.(Strong, Moderate)	☐	☐	☐	☐	☐	☐

		How well are your 5 selected recommendations currently being implemented in Ethiopia?				
**Use of Uterotonics Recommendations (Strength, Quality)**	**Is this a priority recommendation? (select only 5)**	**1 (Not at all)**	**2**	**3**	**4**	**5 (Extremely well)**
The use of uterotonics for the prevention of PPH during the third stage of labour is recommended for all births (Strong, Moderate)	☐	☐	☐	☐	☐	☐
Oxytocin (10 IU, IV/IM) is the recommended uterotonic drug for the prevention of PPH (Strong, Moderate)	☐	☐	☐	☐	☐	☐
In settings where oxytocin is unavailable, the use of other injectable uterotonics (if appropriate ergometrine/ methylergometrine or the fixed drug combination of oxytocin and ergometrine) or oral misoprostol (600 µg) is recommended. (Strong, Moderate)	☐	☐	☐	☐	☐	☐
In settings where skilled birth attendants are not present and oxytocin is unavailable, the administration of misoprostol (600 µg PO) by community health care workers and lay health workers is recommended for the prevention of PPH.(Strong, Moderate)	☐	☐	☐	☐	☐	☐
Oxytocin (IV or IM) is the recommended uterotonic drug for the prevention of PPH in caesarean section (Strong, Moderate)	☐	☐	☐	☐	☐	☐
Intravenous oxytocin alone is the recommended uterotonic drug for the treatment of PPH (Strong, Moderate)	☐	☐	☐	☐	☐	☐
If intravenous oxytocin is unavailable, or if the bleeding does not respond to oxytocin, the use of intravenous ergometrine, oxytocin-ergometrine fixed dose, or a prostaglandin drug (including sublingual misoprostol, 800 µg) is recommended. (Strong, low)	☐	☐	☐	☐	☐	☐
The use of isotonic crystalloids is recommended in preference to the use of colloids for the initial intravenous fluid resuscitation of women with PPH (Strong, low)	☐	☐	☐	☐	☐	☐

Use of Uterotonics Recommendations (Strength, Quality)	Is this a priority recommendation? (select only 5)	How well are your 5 selected recommendations currently being implemented in Ethiopia? 1 (Not at all)	2	3	4	5 (Extremely well)
If bleeding does not stop in spite of treatment using uterotonics and other available conservative interventions (e.g. uterine massage, balloon tamponade), the use of surgical interventions is recommended (Strong, very low)	☐	☐	☐	☐	☐	☐
The use of ergometrine for the management of retained placenta is not recommended as this may cause tetanic uterine contractions which may delay the expulsion of the placenta. (Weak, very low)	☐	☐	☐	☐	☐	☐
Monitoring the use of uterotonics after birth for the prevention of PPH is recommended as a process indicator for programmatic evaluation (Weak, very low)	☐	☐	☐	☐	☐	☐
A single dose of antibiotics (ampicillin or first-generation cephalosporin) is recommended if manual removal of the placenta is practised. (Weak, very low)	☐	☐	☐	☐	☐	☐

2. **CLINICAL AREA 2: *CORD CLAMPING***

From the below list of recommendations related to the clinical area of *cord clamping*, please *select* the 2 recommendations that you feel are priorities in Ethiopia at this time and *rate* your 2 selected recommendations based on how well you think these recommendations are currently being implemented at this time (on a scale from 1 to 5, where 1 = not at all and 5 = extremely well).

In this column, select your top 2 priority recommendations for cord clamping, by checking the relevant boxes.

In this section, rate only your top 2 priority recommendations in terms of how well they are currently being implemented in Ethiopia, by checking the relevant boxes.

		How well are your 2 selected recommendations currently being implemented in Ethiopia?				
**Cord Clamping Recommendations (Strength, Quality)**	**Is this a priority recommendation? (select only 2)**	**1 (Not at all)**	**2**	**3**	**4**	**5 (Extremely well)**
In settings where skilled birth attendants are available, CCT is recommended for vaginal births if the care provider and the parturient woman regard a small reduction in blood loss and a small reduction in the duration of the third stage of labour as important (Weak, High)	☐	☐	☐	☐	☐	☐
Late cord clamping (performed after 1 to 3 minutes after birth) is recommended for all births while initiating simultaneous essential newborn care (Strong, Moderate)	☐	☐	☐	☐	☐	☐
Early cord clamping (<1 minute after birth) is not recommended unless the neonate is asphyxiated and needs to be moved immediately for resuscitation (Strong, Moderate)	☐	☐	☐	☐	☐	☐
Controlled cord traction is the recommended method for removal of the placenta in caesarean section (Strong, Moderate)	☐	☐	☐	☐	☐	☐

3. **CLINICAL AREA 3: *UTERINE MASSAGE***

From the below list of recommendations related to the clinical area of *uterine massage*, please *select* the 2 recommendations that you feel are priorities in Ethiopia at this time and *rate* your 2 selected recommendations based on how well you think these recommendations are currently being implemented at this time (on a scale from 1 to 5, where 1 = not at all and 5 = extremely well).

In this column, select your top 2 priority recommendations for uterine massage, by checking the relevant boxes.

In this section, rate only your top 2 priority recommendations in terms of how well they are currently being implemented in Ethiopia, by checking the relevant boxes.

		**How well are your 2 selected recommendations currently being implemented in Ethiopia?**				
**Uterine Massage Recommendations (Strength, Quality)**	**Is this a priority recommendation? (select only 2)**	**1 (Not at all)**	**2**	**3**	**4**	**5 (Extremely well)**
Sustained uterine massage is not recommended as an intervention to prevent PPH in women who have received prophylactic oxytocin. (Weak, Low)						
Postpartum abdominal uterine tonus assessment for early identification of uterine atony is recommended for all women (Strong, Very low)						
Uterine massage is recommended for the treatment of PPH (Strong, very low)						
The use of bimanual uterine compression is recommended as a temporizing measure until appropriate care is available for the treatment of PPH due to uterine atony after vaginal delivery. (Weak, very low)						
The use of uterine packing is not recommended for the treatment of PPH due to uterine atony after vaginal birth (Weak, very low)						

4. **AREA 4: *PROTOCOL/TRAINING***

From the below list of recommendations related to the area of *protocol/training*, please *select* the 2 recommendations that you feel are priorities in Ethiopia at this time and *rate* your 2 selected recommendations based on how well you think these recommendations are currently being implemented at this time (on a scale from 1 to 5, where 1 = not at all and 5 = extremely well).

In this column, select your top 2 priority recommendations for protocol/training, by checking the relevant boxes.

In this section, rate only your top 2 priority recommendations in terms of how well they are currently being implemented in Ethiopia, by checking the relevant boxes.

**Protocol/Training Recommendations (Strength, Quality)**	**Is this a priority recommendation? (select only 2)**	**How well are your 2 selected recommendations currently being implemented in Ethiopia?** 1 (Not at all)	2	3	4	5 (Extremely well)
The use of formal protocols by health facilities for the prevention and treatment of PPH is recommended (Weak, moderate)	☐	☐	☐	☐	☐	☐
The use of formal protocols for referral of women to a higher level of care is recommended for health facilities (Weak, very low)	☐	☐	☐	☐	☐	☐
The use of simulations of PPH treatment is recommended for pre-service and in-service training programmes. (Weak, very low)	☐	☐	☐	☐	☐	☐

5. Is there anything else about guideline implementation in Ethiopia generally, or implementation of the PPH guidelines in Ethiopia specifically, that you would like to share (e.g., related initiatives, working groups/committees, related strategies/country plans, barriers, facilitators, etc.)?

Thank you very much for participating in this survey.

APPENDIX B: FOCUS GROUP DISCUSSION GUIDES

Table A. Focus Group Discussion: Mixed Group
Day 1: Focus Group Discussion
Duration: (1:45-3:30 p.m.)

Role of facilitators:

- To objectively gather data from multiple participants on a specific topic
- Your transcribed words are not included in analysis
- You are a receiver, not a transmitter
- You are not an expert on the topic
- Refer to the time stamps on the guide to help you stay on track
- Make references to the ground rules to avoid any disruptions
- Ensure audio recorders are placed in optimal locations and that the participant speaking is holding the audio recorder whenever possible

Instructions for facilitators:

- *Welcome and introductions*
- *Collect signed consent forms*
- *Review Focus Group Ground Rules*
- *Remind participants to speak into the audio recorder, as much as possible*
- *Review WHO guideline summary on the Prevention and Treatment of Post-Partum Haemorrhage*

Questions/instructions for participants

Legend:

- **Questions** and *Instructions* are indicated as such in the left hand column. *Instructions* are meant to be directions for the participants, given to them by the facilitator.
- Directions for the facilitator are indicated in *italics* in the body of the text of the second column.

Instructions	If you haven't already, please take a few minutes to review the summary of the WHO guideline, which is available in your Workshop Package.<br><br>*Note: ensure that participants have had a chance to read the summaries.*<br><br>I would like to draw your attention to the recommendations of this guideline.
**Question 1**	Keeping in mind the context of maternal and newborn health in Ethiopia at this time, what three recommendations are the most important to implement for the Prevention and Treatment of Post-Partum Haemorrhage (PPH) guideline?<br><br>Probes (*note*: *use probes if the participants did not provide enough information in their responses to the above question*)<br>• Why did you select those specific guideline recommendations?<br>• What factors did you consider when selecting the guideline recommendations?
**Question 2**	Thinking about the top three recommendations for the guideline as a whole, what do you think are the potential barriers or challenges to implementing these guideline recommendations in Ethiopia?<br><br>Probes<br>• What are some of the barriers or challenges at the systems level? *Examples include funding, policy, health care structure, geography, current cultural and political climate in Ethiopia, etc.*

<table>
<tr><td></td><td>

What are some of the barriers or challenges at the level of the health care provider?
Examples include skills, attitudes/beliefs, leadership, interprofessional working climate, etc.
What are some of the barriers or challenges at the level of the patients and communities?
Examples include cultural beliefs, health seeking behaviours, preferences for care, etc.

Note: If participants want to discuss specific barriers to each of the recommendations individually, it is possible to discuss 3 – 5 specific recommendations in total (i.e. across all guidelines), but ensure that you bring the conversation back to <u>common</u> barriers across all recommendations as there is little time to discuss each recommendation individually. It is likely that barriers/challenges will overlap across all recommendations.
</td></tr>
<tr><td>Question 3</td><td>
Again, thinking about the top three recommendations for the guideline as a whole, what do you think are the potential facilitators that could aid in the implementation of the guideline?

<u>Probes</u>

What are some of the facilitators at the systems level?
Examples include alignment with current initiatives, political turnover/opportunity, updating health training curricula, etc.
What are some of the facilitators at the level of the health care provider?
Examples include champions at each clinical level, strong leadership, reward systems/positive reinforcement, training, etc.
What are some of the facilitators at the level of the patients and communities?
Examples include cultural beliefs, health seeking behaviours, preferences for care, etc.

Note: If participants want to discuss specific facilitators to each of the recommendations individually, it is possible to discuss 3 – 5 specific recommendations in total (i.e. across all guidelines), but ensure that you bring the conversation back to <u>common</u> facilitators across all recommendations as there is little time to discuss each recommendation individually. It is likely that facilitators will overlap across all recommendations.
</td></tr>
<tr><td>Question 4</td><td>
Do you feel that there is sufficient readiness and buy-in in Ethiopia to implement these guideline recommendations?

<u>Probes</u>

If yes, please describe readiness for change at each level: health systems, providers, and patients/communities.
If no, why not? What would be required to make Ethiopia more prepared to implement these guideline recommendations?

</td></tr>
<tr><td>Question 5</td><td>Do you have any additional suggestions that could help with the implementation of the WHO guideline on PPH?</td></tr>
<tr><td>Question 6</td><td>Before we wrap up today’s discussion, is there anything else that anyone would like to add?</td></tr>
<tr><td colspan="2">Thank participants and wrap up</td></tr>
</table>

<table>
<tr><td colspan="2">Table B. Focus Group Discussions: Midwives
Day 1: Focus Group Discussion
Duration: (1:45-3:30 p.m.)</td></tr>
<tr><td colspan="2">Role of facilitators:

• To objectively gather data from multiple participants on a specific topic
• Your transcribed words are not included in analysis
• You are a receiver, not a transmitter
• You are not an expert on the topic
• Refer to the time stamps on the guide to help you stay on track
• Make references to the ground rules to avoid any disruptions
• Ensure audio recorders are placed in optimal locations and that the participant speaking is holding the audio recorder whenever possible</td></tr>
<tr><td colspan="2">Instructions for facilitators:

• Welcome and introductions
• Collect signed consent forms
• Review Focus Group Ground Rules
• Remind participants to speak into the audio recorder, as much as possible
• Review WHO guideline summary on the Prevention and Treatment of Post-Partum Haemorrhage</td></tr>
<tr><td colspan="2">Questions/instructions for participants

Legend:
• Questions and Instructions are indicated as such in the left hand column. Instructions are meant to be directions for the <u>participants</u>, given to them by the facilitator.
• Directions for the facilitator are indicated in italics in the body of the text of the second column.</td></tr>
<tr><td>Instructions</td><td>If you haven’t already, please take a few minutes to review the summary of the WHO guideline, which is available in your Workshop Package.

Note: ensure that participants have had a chance to read the summaries.

I would like to draw your attention to the recommendations of this guideline.</td></tr>
<tr><td>Question 1</td><td>Keeping in mind the context of maternal and newborn health in Ethiopia at this time, what <u>three</u> recommendations are the most important to implement for the Prevention and Treatment of Post-Partum Haemorrhage (PPH) guideline?

<u>Probes </u>(note: use probes if the participants did not provide enough information in their responses to the above question)
• Why did you select those specific guideline recommendations?
• What factors did you consider when selecting the guideline recommendations?</td></tr>
<tr><td>Question 2</td><td>Thinking about the top three recommendations for the guideline as a whole, what do you think are the potential barriers or challenges to implementing these guideline recommendations in Ethiopia?

<u>Probes</u>
• What are some of the barriers or challenges at the systems level?
Examples include funding, policy, health care structure, geography, current cultural and political climate in Ethiopia, etc.
• What are some of the barriers or challenges at the level of the health care provider?</td></tr>
</table>

	Examples include skills, attitudes/beliefs, leadership, interprofessional working climate, etc.<br>• What are some of the barriers or challenges at the level of the patients and communities?<br>*Examples include cultural beliefs, health seeking behaviours, preferences for care, etc.*<br><br>*Note: If participants want to discuss specific barriers to each of the recommendations individually, it is possible to discuss 3 – 5 specific recommendations in total (i.e. across all guidelines), but ensure that you bring the conversation back to common barriers across all recommendations as there is little time to discuss each recommendation individually. It is likely that barriers/challenges will overlap across all recommendations.*
**Question 3**	Again, thinking about the top three recommendations for the guideline as a whole, what do you think are the potential facilitators that could aid in the implementation of these guidelines?<br><br>Probes<br>• What are some of the facilitators at the systems level?<br>*Examples include alignment with current initiatives, political turnover/opportunity, updating health training curricula, etc.*<br>• What are some of the facilitators at the level of the health care provider?<br>*Examples include champions at each clinical level, strong leadership, reward systems/positive reinforcement, training, etc.*<br>• What are some of the facilitators at the level of the patients and communities?<br>*Examples include cultural beliefs, health seeking behaviours, preferences for care, etc.*<br><br>*Note: If participants want to discuss specific facilitators to each of the recommendations individually, it is possible to discuss 3 – 5 specific recommendations in total (i.e. across all guidelines), but ensure that you bring the conversation back to common facilitators across all recommendations as there is little time to discuss each recommendation individually. It is likely that facilitators will overlap across all recommendations.*
**Question 4**	Do you have any additional suggestions that could help with the implementation of the WHO guideline on the Prevention and Treatment of PPH?<br><br>Is there anything else that anyone would like to add?
**Thank participants and wrap up**	

APPENDIX C: PRE-WORKSHOP SURVEY FINDINGS ON THE SELECTION AND IMPLEMENTATION OF PRIORITY RECOMMENDATIONS IN THE PREVENTION AND TREATMENT OF PPH GUIDELINE

Table A. Top 5 Recommendations related to the clinical area of 'use of uterotonics'

**Recommendations related to the clinical area of Use of Uterotonics**	**n** (n=53)	**%**	**Perceived level of Implementation Score* [median (IQR 25th, 75th)]**
The use of uterotonics for the prevention of PPH during the third stage of labour is recommended for all births (Strong, Moderate).	43	81.1	5 (4, 5)
Oxytocin (10 IU, IV/IM) is the recommended uterotonic drug for the prevention of PPH (Strong, Moderate).	35	66.0	5 (4, 5)
In settings where oxytocin is unavailable, the use of other injectable uterotonics (if appropriate ergometrine/ methylergometrine or the fixed drug combination of oxytocin and ergometrine) or oral misoprostol (600 µg) is recommended (Strong, Moderate).	31	58.5	4 (4, 5)
In settings where skilled birth attendants are not present and oxytocin is unavailable, the administration of misoprostol (600 µg PO) by community health care workers and lay health workers is recommended for the prevention of PPH (Strong, Moderate).	27	50.9	4 (3, 4)
If bleeding does not stop in spite of treatment using uterotonics and other available conservative interventions (e.g. uterine massage, balloon tamponade), the use of surgical interventions is recommended (Strong, very low).	24	45.3	4 (3, 5)

Table B. Top 2 Recommendations related to the clinical area of 'cord clamping'

**Recommendations related to the clinical area of Cord Clamping**	**n** (n=53)	**%**	**Perceived level of Implementation Score* [median (IQR 25th, 75th)]**
In settings where skilled birth attendants are available, CCT is recommended for vaginal births if the care provider and the parturient woman regard a small reduction in blood loss and a small reduction in the duration of the third stage of labour as important (Weak, High).	36	67.9	5 (4, 5)
Early cord clamping (<1 minute after birth) is not recommended unless the neonate is asphyxiated and needs to be moved immediately for resuscitation (Strong, Moderate).	28	52.8	5 (4, 5)

*The perceived level of implementation score represents respondents' perception in terms of how the recommendation is currently being implemented in the Ethiopian context from a scale of 1 (not at all) to 5 (extremely well).

Table C. Top 2 Recommendations related to the clinical area of 'uterine massage'

**Recommendations related to the clinical area of Uterine Massage**	**n** (n=53)	**%**	**Perceived level of Implementation Score* [median (IQR 25th, 75th)]**
Postpartum abdominal uterine tonus assessment for early identification of uterine atony is recommended for all women (Strong, Very low).	43	81.1	4 (4, 5)
Uterine massage is recommended for the treatment of PPH (Strong, very low).	28	52.8	5 (4, 5)

Table D. Top 2 Recommendations related to the area of 'protocols/training'

**Recommendations related to the area of Protocol/Training**	**n** (n=51)	**%**	**Perceived level of Implementation Score* [median (IQR 25th, 75th)]**
The use of formal protocols by health facilities for the prevention and treatment of PPH is recommended (Weak, moderate).	48	94.1	4 (3, 5)
The use of simulations of PPH treatment is recommended for pre-service and in-service training programmes (Weak, very low).	28	54.9	3 (2, 4)

*The perceived level of implementation score represents respondents' perception in terms of how the recommendation is currently being implemented in the Ethiopian context from a scale of 1 (not at all) to 5 (extremely well).

APPENDIX D: MEDIAN SCORE AND INTERQUARTILE RANGE (IQR) FOR FEASIBILITY RANKINGS FOR RECOMMENDATIONS OF THE PREVENTION AND TREATMENT OF PPH GUIDELINE

	**Recommendation**	**Score [median (IQR 25th, 75th)]**
**Recommendations for the prevention of PPH**	The use of uterotonics for the prevention of PPH during the third stage of labour is recommended for all births.	9 (8, 9)
	Oxytocin (10 IU, IV/IM) is the recommended uterotonic drug for the prevention of PPH.	8 (8, 9)
	In settings where skilled birth attendants are not present and oxytocin is unavailable, the administration of misoprostol (600 µg PO) by community health care workers and lay health workers is recommended for the prevention of PPH.	8 (8, 9)*
	Late cord clamping (performed after 1 to 3 minutes after birth) is recommended for all births while initiating simultaneous essential newborn care.	9 (8, 9) *
	Postpartum abdominal uterine tonus assessment for early identification of uterine atony is recommended for all women.	9 (8, 9)
	If intravenous oxytocin is unavailable, or if the bleeding does not respond to oxytocin, the use of intravenous ergometrine, oxytocin-ergometrine fixed dose, or a prostaglandin drug (including sublingual misoprostol, 800 µg) is recommended.	7 (7, 8)
	Uterine massage is recommended for the treatment of PPH.	9 (8, 9)
**Recommendations for treatment of PPH**	The use of bimanual uterine compression is recommended as a temporizing measure until appropriate care is available for the treatment of PPH due to uterine atony after vaginal delivery.	7 (7, 8)*
	The use of uterine packing is not recommended for the treatment of PPH due to uterine atony after vaginal birth.	9 (8, 9)*
	A single dose of antibiotics (ampicillin or first-generation cephalosporin) is recommended if manual removal of the placenta is practiced.	8 (8, 9)
	The use of simulations of PPH treatment is recommended for pre-service and in-service training programmes.	7 (7, 8)*

***Note:** These recommendations were re-ranked after further consideration. The re-ranked data is displayed in the table.
